# Identification of tooth traces from a Cretaceous (Maastrichtian) *Edmontosaurus annectens* bonebed in the Lance Formation, Wyoming, U.S.A.

**DOI:** 10.1371/journal.pone.0351939

**Published:** 2026-07-15

**Authors:** Bethania C. T. Siviero, Elizabeth Rega, Matthew A. McLain, Leonard R. Brand, David Nelsen, Art V. Chadwick

**Affiliations:** 1 Department of Pathology and Human Anatomy, Loma Linda University, Loma Linda, California, United States of America; 2 Department of Earth and Biological Sciences, Loma Linda University, Loma Linda, California, United States of America; 3 Department of Medical Anatomical Sciences, Western University of Health Sciences, Pomona, California, United States of America; 4 Department of Biological and Physical Sciences, The Master’s University, Santa Clarita, California, United States of America; 5 Department of Biology, Southern Adventist University, Chattanooga, Tennessee, United States of America; 6 Department of Biology, Southwestern Adventist University, Keene, Texas, United States of America; University of Gothenburg: Goteborgs Universitet, SWEDEN

## Abstract

Identifying the origin of perforating lesions on fossil bone is often difficult, and many are considered tooth traces, in spite of more likely and more parsimonious etiologies. Much of this confusion stems from tooth trace criteria that are ambiguous when the context for the lesions is not considered. Mistaken identification of tooth traces has led to misleading interpretations of animal behavior. This study of tooth traces on fossil bones critically reviews previous criteria and applies them to assessing bones from an *Edmontosaurus annectens* bonebed within the Lance Formation, Wyoming, USA. Of the 3013 bones examined, thirteen bones had features indicative of tooth traces based on gross appearance. Of these, one bone had perforations determined to have a different etiology. Twelve bones had traces attributed to tooth marks, including four bones with *Knethichnus parallelum* and *Linichnus serratus* ichnotaxa. *Tyrannosaurus rex* was identified as the likely inflictor of traces attributable to both ichnotaxa, by comparison of denticle density of carnivore teeth within the bonebed with striation/serration density of the traces. The importance of context in the analysis of perforating lesions on fossil bones is shown through the mistaken identification of features such as neurovasculature foramina and lesions associated with pathology as tooth traces. This study contributes to the literature on biting behavior and refines the criteria used to identify perforations caused by bite marks. The application of these refined criteria also proved useful in accurately identifying tooth traces on bone. This, in turn, enhances the guidelines for recognizing perforations as tooth traces and encourages further research on this topic.

## Introduction

Reports of dinosaur bones with tooth traces often attract widespread attention, because of their association with animal behavior. Tooth traces in fossil bones have prompted discussions and have contributed to a better understanding of interspecific interactions in extinct animals [1,2]. Noto et al. [3] examined limb bones from ornithopods with markings attributed to tooth traces. The morphology and arrangement of marks were comparable with the dental remains of an associated large crocodyliform, which was suggested to have been the inflictor. Lei et al. [4] also reported the widespread occurrence of tooth traces associated with theropods on sauropod fossil bones, suggesting the preferential feeding of sauropod carcasses by theropods. Currie and Jacobsen [5] described an occasion of a bite mark with an embedded tooth from a velociraptorine theropod on an azhdarchid pterosaur tibia. The authors suggested theropod scavenging on a pterosaur carcass because of the size difference between the animals. Literature reports describe numerous additional examples of interspecific interactions evidenced by tooth marks, including crocodyliform bite traces on dinosaur bones [6–8], on turtle shells [9], mammalian bite marks on dinosaurs [10] and among various different dinosaur species [4,11–16]. Tooth trace evidence in fossil bones has also deepened our understanding of behavior within species. Previous reports of tooth traces and associated scars on caudal vertebrae of the crocodylomorph *Baurusuchus pachecoi*, with tooth morphology matching that of the affected bones, suggest attacks by individuals of the same species, likely reflecting intraspecific interaction related to social behavior [17]. In addition, studies documenting the presence and frequency of bite marks on theropod dinosaur skulls also point to intraspecific interaction, potentially associated with intersexual display [18]. Cannibalistic behavior amongst dinosaurs has also been reported [19,20] including cases of *Tyrannosaurus rex* cannibalism [21–23] and other theropods [19].

Over the years, there have been several attempts to determine evidence-based criteria for bones with tooth traces [24–27]. The criteria are based on description and classification of bone perforations interpreted as bite marks or tooth traces. It was first identified and described by Binford [24]. There are four basic types of tooth traces: 1) pit; 2) puncture; 3) score; and 4) furrow. While these four types are still in use today, their definitions have changed with the passage of time. Although most of definitions came from the observations of mammalian carnivores using either canines or carnassials to mark/perforate bone, these definitions for tooth traces can also be used with other carnivorous animals such as dinosaurs, crocodilians and sharks, due to similar functional characteristics of flesh-tearing or bone-crushing teeth and underlying similarities of bone structures.

Tooth traces have themselves been described and classified as ichnotaxa, suggesting traces of ancient behavior. Mikuláš, et al. [28] introduced the ichnotaxon *Nihilichnus nihilicus* to describe punch-hole or puncture traces. Jacobsen and Bromley [29] named two additional tooth trace ichnotaxa based on dinosaur tooth trace specimens: *Linichnus serratus* and *Knethichnus parallelum*. *Linichnus serratus* is characterized by a curved score with a U- or V-shaped geometry in cross-section and a serrated morphology. *Knethichnus parallelum* consists of a series of parallel grooves, sometimes extending from an initial groove, caused by the denticles of a ziphodont tooth dragging along the bone surface [29]. Carnivorans (mammalian carnivores of the order Carnivora), odontocetes, crocodilians, toothed theropod dinosaurs, sharks, and other organisms are capable of producing *Nihilichnus nihilicus* [28]. However, only carnivores with ziphodont teeth, such as theropods, varanids, and sharks are capable of producing *Knethichnus parallelum* and *Linichnus serratus* [29]. Thus, it is helpful to compare different ichnotaxa to determine perpetrator.

Not all features that resemble pits, punctures, scores, or furrows are true tooth traces, as similar marks can result from non-biting processes (see S1 Table for a review of the criteria). For example, the perforations on the left surangular of *Tyrannosaurus rex* FMNH PR 2081 (“Sue”) were once interpreted as penetrating bite trauma [30], other reports proposed these lesions to be the result of a biogenic bone infection that affected the mandibular region of this animal [31–33]. Wolf et al. [32] described the perforations as multiple round erosive lesions with smooth-edges and associated bone remodeling. Similar features were also reported as occurring on the mandibles of other tyrannosaurid and archosaur specimens [32,33]. A conclusive analysis of the lesions’ shape and arrangement as well as their contextual location and frequency in other similar animals suggested a bacterial or protozoan infection that affected tyrannosaurids as a result of intraspecific behavior with other infested animals [32]. This highlights the need to evaluate bone context carefully: pitting or puncture-like features may also arise from other processes such as taphonomic erosion [34], normal anatomical structures (such as neurovascular foramina or cloaca) [35] certain joint diseases, or erosive infections [31,35]. In addition, advanced healing after an actual bite can obscure or alter original tooth traces, further complicating identification. Thus, extraordinary claims of tooth traces on a bone should be subject to rigorous examination because, when misidentified and exaggerated, they have the potential to lead to erroneous conclusions pertaining to intra- or interspecific animal behavior.

In this study, we examined fossil bones from a monodominant *Edmontosaurus annectens* bonebed within the Maastrichtian Lance Formation in eastern Wyoming [36] for perforations indicative of tooth traces. We rigorously compared the perforations with previously published identification and classification for tooth traces including tooth trace ichnotaxa. Context for these perforations were also examined to determine if it was a legitimate tooth trace or an ambiguous perforation of different etiology. Consequently, we revised and refined the criteria for identifying tooth traces and expanded our discussion of other types of bone perforations that may be mistaken for them.

## Materials and methods

We examined approximately 3013 dinosaur bones (mostly *E. annectens*) initially found and excavated from a bonebed near the Hanson Ranch Station (HRS) in Roxson, northeastern Wyoming [36] between 1997−2017. Bones were prepared and stored at the Dinosaur Research Center located at Southwestern Adventist University (SWAU) in Keene, TX. Fossils from the two largest quarries, North and South quarries [36], in the same bonebed were subject to screening by the first author. Marks or perforations that potentially indicated tooth traces were further examined with a magnifier loupe (Bausch & Lomb 10X), Dino-Lite Edge digital microscope, and stereo microscope. Using the previously reported definitions and descriptions for tooth traces (S1 Fig), we identified and classified the bones with potential tooth traces. Pictures of specimens were taken with a Canon EOS D30 camera on a Beseler CS-21 copy stand cameral platform with external lighting for better image contrast. For images that required thin/narrow focal plane and extended depth of field at high resolution, we used photo stacking Helicon Software and Adobe Photoshop Lightroom CC. Specimens with possible tooth traces that were ambiguous were further investigated with a CT scan taken at the Loma Linda University Medical Center.

Additional photographic images of all HRS labeled specimens reported in this paper are available for viewing in the SWAU fossil catalog available at https://fossil.swau.edu. The associated accession numbers to the HRS fossil bones directly discussed herein are: HRS05499, HRS09477, HRS03161, HRS09551, HRS03387, HRS09954, HRS01295, HRS03650, HRS00428, HRS09830, HRS00473, HRS13582, HRS10076. Other HRS fossil bone numbers mentioned in this manuscript are: HRS07948, HRS07804, HRS07887, HRS01254, HRS05808. No permits were required for the described study, which complied with all relevant regulations.

Some of the bones manifested markings attributable to *Knethichnus parallelum* and *Linichnus serratus* ichnotaxa [29]. In order to attempt identification of the probable tooth trace maker, we selected the bones with *Knethichnus parallelum* traces and measured the spacing of the parallel striations to determine denticle density (number of denticles per 2 mm). We used a 10 mm microruler (TDI International, Inc.) under the stereo microscope (Nikon SMZ-10A) for this measurement. We selected different ziphodont teeth from the associated bonebed to compare denticle densities to the striation density from the *Knethichnus parallelum* traces. We measured the number of denticles per 2 mm under the Dino-Lite microscope*.* Forty different teeth (ten specimens per selected species) were measured for mesial and distal denticular density (S2 Fig) and give the density average for each species.

## Results

**Fig 1:**
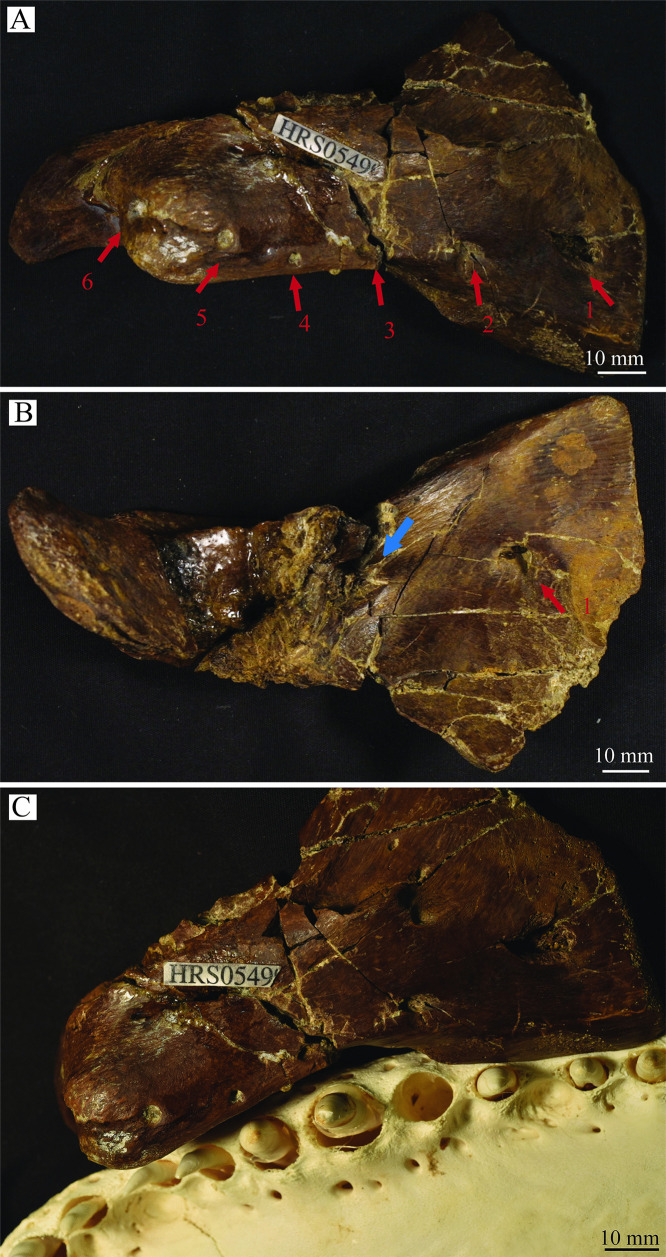
Bone perforation on surangular HRS05499. **A)** Lateral view of surangular. **B)** Medial view of surangular. Red arrows indicate different bone perforations and blue arrow indicates a different type of perforation. **C)** Comparison of bone perforations alignment and spacing of surangular with the tooth alignment and spacing of a modern crocodile maxilla.

Out of the 3013 specimens examined from the largely monodominant *E. annectens* bonebed, we identified only thirteen bones with perforations that resembled tooth traces. Based on the criteria for tooth traces from previous works (S1 Fig), and the additional attention to context provided by the CT scan images, we found that one specific specimen, the surangular HRS05499, out of the thirteen fossils, had questionable tooth trace-like perforations (Fig 1). Twelve specimens manifested tooth traces: five ribs, five vertebrae, one radius, and one ulna. The lesions were classified into types of tooth traces: pit, puncture, score, and furrow. While some tooth traces were easily identified and classified, others were classified only because of their context and association with other traces on the same bone. From the twelve specimens with tooth traces, four specimens manifested patterns attributable to a named ichnotaxa. What follows is a description of each specimen and its associated perforation(s).

### Questionable perforations

We examined a surangular fossil HRS05499 (Fig 1) with perforations/lesions resembling tooth traces. However, the morphology and anatomical location for the “lesions”, as well as their placement in the bone, important when considering tooth trace, were ambiguous. Thus, we conducted further analysis to determine the diagnosis and classification of these perforations.

**Fig 2:**
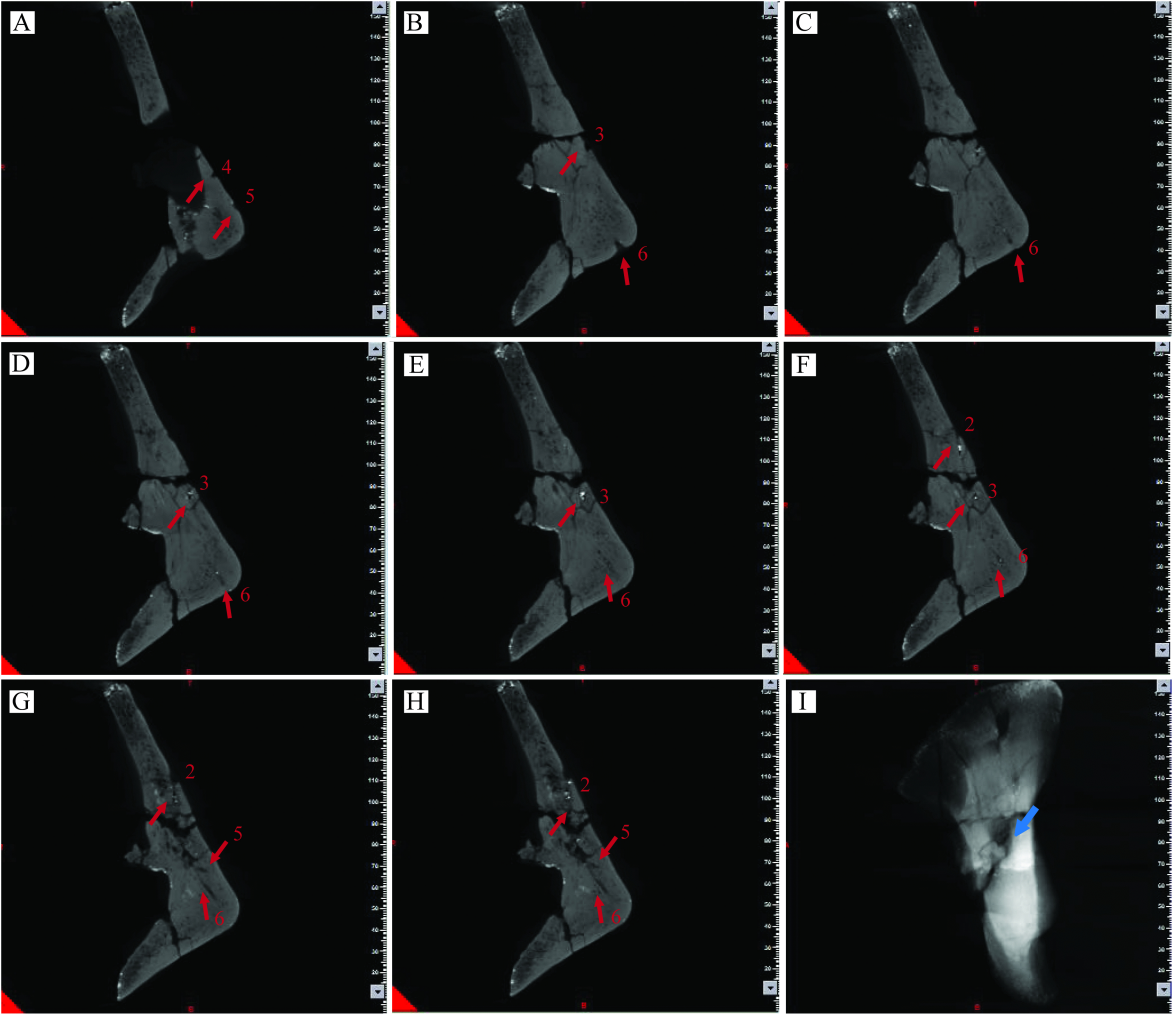
CT scanning of surangular HRS05499. **A-H)** Sequential images in coronal view (due to crossing angle of perforations). Numbers indicating the perforations correspond to Fig 1A and 1B. Red arrows indicate perforations continuing deep into the bone. **I)** Rostral view of bone. Blue arrow corresponding to blue arrow on Fig 1B, indicates different types of perforation on which several of the smaller ones (red arrows) connect.

47 surangular specimens of *E. annectens* were identified within the bonebed. However, the left surangular HRS05499 is distinct in the overall shape from all the other surangulars found in the bonebed (Figs 1A and 1B). Also, unlike the other *E. annectens* surangulars, the HRS05499 has several polygonal perforations, resembling pits and punctures caused by bite marks from conical teeth. Shed indeterminate eusuchian crocodilian teeth (likely from *Borealosuchus sternbergii*) are found within the bonebed. These teeth are conical in shape with no serration. Preliminary analysis of linear arrangement, spacing, and conical shape of the perforations matched with the tooth arrangement and spacing from a modern alligator maxilla (Fig 1C). However, the smooth edges and indetermined depth in several of the perforations are atypical. CT images reveal (following from lateral (rostral) to medial (lingual); Fig 2) that each of the perforations continues into the bone at different angles before collecting into a common area (Fig 2I), typical for bone foramina. Thus, we identified these perforations as foramen within the surangular. Although the general shape of HRS05499 shares some similarities with surangulars of *E. annectens*, its overall morphology is distinct. Accordingly, we compared this specimen with surangulars from various ceratopsid taxa, given the occasional occurrence of ceratopsid material within the bonebed, using comparative collections at the Raymond M. Alf Museum (RAM) and the Black Hills Institute of Geological Research, Inc. (BHI). These comparisons revealed a close correspondence in overall shape. However, the presence and arrangement of foramina remained non-diagnostic until a very similar match was identified with surangular specimen BHI 4772, attributed to a subadult *Torosaurus latus* (“Billy”) (S3 Fig). Although larger than our specimen, the positioning, alignment and spacing of the foramina on the two specimens are nearly identical. Thus, based on the arrangement, depth, and smooth edges of the perforations, and comparisons with other specimens, the perforations from the surangular HRS05499 are normal foramina found in a triceratopsini surangular.

### Tooth trace lesions

#### Ribs.

**Fig 3:**
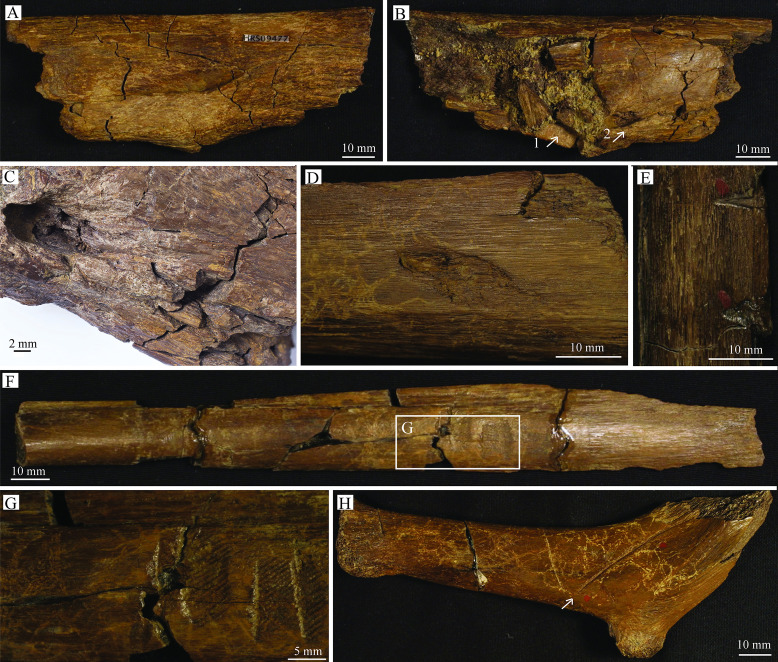
Tooth trace types on rib specimens. **A)** Prominent furrow tooth trace on rib fragment HRS09477. **B)** Two puncture traces on rib fragment HRS09477 indicated by arrows on the opposing side of a furrow on Fig 3A. **C)** Magnified image of the puncture on HRS09477 indicated by arrow 2 on Fig 3B, indicating the pull and drag from the bite. **D)** Rib fragment HRS03161 with puncture and associated drag from the bite. **E)** Rib fragment HRS09551 with parallel scores. **F)** Rib HRS03387 with prominent scores and associated tooth trace ichnotaxon *Knethichnus parallelum*. **G)** Magnification of area indicated by the white box on Fig 3F featuring prominent and detailed parallel *Knethichnus parallelum* traces*.* **H)** Rib HRS09954 with a long score trace indicated by the arrow.

Rib fragment HRS09477 has three tooth trace types: a large furrow on one surface and two punctures on the opposing surface (Fig 3A and 3B). Drag marks from the bite are associated with the puncture identified as #2 in Fig 3B and 3C. Ribs HRS03161, HRS09551 and HRS03387 have scores on the surface along their length. One rib fragment (HRS03161) features an initial bite puncture with associated score from tooth drag on the bone surface (Fig 3D). Specimen HRS09551 has two parallel aligned scores on the edge/surface of the rib fragment (Fig 3E), and specimen HRS03387 has three closely associated parallel scores on its midrib surface with the same orientation for the markings (Fig 3F and 3G). These scores also represent examples of the ichnotaxa *Knethichnus parallelum*. Lastly, rib HRS09954 has a well-defined long score on its proximal end surface (Fig 3H).

#### Vertebrae.

**Fig 4:**
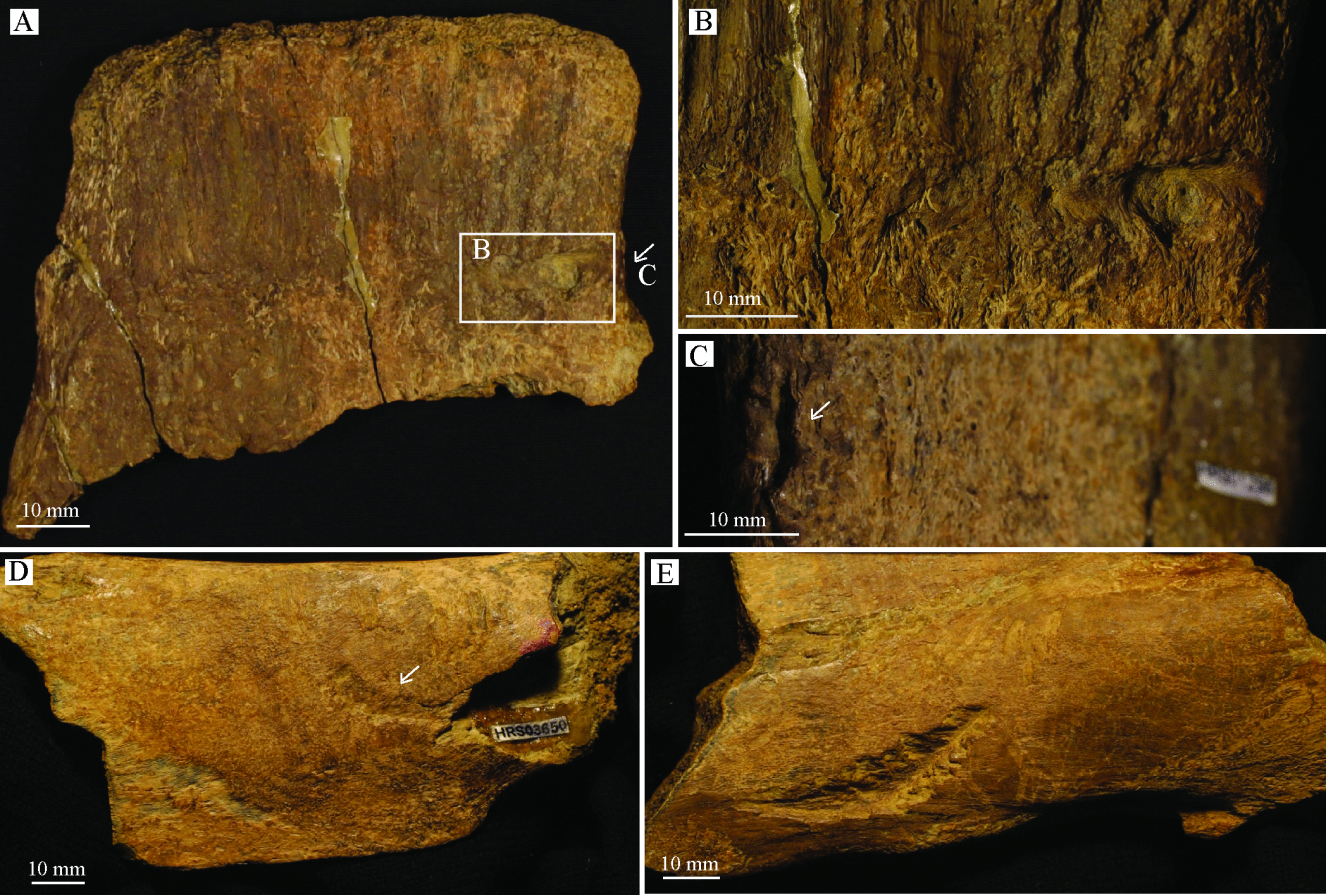
Tooth trace types on neural spine fragments. **A)** Punctures on opposing surfaces in neural spine HRS01295. The image only depicts one of the punctures indicated by the white box. An arrow indicates the location of the other puncture not visible in the image. **B)** A magnified view of the puncture within the white box area on Fig 4A. **C)** A view of the puncture opposing the puncture on Fig 4B and indicated by arrow on Fig 4A. **D)** Neural spine fragment HRS03650 with a score indicated by an arrow. **E)** A view of the opposing surface from HRS03650 (Fig 4D) shows a deep and curved score with associated tooth trace ichnotaxon *Linichnus serratus*.

**Fig 5:**
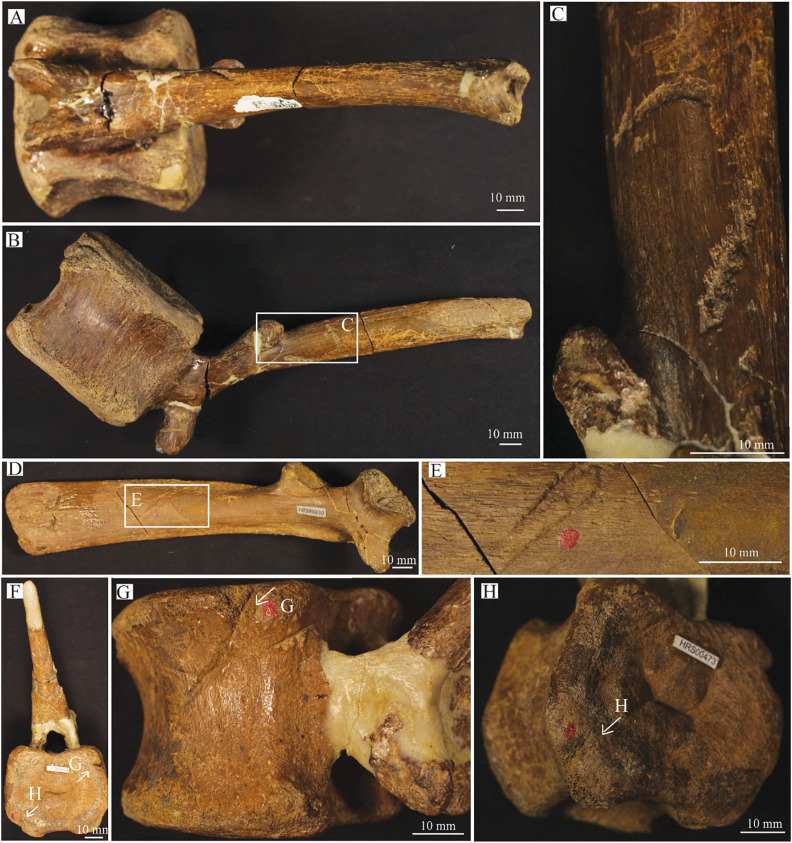
Tooth trace types on caudal vertebrae. **A)** Distal caudal vertebra HRS00428 (from middle-distal region of tail) with the distal end of the neural spine missing. Note bone proliferation on the distal neural spine. **B)** HRS00428 with curved scores on the neural spine associated with the tooth trace ichnotaxon *Linichnus serratus*. **C)** Magnification of the scores from the white box on Fig 5B depicting details of the prominent *Linichnus serratus* traces. **D)** Neural spine HRS09830 from a caudal vertebra (middle-distal region of tail) with scores within the white box area. **E)** Magnification of the scores associated with the white box from Fig 5D. **F)** Distal caudal vertebra HRS00473 with arrows indicating tooth traces on the centrum. **G)** Score on HRS00473 associated with arrow “G” on Fig 5F. Note another score closer to the junction of the centrum with the neural spine. **H)** Puncture on the centrum HRS00473 associated with arrow “H” on Fig 5F.

The tooth traces on the vertebrae are often located on the neural spine. Neural spine fragment HRS01295 has punctures on opposing surfaces (Fig 4A–4C), and one of the punctures shows a drag mark (Fig 4A and 4B). Neural spine fragment HRS03650 has a pronounced, curved, deep score with another shallower score on the opposing surface (Fig 4D and 4E). The neural spine from caudal vertebra HRS00428 (Fig 5A) has its distal end missing and associated reactive bony growth. Fig 5B and 5C also show two curved scores with serrations on the left surface of the caudal vertebra HRS00428. Neural spine HRS09830 has two parallel scores on the right surface (Fig 5D and 5E). Unlike the other tooth traces found on neural spines, the caudal vertebra HRS00473 has three different tooth traces located on its centrum (Fig 5F–5H), two scores and one puncture on the opposing side.

#### Radius and ulna.

**Fig 6:**
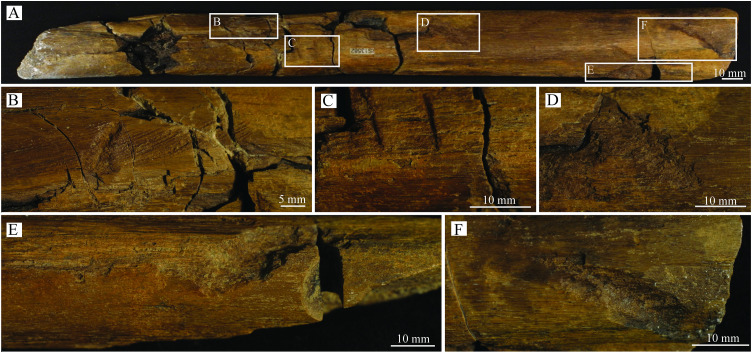
Tooth traces on radius HRS13582. **A)** Radius with several scores indicated by white boxes and corresponding letters. **B)** Magnification of white box B depicting score with also associated ichnotaxon for *Knethichnus parallelum*. **C)** Magnification of white box C depicting parallel scores. D-F) Magnification of associated white boxes from Fig 6A with deep scores for tooth traces and associated ichnotaxon *Linichnus serratus* with serrations especially clear with associated score in Fig 6D.

**Fig 7:**
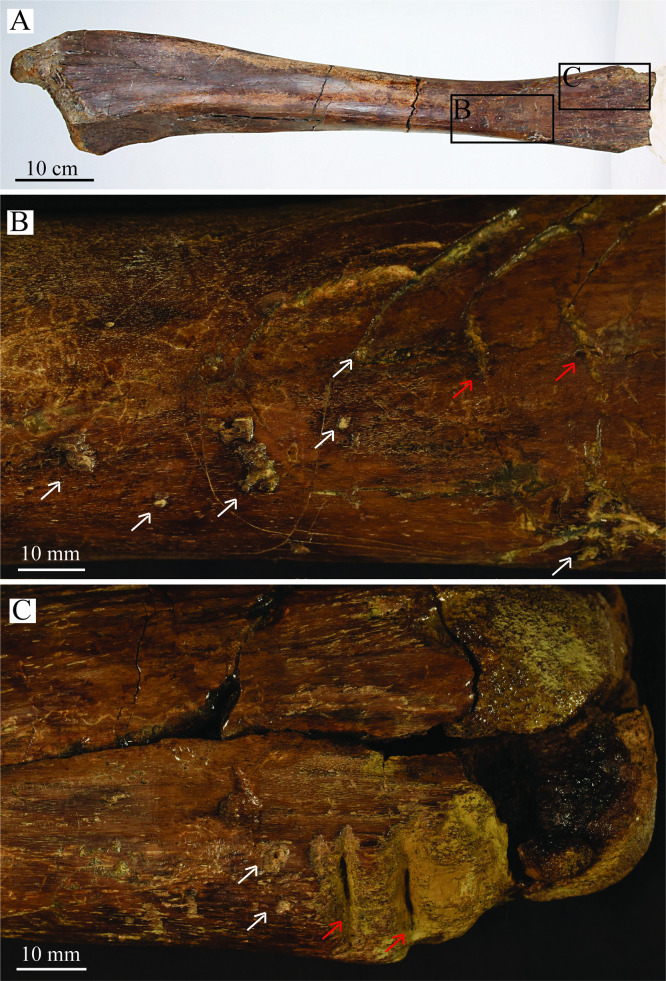
Tooth traces on ulna HRS10076. **A)** Ulna with black boxes indicating areas with tooth traces. **B-C)** Magnification of corresponding boxes from Fig 7A. White arrows indicate pits and red arrows indicate parallel scores.

Additionally, tooth traces are also observed on two different forelimb bones. Specimen HRS13582 (Fig 6), identified as a radius, has six noticeable scores: one score with associated parallel striations indicative of the ichnotaxon *Knethichnus parallelum* (Fig 6B), two parallel linear scores (Fig 6C), and lastly three slightly curved, deep scores with associated serrations indicative of the ichnotaxon *Linichnus serratus* (Fig 6D and 6F). Specimen HRS10076, identified as ulna (Fig 7), also shows tooth traces: eight pits and two sets of two parallel scores located at the distal end (Fig 7B and 7C).

### Tooth trace ichnotaxa

The ichnotaxa *Linichnus serratus* and *Knethichnus parallelum* [29] for score and furrow traces have the potential to be an indicator of the inflictor to the species level. Because *Linichnus serratus* and *Knethichnus parallelum* traces have denticle marks, only carnivores with ziphodont teeth can inflict such traces. The spacing between the parallel lines found in *Knethichnus parallelum* is attributable to the denticles of the inflictor dragging on the surface of a bone during a bite [29]. Thus, specific striation spacing of the trace suggests the identification of specific inflictor’s tooth. A *Linichnus serratus* trace is identified as a curved score with serrated morphology and U- or V-shaped geometry in cross-section. It can be diagnostic to species level when the trace has more than one well-defined and well-preserved serration set, indicative of denticle spacing.

**Fig 8:**
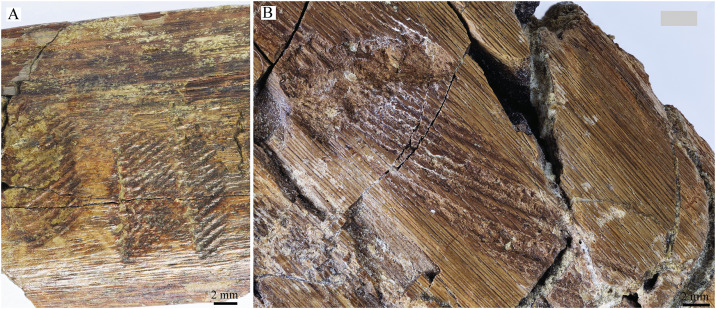
Striation density associated with scores of ichnotaxon *Knethichnus parallelum.* **A)** Scores on rib HRS03387 with striation density of three per 2 mm. **B)** Score on radius HRS13582 with striation density of three per 2 mm.

The ichnotaxon *Knethichnus parallelum* is identified in two different specimens*.* The scores from rib HRS03387 have three parallel *Knethichnus parallelum* traces along the medial surface of the rib (Figs 3F, 3G and 8A). The parallel striations from the three traces on the medial surface of the rib are defined and well-preserved. The density of these striations is three striations per 2 mm (Fig 8A) in all the different traces on the bone. Likewise, one *Knethichnus parallelum* trace is also identified on radius HRS13582 with the parallel striation density of three striations per 2 mm (Figs 6B and 8B). Additionally, three *Linichnus serratus* traces are also identified (Fig 6D–6F). Details of preserved serrations in *Linichnus serratus* traces are essential to determine the denticle density of the inflictor. Although serrations are observed in the three *Linichnus serratus* traces on specimen HRS13582, only one of these traces (Fig 6D) has enough detail to determine the density of two serrations per 2 mm.

*Linichnus serratus* is also identified on two additional fossils in our collection. The caudal vertebra HRS00428 has two curved scores located on the proximal right lateral surface of the neural spine (Fig 5B and 5C). The serration density observed is two per 2 mm. The neural spine fragment HRS03650 also presents two well-preserved examples of *Linichnus serratus* on the opposing sides of the spinal fragment (Fig 4D and 4E). However, the score with the associated traces on the left surface (Fig 4D) is less prominent and deep than the traces associated with the score on the right surface (Fig 4E) indicating serration density of two per 2 mm.

#### Trace maker identification by ichnotaxa.

Because some of the bones in the collection manifested tooth traces that suggested an ichnotaxa, we selected serrated teeth from the same bonebed that could potentially identify probable trace maker. The taxon with ziphodont teeth represented within the bonebed are the tyrannosaurids *Tyrannosaurus rex* and *Nanotyrannus lancensis* (recently indicated as a different taxon [37–39]) the troodontid *Pectinodon bakkeri*, and the dromaeosaurid *Acheroraptor temertyorum*. We present in Table 1 the measurements of denticle density according to taxon from the bonebed (and other comparative bonebeds of the Maastrichtian Lance Formation). Striation densities on *Knethichnus parallelum* traces must be equivalent or less than the denticle density found on the inflictor’s tooth [40]. Denticle density measurement from the teeth of the animals, other than *T. rex*, associated with the bonebed have higher densities, which indicates that the only capable inflictor of the *Knethichnus parallelum* traces was *T. rex.* The density of the serrations from the *Linichnus serratus*, two serrations per 2 mm*,* on the radius HRS13582 (also associated with *Knethichnus parallelum)* confirms traces on the bone to be from *T. rex* bites. Serration density on the neural spines HRS00428 and HRS 03650 is identical to the ones found in the radius HRS13582. Thus, it is parsimonious to suggest that the traces attributed to the two different ichnotaxa are caused by the same species, in this case *T. rex*.

**Table 1:** Tooth measurements for denticle density (per 2 mm) from theropod taxa known associated with examined bonebed of the Lance Formation.

Taxon	*Acheroraptor*	*Nanotyrannus*	*Tyrannosaurus*	*Pectinodon*
AVG MD/2 mm	6.1	5.2	3.2	–
AVG DD/2 mm	6.1	5.1	3.4	6.1
Range MD/2 mm	5.4-6.8	4.1-6.3	2.1-4.3	–
Range DD/2 mm	5.3-6.9	4.1-6.1	2.5-4.3	4.9-7.3
McLain et al. [22]	6.12/5.99	6.1/5.08	3.22/3.79	5.19*
Smith et al. [41]			3.7/3.9	

*Referred to as *Troodon*, DD/2 mm.

Abbreviations: AVG = average; MD/2 mm = mesial denticle density per 2 mm; DD/2 mm = distal denticle density per 2 mm; D/2 mm = denticle density per 2 mm.

## Discussion

Only a small proportion of bones within the studied assemblage (~0.4% from the South and North quarries from material excavated and prepared through the year of 2017) exhibit unambiguous tooth traces. Four specimens preserve diagnostic tooth-trace ichnotaxa: rib HRS03387 (Figs 3F, 3G and 8A), radius HRS13582 (Figs 6 and 8B), caudal vertebra HRS00428 (Fig 5B and 5C), and neural spine fragment HRS03650 (Fig 4D and 4E). In these cases, the spacing and morphology of striations and serrations support attribution to *Tyrannosaurus rex* as the tooth trace maker. Fiorillo [11] also indicated a similar small percentage range of bones with tooth traces and suggested possibly a low predatory behavior by therapods and implications for carcass utilization. However, it is important to mention that some of these bones with tooth traces may also reflect crocodilian activity. Conical, not serrated, lose teeth are also found within the main bonebed of this study and previous reports of crocodile-induced bite marks show similar lesions to the ones found in our analyses [2,25,39,41,42].

Most tooth traces lack associated bone remodeling, suggesting that the animals were inflicted peri- or post-mortem, and indicating that they were subject to predation or, more likely, scavenging. Consistent with this interpretation, previous work on this bonebed [34] documented numerous and consistent rib fractures (smooth surface fracture indicative that the bone was still fresh during lesion) with damage concentrated near the distal ends suggestive of scavenging (in attempt to access the organs). Carcass exposure is also supported by the presence of other bone biogenetic and diagenetic modifications such as bioerosion, and weathering [34]. These findings suggest that remains were exposed prior to burial and subsequently scavenged by theropods, crocodilians, or both, and also may indicate infrequent successful predation.

### Tooth trace criteria

The criteria for attributing a lesion to bite marks is precise but not exhaustive. When bones are associated and/or articulated it is easier to determine whether or not the perforations are lesions representative of tooth traces, due to the overall anatomical context of the animal. However, when examining bones that are disarticulated and mostly dissociated, such as the ones within this study’s bonebed (MNI = 19, left surangular, as in 2017 excavated and prepared bones in the collection), it is crucial to look for clues in each individual bone, within the overall context of the bones, within the bonebed, and at the characteristics of the specific animal species. We have considered the following factors to be important in the analysis of whether bone perforations are tooth traces: a) shape; b) length and width; c) edge; d) depth surface texture; e) depth; f) channeling between perforations when more than one is present; g) location; h) association with bone remodeling; i) general bone morphology and morphology specific to the taxon; j) communality of perforation type with bone association. In some cases, the cause of these perforations attributable to tooth traces is clear, such as in the case of the rib HRS03387 (Fig 3F), neural spine fragment HRS03650 (Fig 4E), and radius HRS13582 (Fig 6) because of the striations and serrations left on the bone by the denticles of the predator’s tooth.

However, in most cases the cause of a lesion is not immediately clear. Therefore, a combination and association of different factors give a contextual understanding that becomes a powerful tool for interpretation on cause or origin. Below, we discuss different combinations and associations of factors that contributed to our interpretation for tooth traces in the bones from this study.

### Contextual factors important for tooth trace criteria

Determining perforation depth and the texture of the perforation edges, and the directly contacted surface (impacted surface) is important because it provides information on natural morphology, bone reaction associated with pathology, or post-mortem erosion. Perforation with a smooth surface usually suggests fresh/malleable or responsive bone. On specimen HRS09477 (Fig 3C) and HRS01295 (Fig 4B), the location and appearance of the perforations are atypical for this type of bone. The smoothness of the texture along the edges of the perforation and impacted surface suggests fresh/malleable bone or early stages of bone remodeling. In both specimens, the pull and dragging along the edges of perforations, typically representing tooth traces, is also observed. Thus, we concluded that these perforations are tooth traces from premortem bites with sometime for early stages of bone remodeling followed by death, or most likely perimortem bites.

However, perforations with smooth edges also may indicate a normal osteological feature such as foramina. If one is not familiar with the specific osteological features in a specimen, especially in extinct species, these perforations could potentially be mistakenly identified as a tooth trace, especially for the untrained eye. The surangular HRS05499 (Fig 1) is a great example of the need for further investigation of such perforations. The perforations shape and arrangement initially could be mistakenly identified as tooth trace lesions caused by a crocodile bite. However, the context of the “bite” combined with the texture of the perforations and CT scan results, indicated that the perforations were not tooth traces but a natural bony feature for the surangular, most likely foramina, not identified into any of the other surangulars in the *E. annectens* bonebed. This is supported by previous findings that the surangulars of hadrosaurs do not have foramina [43]. Although the examined bonebed is dominated by *E. annectens*, our study of the surangular specimen HRS 05499 prompted further investigation of surangulars from other contemporaneous taxa. The main bonebed also contains occasional triceratopsine dinosaur elements. Consequently, we surveyed additional museum collections to compare ceratopsian surangulars with HRS 05499 and identified a larger but closely matching specimen, BHI 4772 (S3 Fig), housed at the Black Hills Institute of Geological Research. This specimen belongs to a sub-adult *Torosaurus latus*. Thus, we determined that the details in the texture, depth and context for perforations are important for etiology. We also suggest that for future study, the examination of more surangular bones from ceratopsids is necessary because the presence of foramina only in surangulars from Triceratopsini is potentially diagnostic for the particular tribe or even for the specific genus *Torosaurus*.

Perforations with smooth edges can also suggest channel formation as the bone responds to infection, which is often associated with complications from trauma. In mammal bones, infection is indicated by the presence of noticeable openings from channels called cloacae necessary for pus drainage during the healing process [44,45]. The process is different in reptiles and birds, where a casseating granuloma (a more solid substance) forms at the infection site [46–48] A more mammal-like response to infection has been suggested to occur in dinosaurs [33,35,49,50]. The organization and characteristics of the lesions observed in the previously reported neural spine HRS07948 [35], including its association with the evident misalignment and bone proliferation, indicates fracture of the neural spine with an infectious response. Thus, we conclude that it is important to consider the context and morphological details of perforations.

Joint related pathologies can also leave a perforation or depression on the articular surface of bones. In such cases, we observe lesions to also have smooth edges and bone response in common joint surfaces, often associated with sclerotic bone. Previously reported analysis of bones from this study’s bonebed illustrates caudal vertebrae HRS07804, HRS07887, HRS01254 and HRS05808 to have perforations in the articular surface of the centrum [35]. When considering only the descriptive criteria for tooth traces in fossils (S1 Fig), these specimens could be mistakenly identified as a tooth trace. However, the context of the lesions makes these perforations questionable candidates for a tooth trace. The smooth edges, along with the location of the perforation (on the lateral aspect of the joint surface of the centrum), suggests an unlikely place for a bite. CT scans showed bone reaction associated with lesions [35] thus suggesting pathology. These types of lesions may be characteristic of mechanical stress due to frequent tail injury affecting the joint surface of caudal vertebral centra. Rothschild and Tanke [51] have previously reported other joint diseases in hadrosaur bones and suggested genetic predisposition.

In this study, we also report several bones with relatively unambiguous bite marks. These types of lesions, when associated with extending striations or serrations, are recognized as ichnotaxa. The ichnotaxa in this study, *Knethichnus paralellum* (Fig 8) and *Linichnus serratus* (Figs 4E, 5C and 6D), have striation and serration density of two per 2 mm. Although we have measured all the possible ziphodont teeth from animals associated with the bonebed, the only animal with the denticle density average (or range of density) compatible with the striation or serration density is *T. rex*. The other taxa had more denticles per mm and their denticle density range was always higher than four denticles per 2 mm. The *Knethichnus paralellum* and *Linichnus serratus* traces in this study did not present signs of bone remodeling associated with the traces, suggesting that the bites occurred peri-mortem or post-mortem. While we cannot be conclusive on the identification of the inflictors associated with all the tooth traces in this study, we have evidence that *T. rex* bit the bones of some of these animals. On the radius HRS13582, the spacing of the very definite linear parallel scores (10 mm apart) is characteristic of *Knethichnus paralellum* and *Linichnus serratus* traces (Fig 6C). These scores might also indicate the interdental spacing of the inflictor, in this case *T. rex*. Similarly, the ulna HRS10076 has also definite linear and parallel scores with similar interdental spacing of 10 mm (Fig 7B and 7C). However, the ulna scores do not have associated ichnotaxa traces diagnostic for the nature of the inflictor. Nonetheless, it is reasonable to identify (by comparison with the radius HRS13582) *T. rex* as the inflictor of the bite in the ulna. It is important to note that many of these predators shed and replaced their teeth, and the angle of bite varies. Therefore, we can only be precise on the identification of the bite inflictor when the parallel scores are in association with tooth trace ichnotaxa.

There are other caveats to consider in tooth trace identification. First, any kind of trace in cancellous bone is always questionable. Without the cortical surface present, it is very difficult to have any idea of what happened to cause the perforation or groove on cancellous bone. In this study, we did not find any lesions affecting cancellous bone in association with cortical bone. Thus, we may be underestimating the frequency of bite marks from this bonebed due to the difficulty of identification. Unless a tooth is found lodged in the trace [5,52], all traces in cancellous bone are ambiguous. There are extreme cases of traces in cancellous bone associated with shearing. In bone shearing there is a complete removal of a bone piece from a bite, thus making it challenging to preserve any trace.

However, there may be possible cases of shearing where signs of a bite can be concluded. Carpenter [13] reported a hadrosaur caudal neural spine missing its distal end. The neural spine also has associated bone proliferation. Other caudal neural spines in connected vertebrae had tooth traces. Thus, the author concluded that the missing and injured neural spine resulted from a bite taken out of the animal during his life and bone proliferation is most likely a response to an infection acquired from the bite [13]. Even though no tooth or tooth traces were associated with the neural spine, it is tempting to hypothesize this case to be an example of “bite trace” with no direct evidence. This example may also indicate a case of pseudarthrosis which resulted from a failed fracture union [33].

In our study, the neural spine HRS00428 (Fig 5A) represents a similar specimen as previously described by Carpenter [13]. The bone is also missing its distal end with bone proliferation. A fracture, not resulting from a bite, is unlikely since only a small segment of the distal neural spine is missing. In the same specimen, we also have unhealed tooth traces (Fig 5C) on the proximal segment of the bone. Therefore, we conclude that this specimen represents unrelated examples of lesions caused by bites. While the lesion associated with the distal neural spine occurred while the animal was alive and with time to heal, the bite associated with the scores occurred either peri-mortem or post-mortem.

Another caveat to consider in tooth trace identification pertains to traces found in highly fractured bones. Tooth traces can be camouflaged or destroyed within fractured regions that can often occur in scavenging or predation. It is important to note that bone fractures during trampling, transport or post-burial compaction fractures can directly impact the preservation of tooth traces since bitten areas might be structurally weaker.

An additional layer of complexity when considering the identification for a perforation is the similarity between pit- or puncture-like tooth traces and other perforations caused by bioerosion. It is important to be familiar with the different types of ichnotaxa for bone bioerosion [53]. Although most cases of bioerosion in bones are usually unambiguous, the more pit/puncture like perforations might be mistaken for a tooth trace. Such cases are bivalve macroborings on bones [54,55], and chambers created by necrophagous larvae [34,53,56–58] that might have the shape of a tooth trace (puncture or pit). Their shape might be very similar to a puncture or a pit caused by a tooth, especially in isolated instances. The gross appearance of the edges of these postmortem bioerosive perforations also might appear smooth and resemble a tooth trace made while the bone was still fresh. Thus, histological examination of a perforation and its associated context is important to determine etiology.

Finally, when evaluating different types of bone perforations as potential tooth traces, it is essential to distinguish them from other bone surface modifications, such as trampling damage and human-generated marks in the fossil or archaeological record [59–63]. Although this remains a subject of ongoing debate, patterning has been identified as a key criterion for differentiation. Trampling typically produces multiple, parallel, shallow striations with oblique or transverse orientations across the bone surface, whereas human-generated marks tend to occur in specific locations and often appear as isolated, deep grooves indicative of deliberate behavior [59]. However, tooth traces generally present wider grooves than those produced by human activities such as butchery [62]. When exploring methods to differentiate fossil excavation tools and preparation tools [64] on dinosaur bone surfaces using commonly used tools, it was determined that tool marks on bone surface could be distinguished from authentic tooth traces [64]. Tool marks often left shallower depressions, even when more force was applied by the examiner. Also, the shape of the depressions created by tools usually were of uneven patterns and with flaking of the edges [64].

**Fig 9:**
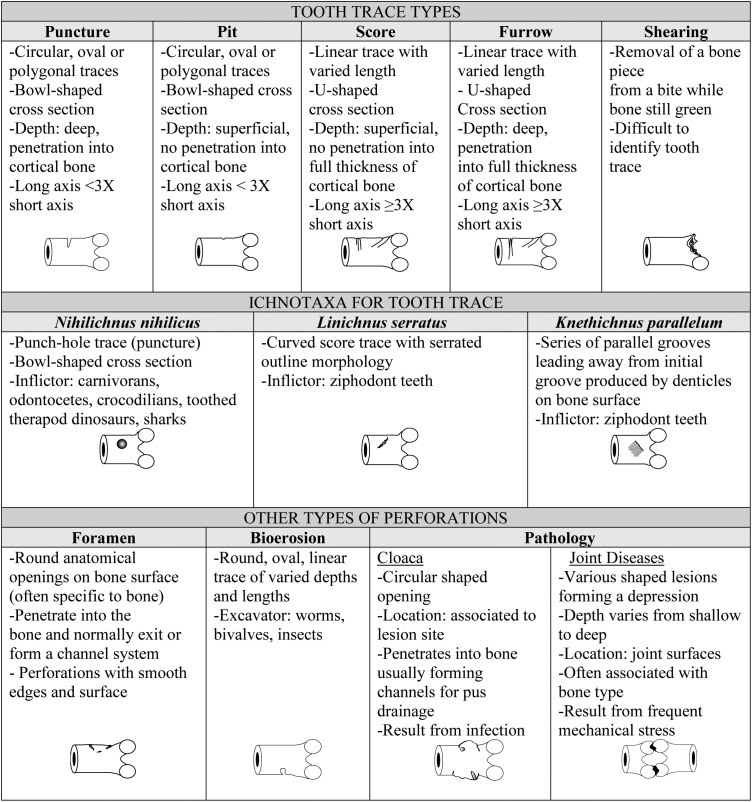
Types of bone perforation with contextual application.

As a result of this study, we adapted the guidelines for the identification of tooth traces (Fig 9) to incorporate many different types of bone perforations. Such perforations can result from natural bone morphology such as foramina in addition to natural bone response to infection or frequency of mechanical stress on joint surfaces. We have also incorporated previous work on tooth trace types and identification (S1 Fig) including ichnotaxa for tooth traces. Although shearing of bone is not a type of bone perforation, its context can be considered as evidence for a bite. Thus, we also integrated shearing into our guideline. However, it is important to note that shearing is often difficult to interpret and quantify due to the lack of direct evidence. Finally, we also consider post-mortem perforations such as bioerosive process, especially macroboring due to the larger size and shape that might potentially be mistaken for another type of bone perforation. This new guide (Fig 9) for the different types of bone perforations thus refines the criteria for identification of tooth traces and will be helpful for correctly labeling bone perforations.

## Conclusion

In this study we report only a very small fraction of the bonebed assemblage (~0.4%) preserved clear tooth traces, with a few specimens confidently attributable to distinct ichnotaxa. The morphology and spacing of these ichnotaxa for tooth traces are most consistent with feeding activity by *Tyrannosaurus rex*. However, some traces may also reflect crocodilian involvement. The general absence of bone remodeling indicates that most traces were inflicted peri- or postmortem. This interpretation is further supported by associated fracture patterns in ribs, bioerosion, and weathering, all of which indicate that carcasses remained exposed prior to burial. Overall, the evidence suggests limited predatory behavior and carcass utilization likely dominated by opportunistic scavenging by theropods, crocodilians, or both.

The correct identification of the different types of bone perforations is necessary especially when examining for tooth traces. Herein, we identified and classified different types of tooth traces from an *Edmontosaurus annectens* bonebed within the Lance Formation, WY. We described several types of traces and associated features, such as evidence for bone remodeling, different types of tooth trace ichnotaxa useful for inflictor identification and shearing when there is no direct evidence of a tooth trace. We also identified and determined the nature of other types of bone perforations that potentially could be mistaken for tooth traces such as foramina, cloaca, cartilaginous inclusions/bone necrosis, and bioerosion. Consequently, we developed a guide for the identification of bone perforations and associations to tooth trace criteria. Further investigations of more specimens from this bonebed will continue, and we expect more examples of tooth traces and other types of bone perforations that will further refine the guide (Fig 9).

We also invite other researchers to collaborate on interpretations and identifications of the different types of bone perforations. Refining the criteria for tooth traces in the context of bone perforation is important and will help interpretations of animal biting behavior as well as our understanding of dinosaur paleopathology, natural bone morphology and biomechanics.

## Supporting information

S1 Fig Criteria for tooth traces. Compilation of literature review of tooth trace classification (modified after Binford 1981; Mikuláš et al 2006; Njau and Blumenschine 2006; Pobiner et al 2007; Pobiner 2008; Jacobsen and Bromley 2009).
(DOCX)

S2 Fig Tooth denticle density measurements. *Tyrannosaurus rex* tooth HRS06541 is used for illustration purpose.
(TIF)

S3 Fig Surangular of a sub-adult *Torosaurus latus* BHI 4772. Numbers indicate individual foramen according to Fig 1.
(TIF)
