# Supplementary material for: Identification of tooth traces from a Cretaceous (Maastrichtian) Edmontosaurus annectens bonebed in the Lance Formation, Wyoming, U.S.A
Source: PLoS One. 2026 Jul 15;21(7):e0351939. doi: 10.1371/journal.pone.0351939 (PMC13372169; doi:10.1371/journal.pone.0351939)
Supplement: S1 Fig — Compilation of literature review of tooth trace classification (modified after Binford 1981; Mikuláš et al 2006; Njau and Blumenschine 2006; Pobiner et al 2007; Pobiner 2008; Jacobsen and Bromley 2009). (DOCX) [file pone.0351939.s001.docx]

| PUNCTURE | PIT | | SCORE | |
| --- | --- | --- | --- | --- |
| -Shape: circular, oval or polygonal traces  -Bowl-shaped cross- sections  -Trace Bite: deep, penetration into cortical bone  -Long axis <3X short axis | -Shape: circular, oval or polygonal traces  -Bowl-shaped cross-sections  -Trace Bite: superficial, no penetration into cortical bone  -Long axis < 3X short axis | | -Shape: linear trace with  varied length  -Cross-section: U-shaped  -Trace Bite: superficial with  no penetration into full thickness  of cortical bone  -Long axis ≥3X short axis | |
|  |  | |  | |
| FURROW | ICHNOTAXA | | | |
| -Shape: linear trace with varied length  -Cross-section: U-shaped  -Trace Bite: deep, penetration  into full thickness of cortical bone  -Long axis ≥3X short axis | *Nihilichnus nihilicus*  -Shape: punch-hole puncture trace  -Inflictor: carnivorans, odontocetes, crocodilians, toothed therapod dinosaurs, sharks | *Linichnus serratus*  -Shape: curved score with serrations  -Cross-section: U or V  -Inflictor: carnivore with ziphodont teeth | | *Knethichnus parallelum*  -Series of parallel grooves leading away from initial groove  -Produced by: denticles on bone surface  -Inflictor: carnivore with ziphodont teeth |
|  |  |  | |  |
