# Supplementary figures and images for: Identification of tooth traces from a Cretaceous (Maastrichtian) Edmontosaurus annectens bonebed in the Lance Formation, Wyoming, U.S.A
Source: PLoS One. 2026 Jul 15;21(7):e0351939. doi: 10.1371/journal.pone.0351939 (PMC13372169; doi:10.1371/journal.pone.0351939)

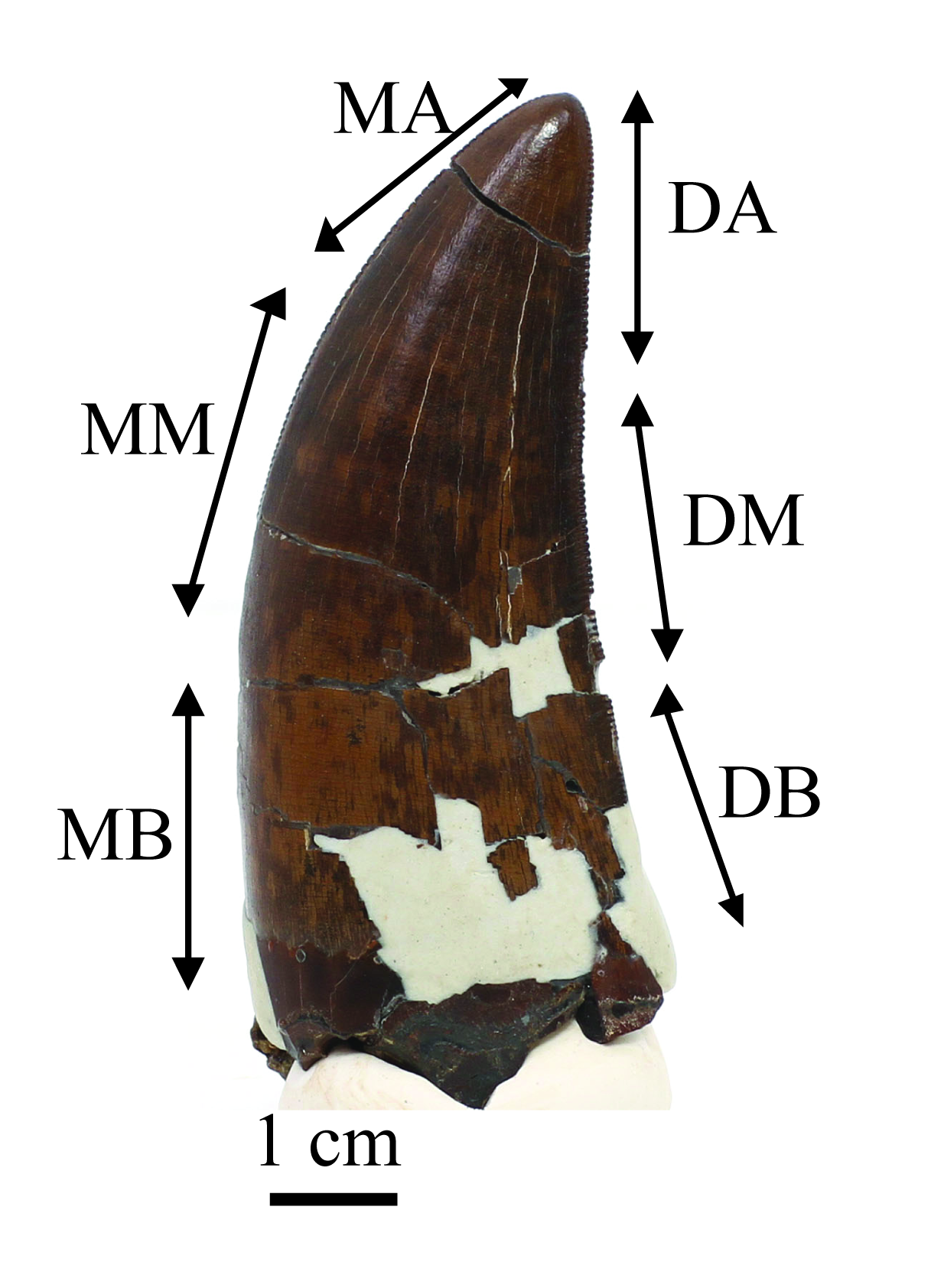

Supplement: S2 Fig — Tyrannosaurus rex tooth HRS06541 is used for illustration purpose. (TIF) [file pone.0351939.s002.tif]

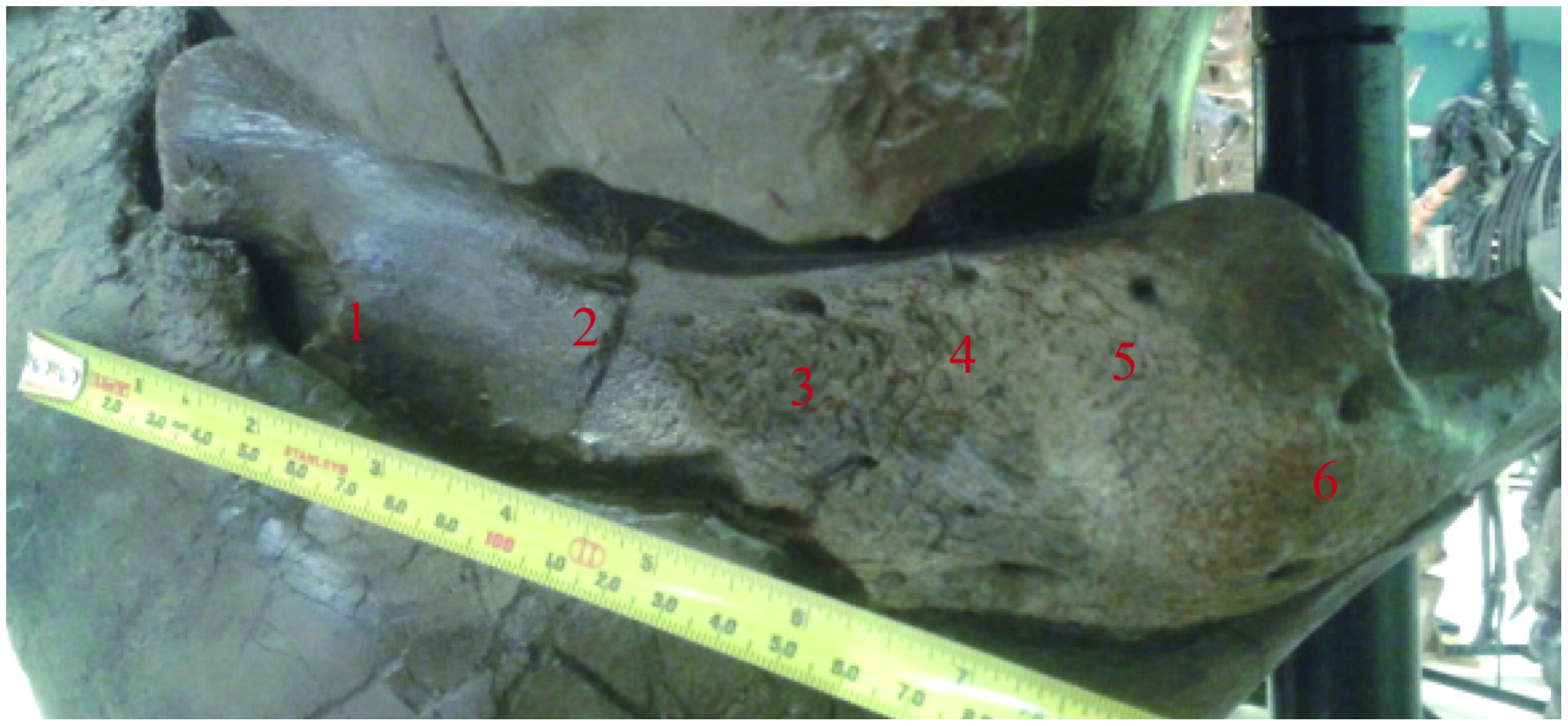

Supplement: S3 Fig — Numbers indicate individual foramen according to Fig 1. (TIF) [file pone.0351939.s003.tif]
