# Supplementary material for: Identification of tooth traces from a Cretaceous (Maastrichtian) Edmontosaurus annectens bonebed in the Lance Formation, Wyoming, U.S.A
Source: PLoS One. 2026 Jul 15;21(7):e0351939. doi: 10.1371/journal.pone.0351939 (PMC13372169; doi:10.1371/journal.pone.0351939)
Supplement: Attachment — Submitted filename: PONE-D-25-67537_reviewer.pdf [file pone.0351939.s005.pdf]

PLOS One

Identification of tooth traces from a Cretaceous (Maastrichtian) Edmontosaurus annectens bonebed in the Lance Formation, Wyoming, U.S.A.

--Manuscript Draft--

**Manuscript Number:**	PONE-D-25-67537
**Article Type:**	Research Article
**Full Title:**	Identification of tooth traces from a Cretaceous (Maastrichtian) Edmontosaurus annectens bonebed in the Lance Formation, Wyoming, U.S.A.
**Short Title:**	Identification of tooth traces from a Cretaceous E. annectens bonebed in the Lance Formation, Wyoming, U.S.A.
**Corresponding Author:**	Bethania C. T. Siviero, PhD<br>Loma Linda University School of Medicine<br>Loma Linda, CA UNITED STATES OF AMERICA
**Keywords:**	Paleontology; Tooth trace; taphonomy; paleopathology; Edmontosaurus annectens; Lance formation; Cretaceous; bonebed; vertebrate paleontology; bone; fossil; dinosaur; hadrosaur; triceratops, t. rex; nanotyrannus; tyrannosaurus
**Abstract:**	Identifying the origin of perforating lesions on fossil bone is often difficult, and many are considered tooth traces, in spite of more likely and more parsimonious etiologies. Much of this confusion stems from tooth trace criteria that are ambiguous when the context for the lesions is not considered. Mistaken identification of tooth traces has led to misleading interpretations of animal behavior. This study of tooth traces on fossil bones critically reviews previous criteria and applies them to assessing bones from an Edmontosaurus annectens bonebed within the Lance Formation, Wyoming, USA. Of the 3013 bones examined, thirteen bones had features indicative of tooth traces based on gross appearance. Of these, one bone had perforations determined to have a different etiology. Twelve bones had traces attributed to tooth marks, including four bones with Knethichnus parallelum and Linichnus serratus ichnotaxa. Tyrannosaurus rex was identified as the likely inflictor of traces attributable to both ichnotaxa, by comparison of denticle density of carnivore teeth within the bonebed with striation/serration density of the traces. The importance of context in the analysis of perforating lesions on fossil bones is shown through the mistaken identification of features such as neurovasculature foramina and lesions associated with pathology as tooth traces. The application of refined criteria was useful in correctly labeling bone tooth traces, which adds to the literature on animal biting behavior in fossil taxa and stimulates further research on this topic.
**Order of Authors:**	Bethania C. T. Siviero, MS; PhD
	Elizabeh Rega, PhD
	Matthew A. McLain, PhD
	Leonard R. Brand
	David Nelsen, PhD
	Art V. Chadwick, PhD
**Additional Information:**	
**Question**	**Response**
**Financial Disclosure**<br><br>Enter a financial disclosure statement that describes the sources of funding for the work included in this submission. Review the submission guidelines for detailed requirements. View published research	Yes

Powered by Editorial Manager® and ProduXion Manager® from Aries Systems Corporation

articles from *PLOS ONE* for specific examples.<br><br>This statement is required for submission and **will appear in the published article** if the submission is accepted. Please make sure it is accurate.<br><br>**Funded studies**<br>Enter a statement with the following details:<br>• Initials of the authors who received each award<br>• Grant numbers awarded to each author<br>• The full name of each funder<br>• URL of each funder website<br>• Did the sponsors or funders play any role in the study design, data collection and analysis, decision to publish, or preparation of the manuscript?<br><br>Did you receive funding for this work?	
Please add funding details.<br>as follow-up to "**Financial Disclosure**<br><br>Enter a financial disclosure statement that describes the sources of funding for the work included in this submission. Review the submission guidelines for detailed requirements. View published research articles from *PLOS ONE* for specific examples.<br><br>This statement is required for submission and **will appear in the published article** if the submission is accepted. Please make sure it is accurate.	Internal fundings from Loma Linda University

**Funded studies**<br>Enter a statement with the following details:<br>• Initials of the authors who received each award<br>• Grant numbers awarded to each author<br>• The full name of each funder<br>• URL of each funder website<br>• Did the sponsors or funders play any role in the study design, data collection and analysis, decision to publish, or preparation of the manuscript?<br><br>Did you receive funding for this work?"	
Please select the country of your main research funder (please select carefully as in some cases this is used in fee calculation).<br>as follow-up to "**Financial Disclosure**<br><br>Enter a financial disclosure statement that describes the sources of funding for the work included in this submission. Review the submission guidelines for detailed requirements. View published research articles from *PLOS ONE* for specific examples.<br><br>This statement is required for submission and **will appear in the published article** if the submission is accepted. Please make sure it is accurate.<br><br>**Funded studies**<br>Enter a statement with the following details:<br>• Initials of the authors who received each award<br>• Grant numbers awarded to each author<br>• The full name of each funder<br>• URL of each funder website<br>• Did the sponsors or funders play any role in the study design, data collection and analysis, decision to publish, or preparation of the manuscript?<br><br>Did you receive funding for this work?"	UNITED STATES - US
**Competing Interests**	The authors have declared that no competing interests exist.

Use the instructions below to enter a competing interest statement for this submission. On behalf of all authors, disclose any competing interests that could be perceived to bias this work—acknowledging all financial support and any other relevant financial or non-financial competing interests.<br><br>This statement is required for submission and **will appear in the published article** if the submission is accepted. Please make sure it is accurate and that any funding sources listed in your Funding Information later in the submission form are also declared in your Financial Disclosure statement.<br><br>View published research articles from *PLOS ONE* for specific examples.<br><br>**NO authors have competing interests**<br><br>Enter: *The authors have declared that no competing interests exist.*<br><br>**Authors with competing interests**<br><br>Enter competing interest details beginning with this statement:<br><br>*I have read the journal's policy and the authors of this manuscript have the following competing interests: [insert competing interests here]*<br><br>* typeset	
**Ethics Statement**<br><br>Enter an ethics statement for this submission. This statement is required if the study involved:<br><br>• Human participants<br>• Human specimens or tissue<br>• Vertebrate animals or cephalopods<br>• Vertebrate embryos or tissues	N/A

• Field research<br><br>Write "N/A" if the submission does not require an ethics statement.<br><br>General guidance is provided below. Consult the submission guidelines for detailed instructions. **Make sure that all information entered here is included in the Methods section of the manuscript.**	

Powered by Editorial Manager® and ProduXion Manager® from Aries Systems Corporation

**Format for specific study types**<br><br>**Human Subject Research (involving human participants and/or tissue)**<br>• Give the name of the institutional review board or ethics committee that approved the study<br>• Include the approval number and/or a statement indicating approval of this research<br>• Indicate the form of consent obtained (written/oral) or the reason that consent was not obtained (e.g. the data were analyzed anonymously)<br><br>**Animal Research (involving vertebrate animals, embryos or tissues)**<br>• Provide the name of the Institutional Animal Care and Use Committee (IACUC) or other relevant ethics board that reviewed the study protocol, and indicate whether they approved this research or granted a formal waiver of ethical approval<br>• Include an approval number if one was obtained<br>• If the study involved *non-human primates*, add *additional details* about animal welfare and steps taken to ameliorate suffering<br>• If anesthesia, euthanasia, or any kind of animal sacrifice is part of the study, include briefly which substances and/or methods were applied<br><br>**Field Research**<br><br>Include the following details if this study involves the collection of plant, animal, or other materials from a natural setting:<br>• Field permit number<br>• Name of the institution or relevant body that granted permission	
**Data Availability**<br><br>Authors are required to make all data underlying the findings described fully available, without restriction, and from the time of publication. PLOS allows rare exceptions to address legal and ethical concerns. See the PLOS Data Policy and FAQ for detailed information.	Yes - all data are fully available without restriction

A Data Availability Statement describing where the data can be found is required at submission. Your answers to this question constitute the Data Availability Statement and **will be published in the article**, if accepted.<br><br>**Important:** Stating 'data available on request from the author' is not sufficient. If your data are only available upon request, select 'No' for the first question and explain your exceptional situation in the text box.<br><br>Do the authors confirm that all data underlying the findings described in their manuscript are fully available without restriction?	
**Describe where the data may be found in full sentences. If you are copying our sample text, replace any instances of XXX with the appropriate details.**<br><br>• If the data are **held or will be held in a public repository**, include URLs, accession numbers or DOIs. If this information will only be available after acceptance, indicate this by ticking the box below. For example: *All XXX files are available from the XXX database (accession number(s) XXX, XXX.*).<br>• If the data are all contained **within the manuscript and/or Supporting Information files**, enter the following: *All relevant data are within the manuscript and its Supporting Information files.*<br>• If neither of these applies but you are able to provide **details of access elsewhere**, with or without limitations, please do so. For example:<br><br>*Data cannot be shared publicly because of [XXX]. Data are available from the XXX Institutional Data Access / Ethics Committee (contact via XXX) for researchers who meet the criteria for access to confidential data.*<br><br>*The data underlying the results presented in the study are available from (include the name of the third party*	Images of the fossils can be found at https://fossil.swau.edu

and contact information or URL).<br>• This text is appropriate if the data are owned by a third party and authors do not have permission to share the data.<br><br>* typeset	
Additional data availability information:	

Powered by Editorial Manager® and ProduXion Manager® from Aries Systems Corporation

Manuscript
Click here to access/download;Manuscript;E annectens tooth traces Manuscript.docx

Identification of tooth traces from a Cretaceous (Maastrichtian) *Edmontosaurus annectens* bonebed in the Lance Formation, Wyoming, U.S.A.

Bethania C. T. Siviero [1, 4*], Elizabeth Rega [2], Matthew A. McLain [3,4], Leonard R. Brand [4], David Nelsen [5], Art V. Chadwick [6]

[1] Department of Pathology and Human Anatomy, Loma Linda University, Loma Linda, California, United States of America

[2] Department of Medical Anatomical Sciences, Western University of Health Sciences, Pomona, California, United States of America

[3] Department of Biological and Physical Sciences, The Master's University, Santa Clarita, California, United States of America

[4] Department of Earth and Biological Sciences, Loma Linda University, Loma Linda, California, United States of America

[5] Department of Biology, Southern Adventist University, Chattanooga, Tennessee, United States of America

[6] Department of Biology, Southwestern Adventist University, Keene, Texas, United States of America

[*] Corresponding author

E-mail: bsiviero@llu.edu

Abstract

Identifying the origin of perforating lesions on fossil bone is often difficult, and many are considered tooth traces, in spite of more likely and more parsimonious etiologies. Much of this confusion stems from tooth trace criteria that are ambiguous when the context for the lesions is not considered. Mistaken identification of tooth traces has led to misleading interpretations of animal behavior. This study of tooth traces on fossil bones critically reviews previous criteria and applies them to assessing bones from an *Edmontosaurus annectens* bonebed within the Lance Formation, Wyoming, USA. Of the 3013 bones examined, thirteen bones had features indicative of tooth traces based on gross appearance. Of these, one bone had perforations determined to have a different etiology. Twelve bones had traces attributed to tooth marks, including four bones with *Knethichnus parallelum* and *Linichnus serratus* ichnotaxa. *Tyrannosaurus rex* was identified as the likely inflictor of traces attributable to both ichnotaxa, by comparison of denticle density of carnivore teeth within the bonebed with striation/serration density of the traces. The importance of context in the analysis of perforating lesions on fossil bones is shown through the mistaken identification of features such as neurovasculature foramina and lesions associated with pathology as tooth traces. The application of refined criteria was useful in correctly labeling bone tooth traces, which adds to the literature on animal biting behavior in fossil taxa and stimulates further research on this topic.

Introduction

Reports of dinosaur bones with tooth traces often attract widespread attention, because of their association with animal behavior. Previous reports on the occurrence and frequency of

cranial bite marks have suggested intraspecific behavior [1] possibly indicating intersexual display [2]. Cannibalistic behavior amongst dinosaurs has also been reported [3, 4] including cases of *Tyrannosaurus rex* cannibalism [5-7] and other theropods [3]. Rarely is the direct evidence of the inflictor in the actual tooth trace reported. However, Currie and Jacobsen [8] described an occasion of a bite mark with an embedded tooth from a velociraptorine theropod on an azhdarchid pterosaur tibia. The authors suggested theropod scavenging on a pterosaur carcass because of the lack of bony healing, although the authors did not consider the possibility of wounds implicated perimortem.

The occurrence of tooth traces in the fossil record has also prompted discussions of interspecific behavior. Noto et al. [9] examined limb bones from ornithopods with markings attributed to tooth traces. The morphology and arrangement of marks were comparable with the dental remains of an associated large crocodyliform, which was suggested to have been the inflictor. Lei et al. [10] also reported the widespread occurrence of tooth traces associated with theropods on sauropod fossil bones, suggesting the preferential feeding of sauropod carcasses by theropods.

Over the years, there have been several attempts to determine evidence-based criteria for bones with tooth traces [11-14]. The criteria are based on description and classification of bone perforations interpreted as bite marks or tooth traces. The consensus is that there are four basic types of tooth traces: 1) pit; 2) puncture; 3) score; and 4) furrow. These were first identified and described by Binford [11] and while these four types are still in use today, their definitions have changed with the passage of time. Although ~~most these~~ definitions came from the observations of mammalian carnivores using either canines or carnassials to mark/perforate bone; these definitions for tooth traces can also be used with other carnivorous animals such as dinosaurs,

crocodilians and sharks, due to similar functional characteristics of flesh-tearing or bone-crushing teeth and underlying similarities of bone structures.

Tooth traces have in their own right been described and classified as ichnotaxa. Ichnotoxa are preserved remains of ancient behavior. Typically, ichnotaxa consist of footprints and less frequently, scratch marks, burrows, and bite marks. Mikuláš, et al. [15] designated punch-hole, puncture traces as an ichnotaxon named *Nihilichnus nihilicus*. Jacobsen and Bromley [16] named two tooth trace ichnotaxa based on dinosaur tooth trace specimens: *Linichnus serratus* and *Knethichnus parallelum*. *Linichnus serratus* is a curved score with a U- or V-shaped geometry in cross-section and a serrated morphology. *Knethichnus parallelum* consists of a series of parallel grooves leading in some cases away from an initial groove, caused by the denticles of a ziphodont tooth dragging along the bone surface. Carnivorans (mammalian carnivores of the order Carnivora), odontocetes, crocodilians, toothed theropod dinosaurs, sharks, and other organisms are capable of producing *Nihilichnus nihilicus*, but only carnivores with ziphodont teeth, such as theropods, varanids, and sharks are capable of producing *Knethichnus parallelum* and *Linichnus serratus*. Thus, there is not a one-to-one relationship of the ichnotaxa with the organism causing the trace. A single ichnotaxon can be paired with multiple perpetrators.

The general criteria for tooth traces and bite marks can also describe processes not due to bite damage. Not everything that looks like a pit, puncture, score, or furrow is necessarily a tooth trace (see S1 Table for a review of the criteria). An example is the bone perforations on the left surangular from the *Tyrannosaurus rex* FMNH PR 2081 also known as "Sue." Although some reports suggested that the lesions were associated with healing from bone penetrating trauma [17], other reports proposed these lesions to be the result of a biogenic bone infection that affected the mandibular region of this animal [18-20]. Wolf et al. [19] described the perforations

as multiple round erosive lesions with smooth-edges and associated bone remodeling. Similar features were also reported as occurring on the mandibles of other tyrannosaurid and archosaur specimens [19, 20]. A conclusive analysis of the lesions' shape and arrangement as well as their contextual location and frequency in other similar animals suggested a bacterial or protozoan infection that affected tyrannosaurids as a result of intraspecific behavior with other infested animals [19].

Thus, even though many bone lesions appear to be tooth traces, it is important to carefully consider their association with bone context. Some examples of bone lytic lesions that can be mistaken for tooth traces are pitting caused by taphonomic erosion, natural bone morphology (such as neural and vascular foramina), some types of joint diseases, and erosive infections. It is also important to note that advanced bone healing from bite injury can camouflage the presence of a tooth trace.

Extraordinary claims of tooth traces on a bone should be subject to rigorous examination because, when misidentified and exaggerated, they have the potential to lead to erroneous conclusions pertaining to intra- or interspecific animal behavior [19]. In this study, we examined the fossil bones for perforations indicative of tooth traces from a mostly monospecific *Edmontosaurus annectens* bonebed, within the Maastrichtian Lance Formation in eastern Wyoming. This bonebed has been previously described as normally graded with mostly disarticulated and disassociated well-preserved bones [21]. The examined bones for the perforations were rigorously compared with previously published identification and classification for tooth traces, including ichnotaxa associated with different types of perforations that are tooth trace-like. Context for these perforations were also examined to determine if it was a legitimate tooth trace or an ambiguous perforation of different etiology. Consequently, we

revise and refine criteria for tooth traces and also expand and discuss other types of bone perforations that have the potential to be mistaken for tooth traces.

Materials and Methods

We examined approximately 3013 dinosaur bones (mostly *E. annectens*) initially found and excavated from a bonebed near the Hanson Ranch Station (HRS) in Roxson, northeastern Wyoming between 1997-2017. Bones were prepared and stored at the Dinosaur Research Center located at Southwestern Adventist University (SWAU) in Keene, TX. Fossils from the two largest quarries, North and South quarries (Fig 1), in the same bonebed were subject to screening by the first author. Marks or perforations that potentially indicated tooth traces were further examined with a magnifier loupe (Bausch & Lomb 10X), Dino-Lite Edge digital microscope, and stereo microscope. Using the previously reported definitions and descriptions for tooth traces (S1 Fig), we identified and classified the bones with potential tooth traces. Pictures of specimens were taken with a Canon EOS D30 camera on a Beseler CS-21 copy stand cameral platform with external lighting for better image contrast. For images that required thin/narrow focal plane and extended depth of field at high resolution, we used photo stacking Helicon Software and Adobe Photoshop Lightroom CC. Specimens with possible tooth traces that were ambiguous were further investigated with a CT scan taken at the Loma Linda Medical Center.

Additional images of specimens reported in this paper are available for viewing in the SWAU fossil catalog available at https://fossil.swau.edu.

Fig 1. Map of the bonebed site and quarries included in this study.

Some of the bones manifested markings attributable to *Knethichnus parallelum* and *Linichnus serratus* ichnotaxa [16]. In order to attempt identification of the probable predator associated with the tooth traces, we selected the bones with *Knethichnus parallelum* traces and measured the spacing of the parallel striations to determine denticle density (number of denticles per 2 mm). We used a 10 mm microruler (TDI International, Inc.) under the stereo microscope (Nikon SMZ-10A) for this measurement. We selected different ziphodont teeth from the associated bonebed to compare denticle densities to the striation density from the *Knethichnus parallelum* traces. We measured the number of denticles per 2 mm under the Dino-Lite microscope. Forty different teeth (ten specimens per selected species) were measured for mesial and distal denticular density (S2 Fig) and give the density average for each species.

Results

Out of the 3013 specimens examined from the largely monospecific *E. annectens* bonebed, we identified only thirteen bones with perforations that resembled tooth traces. Based on the criteria for tooth traces from previous works (S1 Fig), and the additional attention to context provided by the CT scan images, we found that one specific specimen, the surangular HRS05499, out of the thirteen fossils, had questionable tooth trace-like perforations (Fig 2). Twelve specimens manifested tooth traces: five ribs, five vertebrae, one radius, and one ulna. The lesions were classified into types of tooth traces: pit, puncture, score, and furrow. While some tooth traces were easily identified and classified, others were classified only because of their context and association with other traces on the same bone. From the twelve specimens with tooth traces, four specimens manifested patterns attributable to a named ichnotaxa. What follows is a description of each specimen and its associated perforation(s).

Questionable Perforations

We examined a surangular fossil HRS05499 with perforations/lesions resembling tooth traces. However, the context and shapes for the lesions, as well as bone association important for tooth trace identification, were ambiguous. Thus, we conducted further analysis to determine the diagnosis and classification of these perforations.

47 surangular specimens of *E. annectens* were identified within the bonebed. However, the left surangular HRS05499 is different from all the other surangulars found in the bonebed (Figs 2A and 2B). Unlike the other *E. annectens* surangulars, the HRS05499 has several polygonal perforations, resembling pits and punctures caused by bite marks from conical teeth. Shed crocodilian teeth of *Borealosuchus sternbergii* are found within the bonebed. Preliminary analysis of linear arrangement, spacing, and conical shape of the perforations matched with the tooth arrangement and spacing from a modern alligator maxilla (Fig 2C). However, the smooth edges and indetermined depth in several of the perforations are atypical. CT images reveal (following from lateral (rostral) to medial (lingual); Fig 3) that each of the perforations continues into the bone at different angles before collecting into a common area (Fig 3I), typical for bone foramina. Thus, we identified these perforations as foramen within the surangular. We also compared our specimen with other surangulars from different species found in other bone collections at the Raymond Alf Museum (RAM) and Black Hills Institute of Geological Research Inc. (BHI) and found one match, the surangular specimen BHI 4772 from a sub-adult *Torosaurus latus* known as Billy, (S3 Fig). Although larger than our specimen, the positioning, alignment and spacing of the foramina on the two specimens are nearly identical. Thus, based on the arrangement, depth, and smooth edges of the perforations, and comparisons with other

specimens, the perforations from the surangular HRS05499 are normal foramina found in a triceratopsini surangular.

Fig 2. Bone perforation on surangular HRS05499.

A) Lateral view of surangular. B) Medial view of surangular. Red arrows indicate different bone perforations and blue arrow indicates a different type of perforation. C) Comparison of bone perforations alignment and spacing of surangular with the tooth alignment and spacing of a modern crocodile maxilla.

Fig 3. CAT scanning of surangular HRS05499.

A-H) Sequential images in coronal view (due to crossing angle of perforations). Numbers indicating the perforations correspond to Figs 2A and 2B. Red arrows indicate perforations continuing deep into the bone. I) Rostral view of bone. Blue arrow corresponding to blue arrow on Fig 2B, indicates different types of perforation on which several of the smaller ones (red arrows) connect.

Tooth Trace Lesions

Ribs

Rib fragment HRS09477 has three tooth trace types: a large furrow on one surface and two punctures on the opposing surface (Figs 4A and 4B). Drag marks from the bite are associated with the puncture identified as #2 in Figs 4B and 4C. Ribs HRS03161, HRS09551 and HRS03387 have scores on the surface along their length. One rib fragment (HRS03161) features an initial bite puncture with associated score from tooth drag on the bone surface (Fig 4D). Specimen HRS09551 has two parallel aligned scores on the edge/surface of the rib fragment (Fig

4E), and specimen HRS03387 has three closely associated parallel scores on its midrib surface with the same orientation for the markings (Figs 4F and 4G). These scores also represent examples of the ichnotaxa *Knethichnus parallelum*. Lastly, rib HRS09954 has a well-defined long score on its proximal end surface (Fig 4H).

Fig 4. Tooth trace types on rib specimens.

A) Prominent furrow tooth trace on rib fragment HRS09477. B) Two puncture traces on rib fragment HRS09477 indicated by arrows on the opposing side of a furrow on Fig 4A. C) Magnified image of the puncture on HRS09477 indicated by arrow 2 on Fig 3B, indicating the pull and drag from the bite. D) Rib fragment HRS03161 with puncture and associated drag from the bite. E) Rib fragment HRS09551 with parallel scores. F) Rib HRS03387 with prominent scores and associated tooth trace ichnotaxon *Knethichnus parallelum*. G) Magnification of area indicated by the white box on figure 4F featuring prominent and detailed parallel *Knethichnus parallelum* traces. H) Rib HRS09954 with a long score trace indicated by the arrow.

Vertebrae

The tooth traces on the vertebrae were often located on the neural spine. Neural spine fragment HRS01295 has punctures on opposing surfaces (Figs 5A-5C), and one of the punctures shows a drag mark (Figs 5A and 5B). Neural spine fragment HRS03650 has a pronounced, curved, deep score with another shallower score on the opposing surface (Figs 5D and 5E). The neural spine from caudal vertebra HRS00428 (Fig 6A) has its distal end missing and associated reactive bony growth. Figs 6B and 6C also show two curved scores with serrations on the left surface of the caudal vertebra HRS00428. Neural spine HRS09830 has two parallel scores on the

right surface (Figs 6D and 6E). Unlike the other tooth traces found on neural spines, the caudal vertebra HRS00473 has three different tooth traces located on its centrum (Figs 6F-6H), two scores and one puncture on the opposing side.

Fig 5. Tooth trace types on neural spine fragments.

A) Punctures on opposing surfaces in neural spine HRS01295. The image only depicts one of the punctures indicated by the white box. An arrow indicates the location of the other puncture not visible in the image. B) A magnified view of the puncture within the white box area on Fig 5A. C) A view of the puncture opposing the puncture on Fig 5B and indicated by arrow on Fig 5A. D) Neural spine fragment HRS03650 with a score indicated by an arrow. E) A view of the opposing surface from HRS03650 (Fig 5D) shows a deep and curved score with associated tooth trace ichnotaxon *Linichnus serratus*.

Fig 6. Tooth trace types on caudal vertebrae.

A) Distal caudal vertebra HRS00428 (from middle-distal region of tail) with the distal end of the neural spine missing. Note bone proliferation on the distal neural spine. B) HRS00428 with curved scores on the neural spine associated with the tooth trace ichnotaxon *Linichnus serratus*. C) Magnification of the scores from the white box on figure 6B depicting details of the prominent *Linichnus serratus* traces. D) Neural spine HRS09830 from a caudal vertebra (middle-distal region of tail) with scores within the white box area. E) Magnification of the scores associated with the white box from Fig 6D. F) Distal caudal vertebra HRS00473 with arrows indicating tooth traces on the centrum. G) Score on HRS00473 associated with arrow “G” on Fig 6F. Note another score closer to the junction of the centrum with the neural spine. H) Puncture on the centrum HRS00473 associated with arrow “H” on Fig 6F.

Radius and Ulna

Additionally, tooth traces are also observed on two different forelimb bones. Specimen HRS13582 (Fig 7), identified as a radius, has six noticeable scores: one score with associated parallel striations indicative of the ichnotaxon *Knethichnus parallelum* (Fig 7B), two parallel linear scores (Fig 7C), and lastly three slightly curved, deep scores with associated serrations indicative of the ichnotaxon *Linichnus serratus* (Figs 7D and 7F). Specimen HRS10076, identified as ulna (Fig 8), also shows tooth traces: eight pits and two sets of two parallel scores located at the distal end (Figs 8B and 8C).

Fig 7. Tooth traces on radius HRS13582.

A) Radius with several scores indicated by white boxes and corresponding letters. B) Magnification of white box B depicting score with also associated ichnotaxon for *Knethichnus parallelum*. C) Magnification of white box C depicting parallel scores. D-F) Magnification of associated white boxes from Fig 7A with deep scores for tooth traces and associated ichnotaxon *Linichnus serratus* with serrations especially clear with associated score in Fig 7D.

Fig 8. Tooth traces on ulna HRS10076.

A) Ulna with black boxes indicating areas with tooth traces. B-C) Magnification of corresponding boxes from Fig 8A. White arrows indicate pits and red arrows indicate parallel scores.

Tooth Trace Ichnotaxa

The ichnotaxa *Linichnus serratus* and *Knethichnus parallelum* [16] for score and furrow traces have the potential to be an indicator of the inflictor to the species level. Because *Linichnus serratus* and *Knethichnus parallelum* traces have denticle marks, only carnivores with ziphodont teeth can inflict such traces. The spacing between the parallel lines found in *Knethichnus parallelum* is attributable to the denticles of the inflictor dragging on the surface of a bone during a bite [16]. Thus, specific striation spacing of the trace suggests the identification of specific inflictor's tooth. A *Linichnus serratus* trace is identified as a curved score with serrated morphology and U- or V-shaped geometry in cross-section. It can be diagnostic to species level when the trace has more than one well-defined and well-preserved serration set, indicative of denticle spacing.

The ichnotaxon *Knethichnus parallelum* is identified in two different specimens. The scores from rib HRS03387 have three parallel *Knethichnus parallelum* traces along the medial surface of the rib (Figs 4F, 4G, 9A). The parallel striations from the three traces on the medial surface of the rib are defined and well-preserved. The density of these striations is three striations per 2 mm (Fig 9A) in all the different traces on the bone. Likewise, one *Knethichnus parallelum* trace is also identified on radius HRS13582 with the parallel striation density of three striations per 2 mm (Figs 7B and 9B). Additionally, three *Linichnus serratus* traces are also identified (Figs 7D-7F). Details of preserved serrations in *Linichnus serratus* traces are essential to determine the denticle density of the inflictor. Although serrations are observed in the three *Linichnus serratus* traces on specimen HRS13582, only one of these traces (Fig 7D) has enough detail to determine the density of two serrations per 2 mm.

Fig 9. Striation density associated with scores of ichnotaxon *Knethichnus parallelum*.

A) Scores on rib HRS03387 with striation density of three per 2 mm. B) Score on radius HRS13582 with striation density of three per 2 mm.

Linichnus serratus is also identified on two additional fossils in our collection. The caudal vertebra HRS00428 has two curved scores located on the proximal right lateral surface of the neural spine (Figs 6B and 6C). The serration density observed is two per 2 mm. The neural spine fragment HRS03650 also presents two well-preserved examples of *Linichnus serratus* on the opposing sides of the spinal fragment (Figs 5D and 5E). However, the score with the associated traces on the left surface (Fig 5D) is less prominent and deep than the traces associated with the score on the right surface (Fig 5E) indicating serration density of two per 2 mm.

Taxon identification by ichnotaxa

Because some of the bones in the collection manifested tooth traces that suggested a ichnotaxa, we selected serrated teeth from the same bonebed that could potentially identify probable predator. The taxon with ziphodont teeth represented within the bonebed are the tyrannosaurids *Tyrannosaurus rex* and *Nanotyrannus lancensis* (which may be a juvenile *T. rex,* [see 22, 23]) the troodontid *Pectinodon bakkeri*, and the dromaeosaurid *Acheroraptor temertyorum*. We present in Table 1 the measurements of denticle density according to taxon from the bonebed (and other comparative bonebeds of the Maastrichtian Lance Formation). Striation densities on *Knethichnus parallelum* traces must be equivalent or less than the denticle density found on the inflictor's tooth [24]. Denticle density measurement from the teeth of the animals, other than *T. rex*, associated with the bonebed have higher densities, which indicates that the only capable inflictor of the *Knethichnus parallelum* traces was *T. rex.* The density of the

serrations from the *Linichnus serratus*, two serrations per 2 mm, on the radius HRS13582 (also associated with *Knethichnus parallelum)* confirms traces on the bone to be from *T. rex* bites. Serration density on the neural spines HRS00428 and HRS 03650 is identical to the ones found in the radius HRS13582. Thus, it is parsimonious to suggest that the traces attributed to the two different ichnotaxa are caused by the same species, in this case *T. rex*.

Table 1. Tooth measurements for denticle density (per 2 mm) from theropod taxa known associated with examined bonebed of the Lance Formation.

Taxon	*Acheroraptor*	*Nanotyrannus*	*Tyrannosaurus*	*Pectinodon*
AVG MD/2 mm	6.1	5.2	3.2	-
AVG DD/2 mm	6.1	5.1	3.4	6.1
Range MD/2 mm	5.4-6.8	4.1-6.3	2.1-4.3	-
Range DD/2 mm	5.3-6.9	4.1-6.1	2.5-4.3	4.9-7.3
McLain et al. 2018	6.12/5.99	6.1/5.08	3.22/3.79	5.19*
Smith et al. 2005			3.7/3.9	

*Referred to as *Troodon*, DD/2mm

Abbreviations: AVG= average; MD/2 mm= mesial denticle density per 2 mm; DD/2 mm= distal denticle density per 2 mm; D/2 mm= denticle density per 2 mm

Discussion

The criteria for attributing a lesion to bite marks is precise but not exhaustive. When bones are associated and/or articulated it is easier to determine whether or not the perforations are lesions representative of tooth traces, due to the overall anatomical context of the animal. However, when examining bones that are disarticulated and dissociated, such as the ones within this study's bonebed (MNI=19, left surangular), it is crucial to look for clues in each individual bone, within the overall context of the bones, within the bonebed, and at the characteristics of the

specific animal species. We have considered the following factors to be important in the analysis of whether bone perforations are tooth traces: a) shape; b) length and width; c) edge; d) depth surface texture; e) depth; f) channeling between perforations when more than one is present; g) location; h) association with bone remodeling; i) general bone morphology and morphology specific to the taxon; j) communality of perforation type with bone association. In some cases, the cause of these perforations attributable to tooth traces is clear, such as in the case of the rib HRS03387 (Fig 4F), neural spine fragment HRS03650 (Fig 5E), and radius HRS13582 (Fig 7) because of the striations and serrations left on the bone by the denticles of the predator's tooth.

However, in most cases the cause of a lesion is not immediately clear. Therefore, a combination and association of different factors give a contextual understanding that becomes a powerful tool for interpretation on cause or origin. Below, we discuss different combinations and associations of factors that contributed to our interpretation for tooth traces in the bones from this study.

Contextual Factors Important for Tooth Trace Criteria

Determining perforation depth and the texture of the perforation edges, and the directly contacted surface (impacted surface) is important because it provides information on natural morphology, bone reaction associated with pathology, or post-mortem erosion. Perforation with a smooth surface usually suggests fresh/malleable or responsive bone. On specimen HRS09477 (Fig 4C) and HRS01295 (Fig 5B), the location and appearance of the perforations are atypical for this type of bone. The smoothness of the texture along the edges of the perforation and impacted surface suggests fresh/malleable bone or early stages of bone remodeling. In both specimens, the pull and dragging along the edges of perforations, typically representing tooth

traces, is also observed. Thus, we concluded that these perforations are tooth traces from premortem bites with sometime for early stages of bone remodeling followed by death, or most likely perimortem bites.

However, perforations with smooth edges also may indicate a normal osteological feature such as foramina. If one is not familiar with the specific osteological features in a specimen, especially in extinct species, these perforations could potentially be mistakenly identified as a tooth trace. The surangular HRS05499 (Fig 2) is a great example of the need for further investigation of such perforations. The perforations shape and arrangement initially could be mistakenly identified as tooth trace lesions caused by a crocodile bite. However, the context of the "bite" combined with the texture of the perforations and CT scan results, indicated that the perforations were not tooth traces but a natural bony feature for the surangular, most likely foramina, not identified into any of the other surangulars in the *E. annectens* bonebed. This is supported by previous findings that the surangulars of hadrosaurs do not have foramina [25]. Although our examined bonebed is largely from *E. annectens*, our findings with the surangular HRS 05499 let us to further investigate the surangulars from other contemporary animals and consequentially find a larger but exact match, the surangular BHI 4772 from the sub-adult *Torosaurus latus.* Thus, details in the texture, depth and context for perforations are important for etiology. We also suggest that for future study, the examination of more surangular bones from ceratopsids is necessary because the presence of foramina only in surangulars from Triceratopsini is potentially diagnostic for the particular tribe or even for the specific genus *Torosaurus*.

Perforations with smooth edges can also suggest channel formation as the bone responds to infection, which is often associated with complications from trauma. In mammal bones,

infection is indicated by the presence of noticeable openings from channels called cloacae necessary for pus drainage during the healing process [26, 27]. The process is different in reptiles and birds, where a casseating granuloma (a more solid substance) forms at the infection site [28-30] A more mammal-like response to infection has been suggested to occur in dinosaurs [20, 31-33]. The organization and characteristics of the lesions observed in the previously reported neural spine HRS07948 [33], including its association with the evident misalignment and bone proliferation, indicates fracture of the neural spine with an infectious response. Thus, we conclude that it is important to consider the context and morphological details of perforations.

Joint related pathologies can also leave a perforation or depression on the articular surface of bones. In such cases, we observe lesions to also have smooth edges and bone response in common joint surfaces, often associated with sclerotic bone. Previously reported analysis of bones from this study’s bonebed illustrates caudal vertebrae HRS07804, HRS07887, HRS01254 and HRS05808 to have perforations in the articular surface of the centrum [33]. When considering only the descriptive criteria for tooth traces in fossils (S1 Fig), these specimens could be mistakenly identified as a tooth trace. However, the context of the lesions makes these perforations questionable candidates for a tooth trace. The smooth edges, along with the location of the perforation (on the lateral aspect of the joint surface of the centrum), suggests an unlikely place for a bite. CT scans showed bone reaction associated with lesions [33] thus suggesting pathology. These types of lesions may be characteristic of mechanical stress due to frequent tail injury affecting the joint surface of caudal vertebral centra. Rothschild and Tanke [34] have previously reported other joint diseases in hadrosaur bones and suggested genetic predisposition.

In this study, we also report several bones with relatively unambiguous bite marks. These types of lesions, when associated with extending striations or serrations, are recognized as

ichnotaxa. The ichnotaxa in this study, *Knethichnus paralellum* (Fig 8) and *Linichnus serratus* (Figs 4E, 5C, 6D), have striation and serration density of two per 2 mm. Although we have measured all the possible ziphodont teeth from animals associated with the bonebed, the only animal with the denticle density average (or range of density) compatible with the striation or serration density is *T. rex*. The other taxa had more denticles per mm and their denticle density range was always higher than four denticles per 2 mm. The *Knethichnus paralellum* and *Linichnus serratus* traces in this study did not present signs of bone remodeling associated with the traces, suggesting that the bites occurred peri-mortem or post-mortem. While we cannot be conclusive on the identification of the inflictors associated with all the tooth traces in this study, we have evidence that *T. rex* bit the bones of some of these animals. On the radius HRS13582, the spacing of the very definite linear parallel scores (10 mm apart) is characteristic of *Knethichnus paralellum* and *Linichnus serratus* traces (Fig 6C). These scores might also indicate the interdental spacing of the inflictor, in this case *T. rex*. Similarly, the ulna HRS10076 has also definite linear and parallel scores with similar interdental spacing of 10 mm (Figs 7B and 7C). However, the ulna scores do not have associated ichnotaxa traces diagnostic for the nature of the inflictor. Nonetheless, it is reasonable to identify (by comparison with the radius HRS13582) *T. rex* as the inflictor of the bite in the ulna. It is important to note that many of these predators shed and replaced their teeth, and the angle of bite varies. Therefore, we can only be precise on the identification of the bite inflictor when the parallel scores are in association with tooth trace ichnotaxa.

There are other caveats to consider in tooth trace identification. First, any kind of trace in cancellous bone is always questionable. Without the cortical surface present, it is very difficult to have any idea of what happened to cause the perforation or groove on cancellous bone. In this

study, we did not find any lesions affecting cancellous bone. Thus, we may be underestimating the frequency of bite marks from this bonebed due to the difficulty of identification. Unless a tooth is found lodged in the trace [8, 35], all traces in cancellous bone are ambiguous. There are extreme cases of traces in cancellous bone associated with shearing. In bone shearing there is a complete removal of a bone piece from a bite, thus making it challenging to preserve any trace.

However, there may be possible cases of shearing where signs of a bite can be concluded. Carpenter [36] reported a hadrosaur caudal neural spine missing its distal end. The neural spine also has associated bone proliferation. Other caudal neural spines in connected vertebrae had tooth traces. Thus, the author concluded that the missing and injured neural spine resulted from a bite taken out of the animal during his life and bone proliferation is most likely a response to an infection acquired from the bite [36]. Even though no tooth or tooth traces were associated with the neural spine, it is tempting to hypothesize this case to be an example of “bite trace” with no direct evidence. This example may also indicate a case of pseudarthrosis which resulted from a failed fracture union [20].

In our study, the neural spine HRS00478 (Fig 5A) represents a similar specimen as previously described by Carpenter [36]. The bone is also missing its distal end with bone proliferation. A fracture, not resulting from a bite, is unlikely since only a small segment of the distal neural spine is missing. In the same specimen, we also have unhealed tooth traces (Fig 5C) on the proximal segment of the bone. Therefore, we conclude that this specimen represents unrelated examples of lesions caused by bites. While the lesion associated with the distal neural spine occurred while the animal was alive and with time to heal, the bite associated with the scores occurred either peri-mortem or post-mortem.

Another caveat to consider in tooth trace identification pertains to traces found in highly-fractured bones. Tooth traces can be camouflaged or destroyed within fractured regions that can often occur in scavenging or predation. It is important to note that bone fractures during trampling, transport or post-burial compaction fractures can directly impact the preservation of tooth traces since bitten areas might be structurally weaker.

An additional layer of complexity when considering the identification for a perforation is the similarity between pit- or puncture-like tooth traces and other perforations caused by bioerosion. It is important to be familiar with the different types of ichnotaxa for bone bioerosion [37]. Although most cases of bioerosion in bones are usually unambiguous, the more pit/puncture like perforations might be mistaken for a tooth trace. Such cases are bivalve macroborings on bones [38, 39], and chambers created by necrophagous larvae [37, 40-42] that might have the shape of a tooth trace (puncture or pit). Their shape might be very similar to a puncture or a pit caused by a tooth, especially in isolated instances. The gross appearance of the edges of these postmortem bioerosive perforations also might appear smooth and resemble a tooth trace made while the bone was still fresh. Thus, histological examination of a perforation and its associated context is important to determine etiology.

Finally, when considering different types of bone perforations as tooth traces it is important to think about the impact of excavation and fossil preparation tools on bone surfaces. After experiments with commonly used tools in excavation and preparation of fossil bones, it was determined that tool marks could be distinguished from authentic tooth traces [43]. Tool marks often left shallower depressions, even when more force was applied by the examiner. Also, the shape of the depressions created by tools usually were of uneven patterns and with flaking of the edges [43].

As a result of this study, we adapted the guidelines for the identification of tooth traces (Fig 10) to incorporate many different types of bone perforations. Such perforations can result from natural bone morphology such as foramina in addition to natural bone response to infection or frequency of mechanical stress on joint surfaces. We have also incorporated previous work on tooth trace types and identification (S1 Fig) including ichnotaxa for tooth traces. Although shearing of bone is not a type of bone perforation, its context can be considered as evidence for a bite. Thus, we also integrated shear into our guideline. However, it is important to note that shearing is often difficult to interpret and quantify due to the lack of direct evidence. Finally, we also consider post-mortem perforations such as bioerosive process, especially macroboring due to the larger size and shape that might potentially be mistaken for another type of bone perforation. This new guide (Fig 10) for the different types of bone perforations thus refines the criteria for identification of tooth traces and will be helpful for correctly labeling bone perforations.

Fig 10. Types of bone perforation with contextual application.

Conclusion

The correct identification of the different types of bone perforations is necessary especially when examining for tooth traces. Herein, we identified and classified different types of tooth traces from an *Edmontosaurus annectens* bonebed within the Lance Formation, WY. We described several types of traces and associated features, such as evidence for bone remodeling, different types of tooth trace ichnotaxa useful for inflictor identification and shearing when there is no direct evidence of a tooth trace. We also identified and determined the nature of other types

of bone perforations that potentially could be mistaken for tooth traces such as foramina, cloaca, cartilaginous inclusions/bone necrosis, and bioerosion. Consequently, we developed a guide for the identification of bone perforations and associations to tooth trace criteria. Further investigations of more specimens from this bonebed will continue, and we expect more examples of tooth traces and other types of bone perforations that will further refine the guide (Fig 10). We also invite other researchers to collaborate on interpretations and identifications of the different types of bone perforations. Refining the criteria for tooth traces in the context of bone perforation is important and will help interpretations of animal biting behavior as well as our understanding of dinosaur paleopathology, natural bone morphology and biomechanics.

Acknowledgments

We thank the Hanson Ranch owners for facilitating this research. We also thank all the students and volunteers for excavating and preparing the recovered specimens and especially Emily Manzano for assistance on tooth measurements. We thank Pete Larson for giving access to the museum collection at the Black Hills Institute of Geological Research. We also thank Andrew Farke and Gabriel Santos for access to the museum collection at Raymond M. Alf Museum of Paleontology and assistance in photography and photographic equipment. Finally, we thank Leroy Leggit and Daniel Kido at Loma Linda University for assisting with the radiographs.

References

1. Tanke D, Currie PJ. Head-biting behavior in theropod dinosaurs: paleopathological evidence. GAIA. 1998; 15: 167-84
2. Brown CM, Currie PJ, Therrien F. Intraspecific facial bite marks in tyrannosaurids provide insight into sexual maturity and evolution of bird-like intersexual display. Paleobiology. 2022 Feb;48(1):12-43.
3. Rogers RR, Krause DW, Curry Rogers K. Cannibalism in the Madagascan dinosaur *Majungatholus atopus*. Nature. 2003 Apr 3;422(6931):515-8.
4. Drumheller SK, McHugh JB, Kane M, Riedel A, D'Amore DC. High frequencies of theropod bite marks provide evidence for feeding, scavenging, and possible cannibalism in a stressed Late Jurassic ecosystem. PLoS One. 2020 May 27;15(5): e0233115.
5. Longrich NR, Horner JR, Erickson GM, Currie PJ. Cannibalism in *Tyrannosaurus rex*. PLoS one. 2010 Oct 15;5(10): e13419.
6. McLain MA, Nelsen D, Snyder K, Griffin CT, Siviero B, Brand LR, Chadwick AV. Tyrannosaur cannibalism: a case of a tooth-traced tyrannosaurid bone in the Lance Formation (Maastrichtian), Wyoming. Palaios. 2018 Apr 24;33(4):164-73.
7. Dalman SG, Lucas SG. New evidence for cannibalism in tyrannosaurid dinosaurs from the Upper Cretaceous (Campanian/Maastrichtian) San Juan Basin of New Mexico. New Mexico Museum of Natural History and Science Bulletin. 2021 Apr 1;82:39-56.
8. Currie PJ, Jacobsen AR. An azhdarchid pterosaur eaten by a velociraptorine theropod. Canadian Journal of Earth Sciences. 1995 Jul 1;32(7):922-5.
9. Noto CR, Main DJ, Drumheller SK. Feeding traces and paleobiology of a Cretaceous (Cenomanian) crocodyliform: example from the Woodbine Formation of Texas. Palaios. 2012 Feb 1;27(2):105-15.

10. Lei R, Tschopp E, Hendrickx C, Wedel MJ, Norell M, Hone DW. Bite and tooth marks on sauropod dinosaurs from the Morrison Formation. PeerJ. 2023 Nov 14;11: e16327.

11. Binford LR. Patterns of bone modifications produced by nonhuman agents. Bones: ancient men and modern myths. San Diego, California: Academic press; 1981. pp. 35-86.

12. Njau JK, Blumenschine RJ. A diagnosis of crocodile feeding traces on larger mammal bone, with fossil examples from the Plio-Pleistocene Olduvai Basin, Tanzania. Journal of Human Evolution. 2006 Feb 1;50(2):142-62.

13. Pobiner BL, DeSilva J, Sanders WJ, Mitani JC. Taphonomic analysis of skeletal remains from chimpanzee hunts at Ngogo, Kibale National Park, Uganda. Journal of Human Evolution. 2007 Jun 1;52(6):614-36.

14. Pobiner B. Paleoecological information in predator tooth marks. Journal of Taphonomy. 2008;6(3-4):373-97.

15. Mikuláš R, Kadlecová E, Fejfar O, Dvořák Z. Three new ichnogenera of biting and gnawing traces on reptilian and mammalian bones: a case study from the Miocene of the Czech Republic. Ichnos. 2006 Sep 1;13(3):113-27.

16. Jacobsen AR, Bromley RG. New ichnotaxa based on tooth impressions on dinosaur and whale bones. Geological Quarterly. 2009;53(4):373-82.

17. Rothschild BM, Molnar RE. Tyrannosaurid pathologies as clues to nature and nurture in the Cretaceous. In: Larson PL, Carpenter K, editors. *Tyrannosaurus rex*, the tyrant king. Bloomington, Indiana: Indiana University Press; 2008. pp. 287-304.

18. Rega EA, Brochu CA. Paleopathology of a mature *Tyrannosaurus rex* skeleton. Journal of Vertebrate Paleontology. 2001 Aug 22;21(3):92.

19. Wolff ED, Salisbury SW, Horner JR, Varricchio DJ. Common avian infection plagued the tyrant dinosaurs. PLoS One. 2009 Sep 30;4(9): e7288.

20. Rega EA. Disease in Dinosaurs. In: Brett-Surman MK, Holtz TR, Farlow JO, editors. The Complete Dinosaur. Bloomington, Indiana: Indiana University Press; 2012. pp. 666-711.

21. Snyder K, McLain M, Wood J, Chadwick A. Over 13,000 elements from a single bonebed help elucidate disarticulation and transport of an *Edmontosaurus thanatocoenosis*. PLoS One. 2020 May 21;15(5): e0233182.

22. Carr TD. A high-resolution growth series of Tyrannosaurus rex obtained from multiple lines of evidence. PeerJ. 2020 Jun 4;8: e9192.

23. Longrich NR, Saitta ET. Taxonomic status of Nanotyrannus lancensis (Dinosauria: Tyrannosauroidea)—a distinct taxon of small-bodied tyrannosaur. Fossil Studies. 2024 Mar;2(1):1-65.

24. D'Amore DC, Blumenschine RJ. Using striated tooth marks on bone to predict body size in theropod dinosaurs: a model based on feeding observations of *Varanus komodoensis*, the Komodo monitor. Paleobiology. 2012 Jan;38(1):79-100.

25. Horner HR, Weishampel DB, Forster CA. Hadrosauridae. In: Weishampel DB, Dodson P, Osmólska H, editors. The Dinosauria. 2nd ed. University of California Press; 2004. pp. 438-463.

26. Aghababian RV, Conte Jr JE. Mammalian bite wounds. Annals of emergency medicine. 1980 Feb 1;9(2):79-83.

27. de Souza Barbosa FH, de Oliveira Porpino K, Fragoso AB. Osteomyelitis in Quaternary mammal from the Rio Grande do Norte State, Brazil. Quaternary International. 2013 Jun 19;299:90-3.

28. Montali RJ. Comparative pathology of inflammation in the higher vertebrates (reptiles, birds and mammals). Journal of comparative pathology. 1988 Jul 1;99(1):1-26.

29. Antinoff N. Osteomyelitis in reptiles. Proc ARAV. 1997:149-52.

30. Tell LA, Ferrell ST, Gibbons PM. Avian mycobacteriosis in free-living raptors in California: 6 cases (1997–2001). Journal of Avian Medicine and Surgery. 2004 Mar;18(1):30-40.

31. Hanna RR. Multiple injury and infection in a sub-adult theropod dinosaur *Allosaurus fragilis* with comparisons to allosaur pathology in the Cleveland-Lloyd Dinosaur Quarry Collection. Journal of Vertebrate Paleontology. 2002 Mar 14;22(1):76-90.

32. Xing L, Rothschild BM, Randolph-Quinney PS, Wang Y, Parkinson AH, Ran H. Possible bite-induced abscess and osteomyelitis in *Lufengosaurus* (Dinosauria: sauropodomorph) from the Lower Jurassic of the Yimen Basin, China. Scientific reports. 2018 Mar 22;8(1):5045.

33. Siviero BC, Rega E, Hayes WK, Cooper AM, Brand LR, Chadwick AV. Skeletal trauma with implications for intratail mobility in *Edmontosaurus annectens* from a monodominant bonebed, Lance Formation (Maastrichtian), Wyoming USA. Palaios. 2020 Apr 16;35(4):201.

34. Rothschild BM, Tanke DH. Osteochondrosis in Late Cretaceous Hadrosauria: a manifestation of ontologic failure. In: Carpenter K, editor. Horns and Beaks: Ceratopsian and Ornithopod Dinosaurs. Bloomington, Indiana: Indiana University Press; 2007. pp. 171-183.

35. DePalma RA, Burnham DA, Martin LD, Rothschild BM, Larson PL. Physical evidence of predatory behavior in *Tyrannosaurus rex*. Proceedings of the National Academy of Sciences. 2013 Jul 30;110(31):12560-4.

36. Carpenter K. Evidence of predatory behavior by carnivorous dinosaurs. GAIA: 1998(15):135-44.

37. Pirrone CA, Buatois LA, Bromley RG. Ichnotaxobases for bioerosion trace fossils in bones. Journal of Paleontology. 2014 Jan;88(1):195-203.

38. Tapanila L, Roberts EM, Bouare ML, Sissoko F, O'LEARY MA. Bivalve borings in phosphatic coprolites and bone, Cretaceous–Paleogene, northeastern Mali. Palaios. 2004 Dec 1;19(6):565-73.

39. Wilson MA. Macroborings and the evolution of marine bioerosion. In: Miller III W, editor. Trace Fossils: Concepts, Problems, Prospects. Oxford, United Kingdom: Elsevier Science; 2007. pp. 350-361.

40. Lima FCGD. Alterações ante mortem e pseudopatologias em exoesqueleto de cingulados pleistocênicos de grande porte da região intertropical brasileira [Master’s thesis]. Mossoró, Brazil: Universidade Federal Rural do Semi-Árido; 2017. Portuguese.

41. Odes EJ, Parkinson AH, Randolph-Quinney PS, Zipfel B, Jakata K, Bonney H, Berger LR. Osteopathology and insect traces in the *Australopithecus africanus* skeleton StW 431. South African Journal of Science. 2017 Feb;113(1-2):1-7.

42. Zanetti NI, Ferrero AA, Centeno ND. Depressions of *Dermestes maculatus* (Coleoptera: Dermestidae) on bones could be pupation chambers. The American Journal of Forensic Medicine and Pathology. 2019 Jun 1;40(2):122-4.

43. Siviero BT, McLain MA, Nelsen D, Brand LR, Chadwick AV. Refinement of tooth trace criteria through experimentation and literature review. Geological Society of America Abstracts with Programs. 2017;49(6):84-49.

Supporting information

S1 Fig. Criteria for tooth traces. Compilation of literature review of tooth trace classification (modified after Binford 1981; Mikuláš et al 2006; Njau and Blumenschine 2006; Pobiner et al 2007; Pobiner 2008; Jacobsen and Bromley 2009).

S2 Fig. Tooth denticle density measurements. *Tyrannosaurus rex* tooth HRS06541 is used for illustration purpose.

S3 Fig. Surangular of a sub-adult *Torosaurus latus* BHI 4772. Numbers indicate individual foramen according to Fig 1.

Figure 1

Click here to access/download;Figure;Fig 1.tif

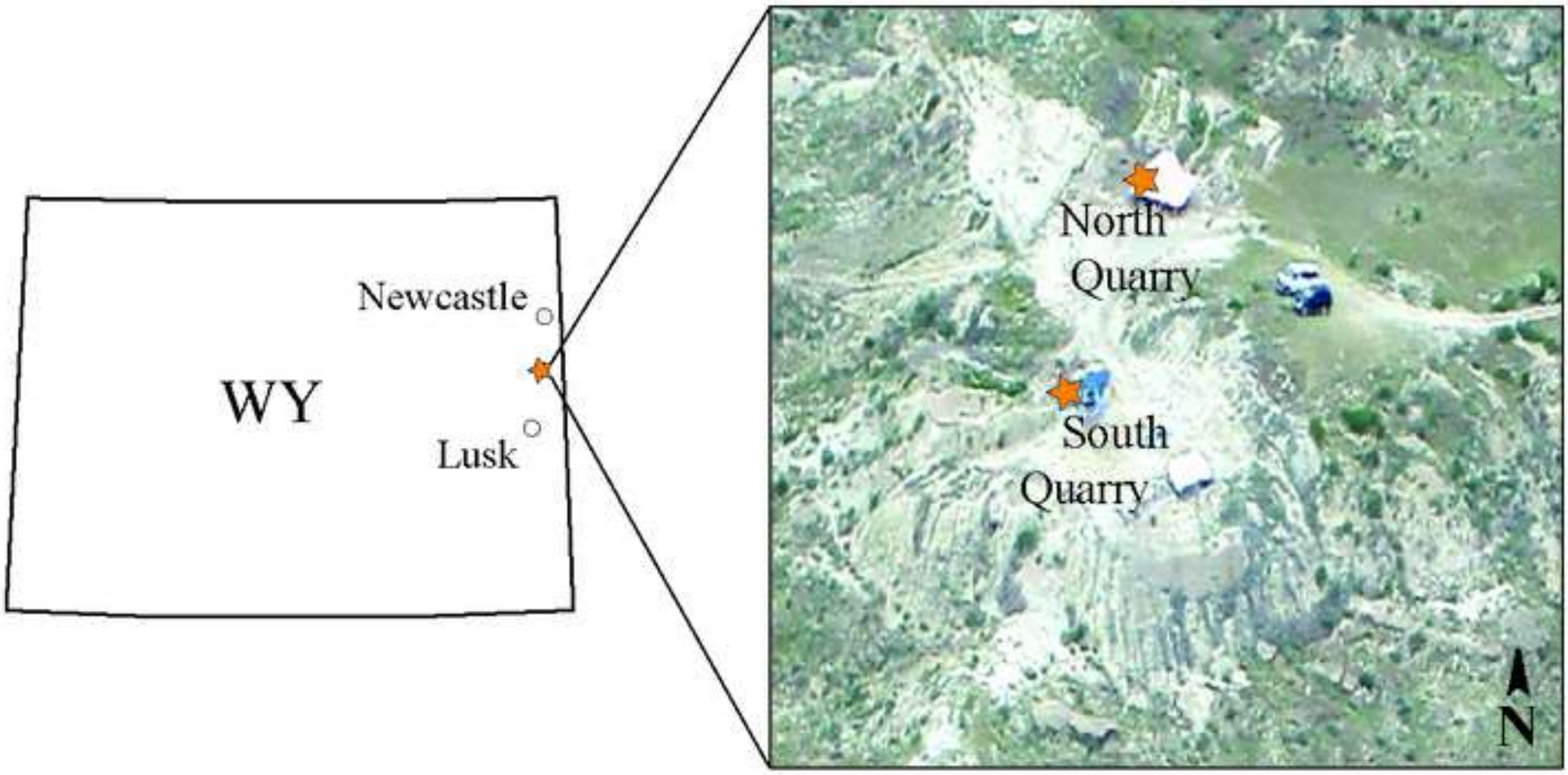

Figure 2

Click here to access/download;Figure;Fig 2.tif

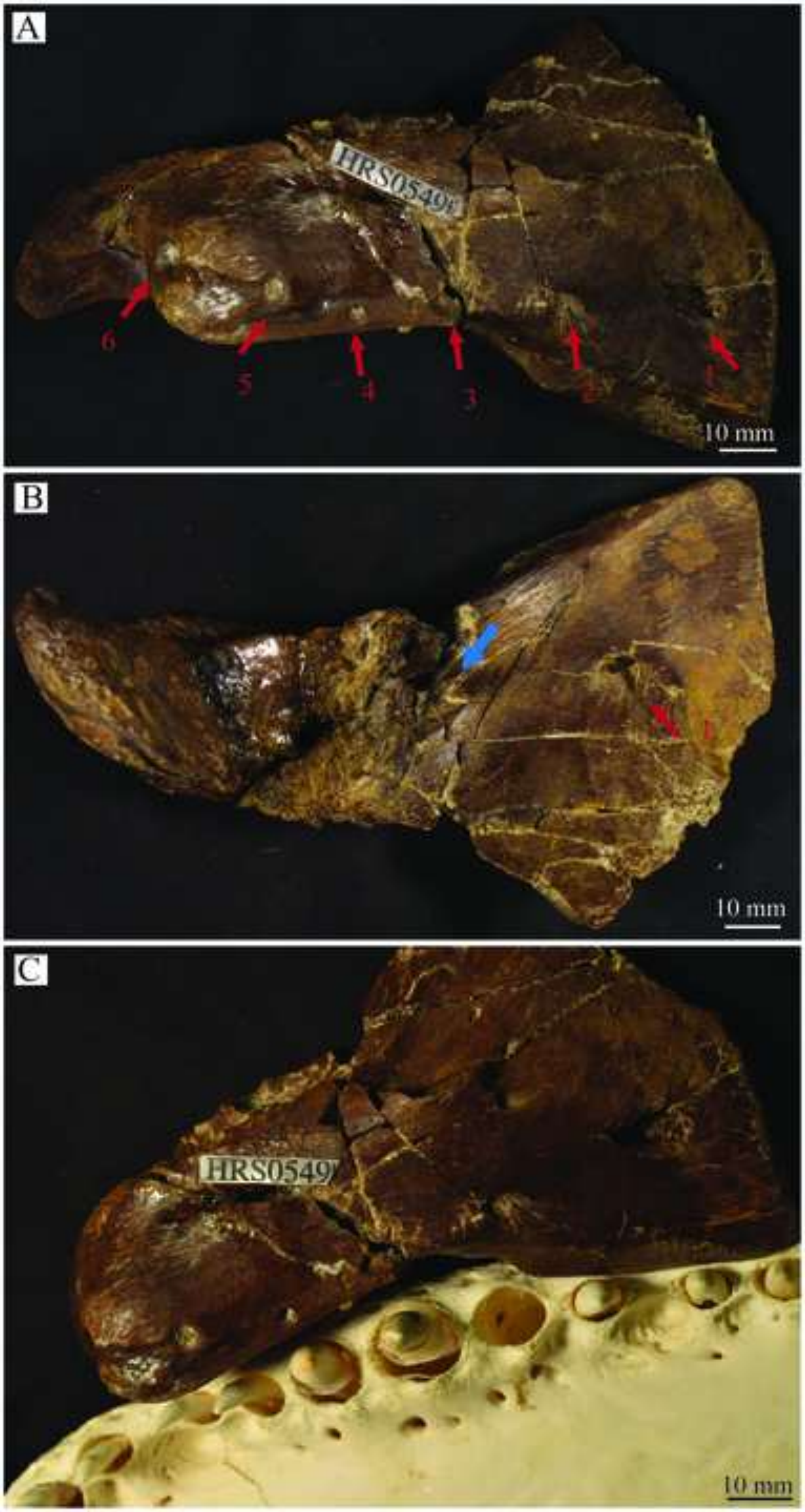

Figure 3

Click here to access/download;Figure;Fig 3.tif

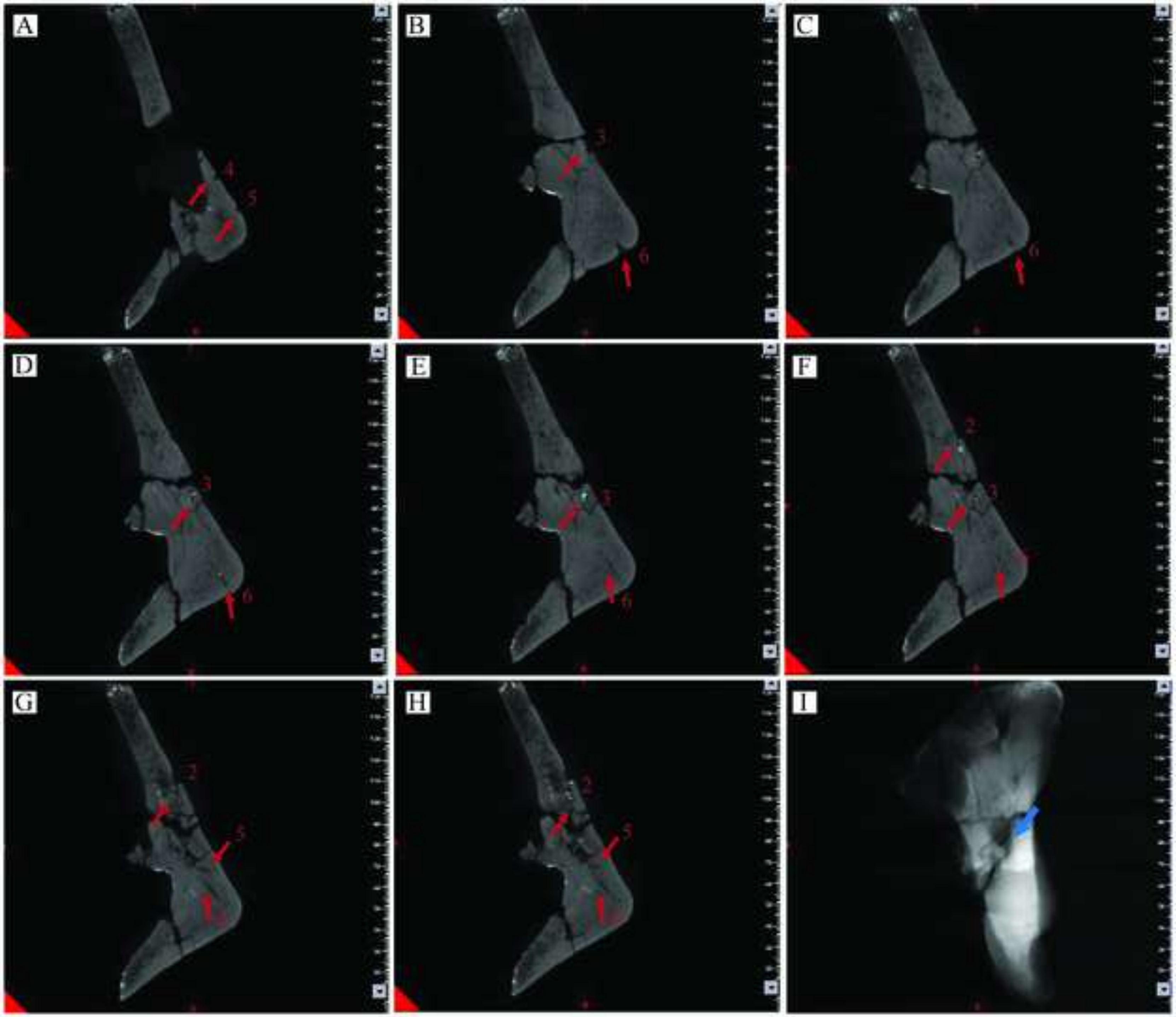

Figure 4

Click here to access/download;Figure;Fig 4.tif

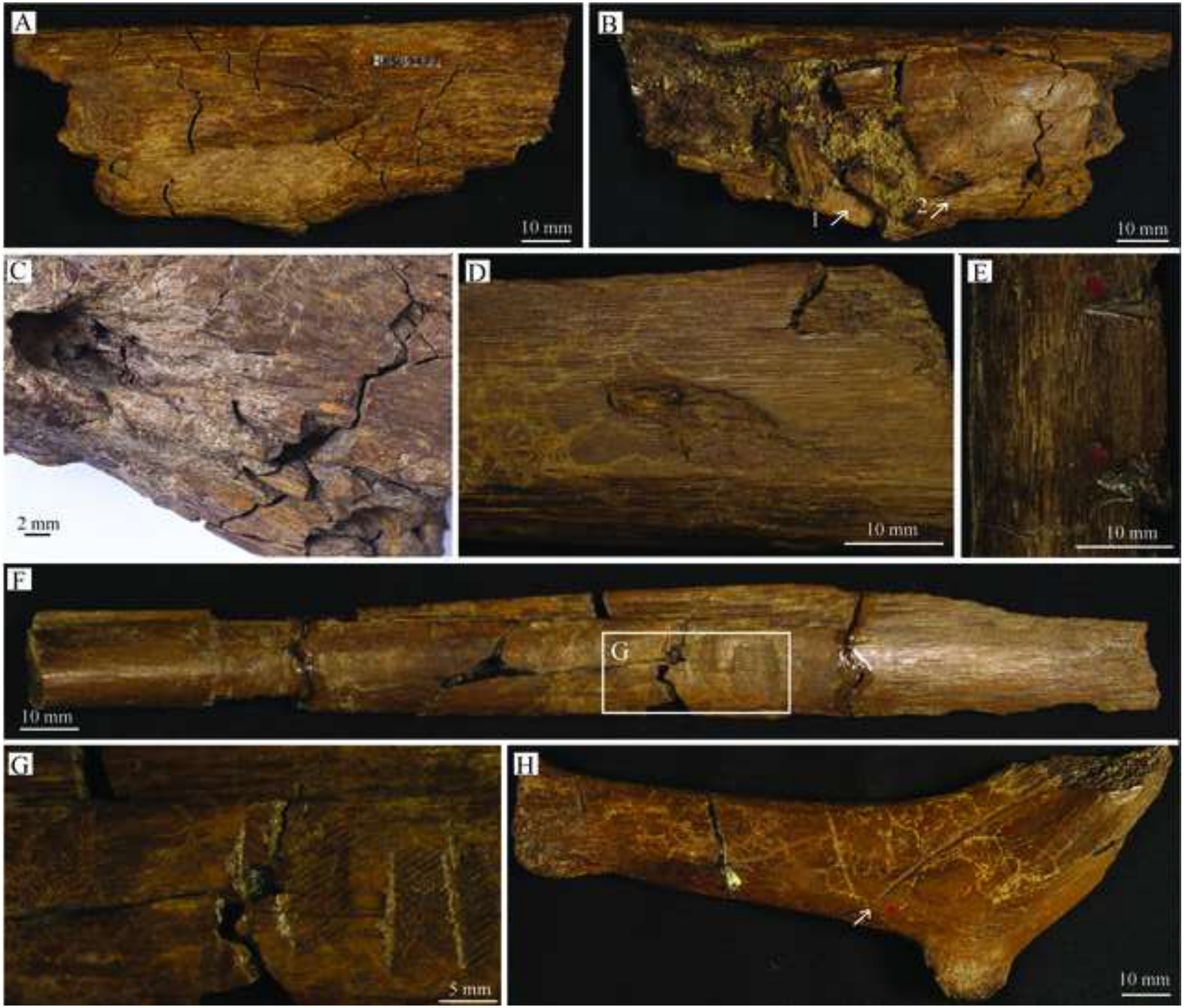

Figure 5

Click here to access/download;Figure;Fig 5.tif

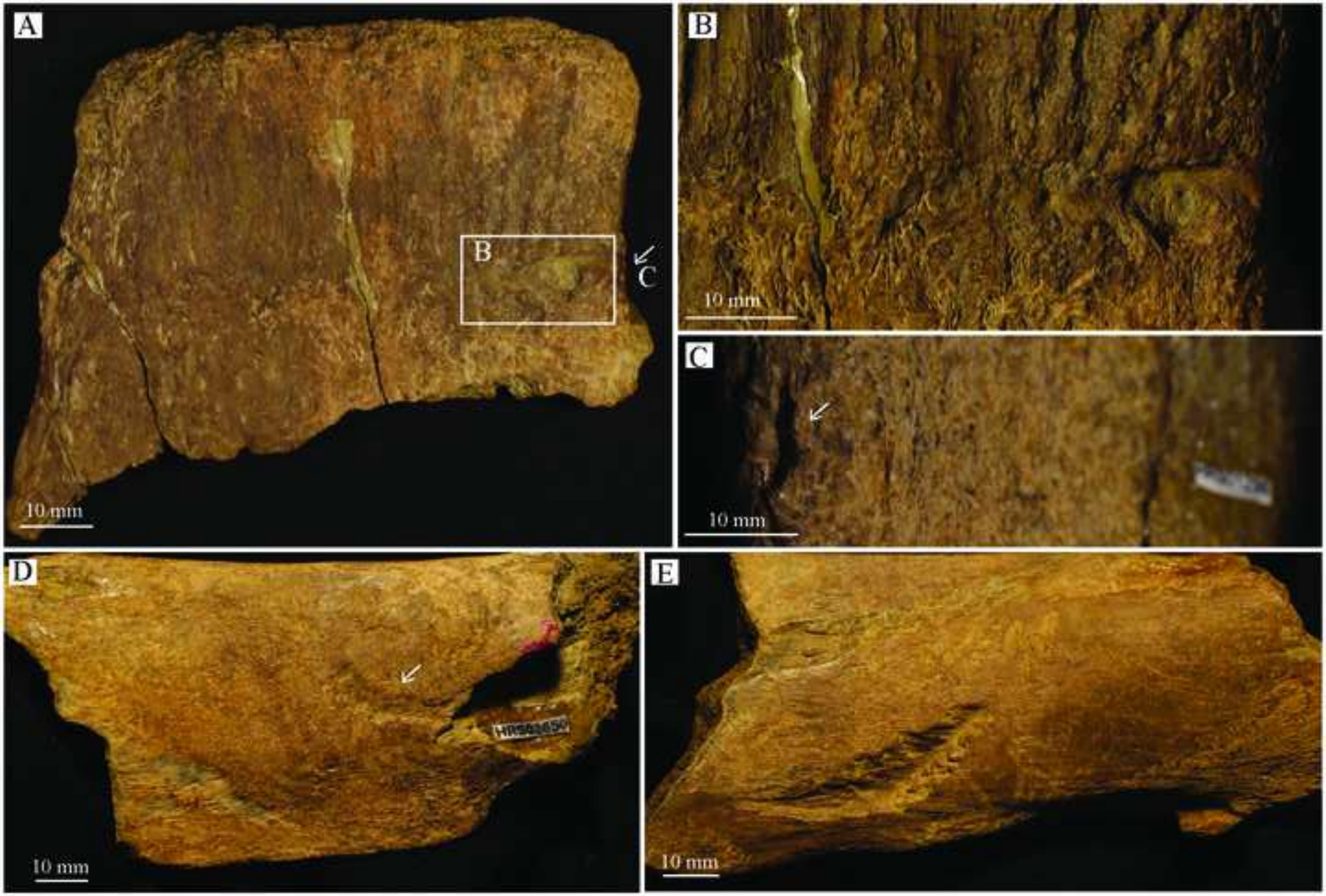

Figure 6

Click here to access/download;Figure;Fig 6.tif

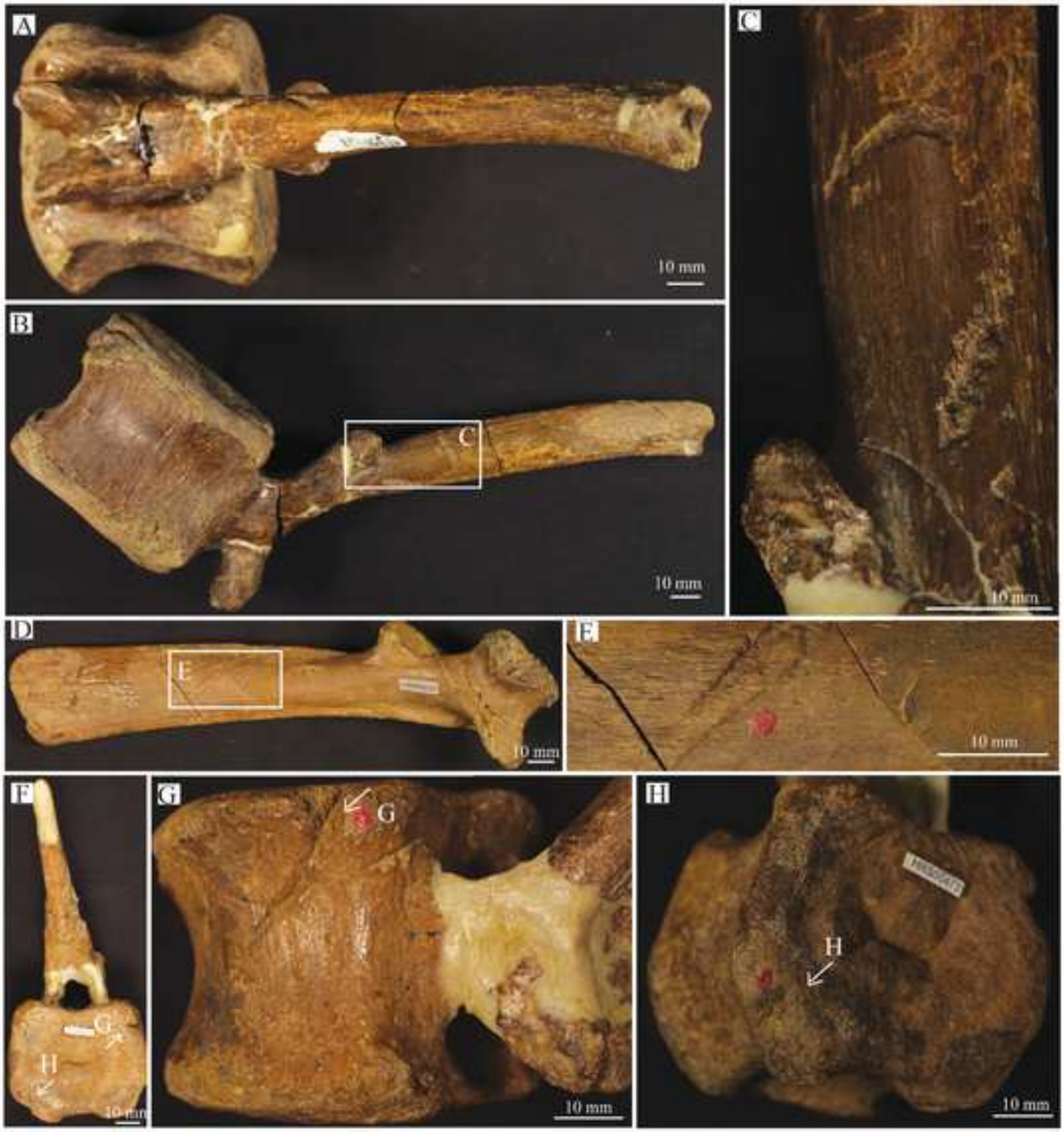

Figure 7

Click here to access/download;Figure;Fig 7.tif

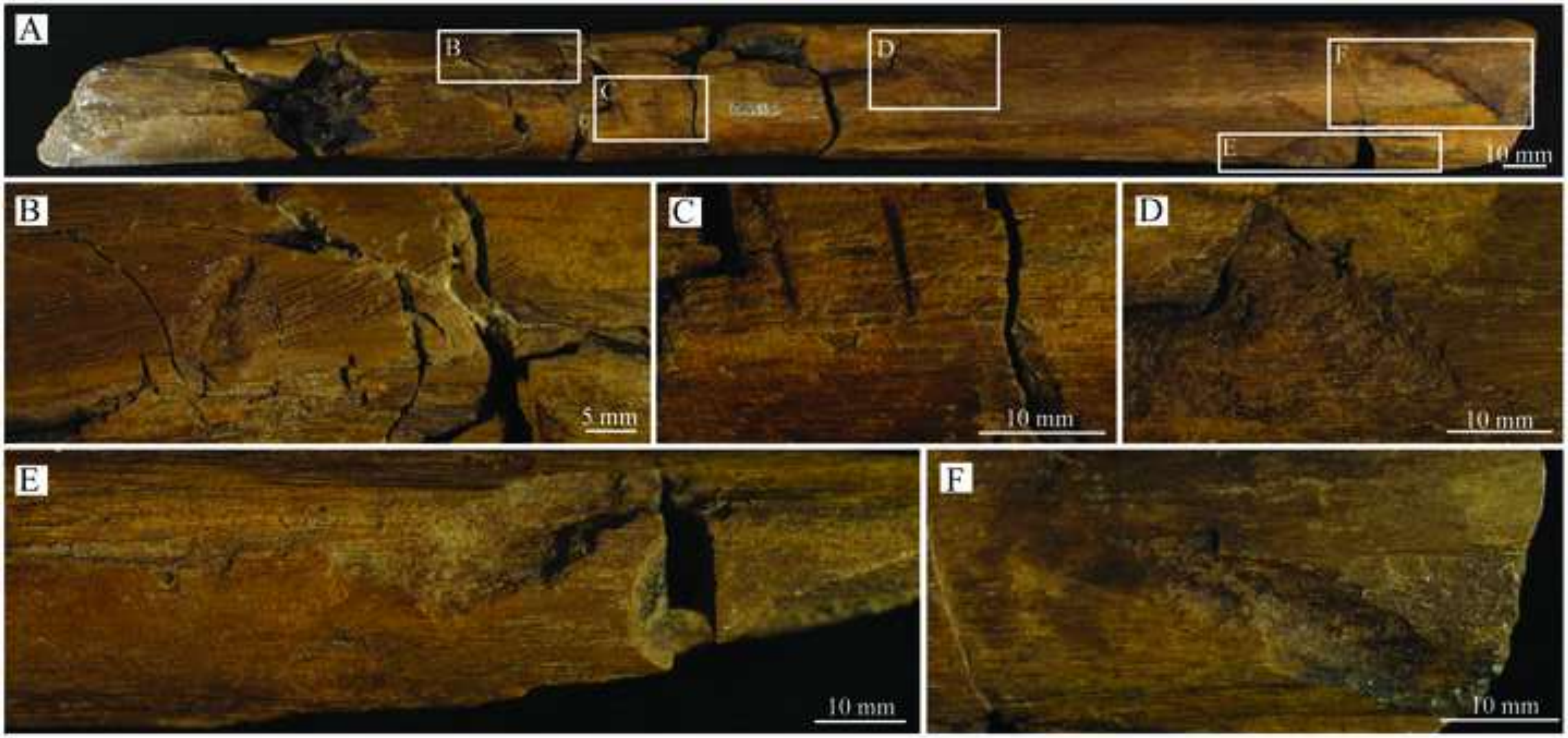

Figure 8

Click here to access/download;Figure;Fig 8.tif

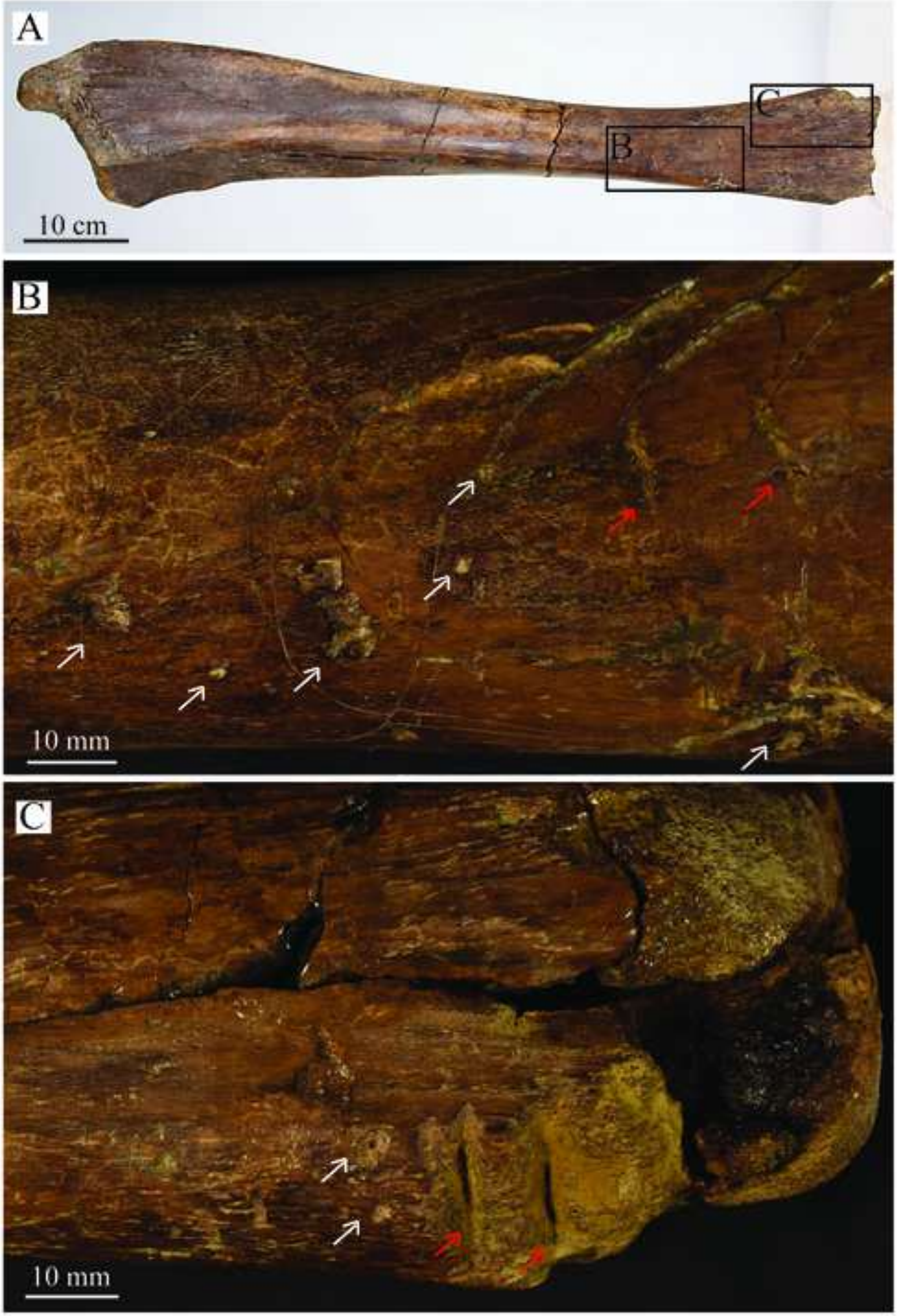

Figure 9

Click here to access/download;Figure;Fig 9.tif

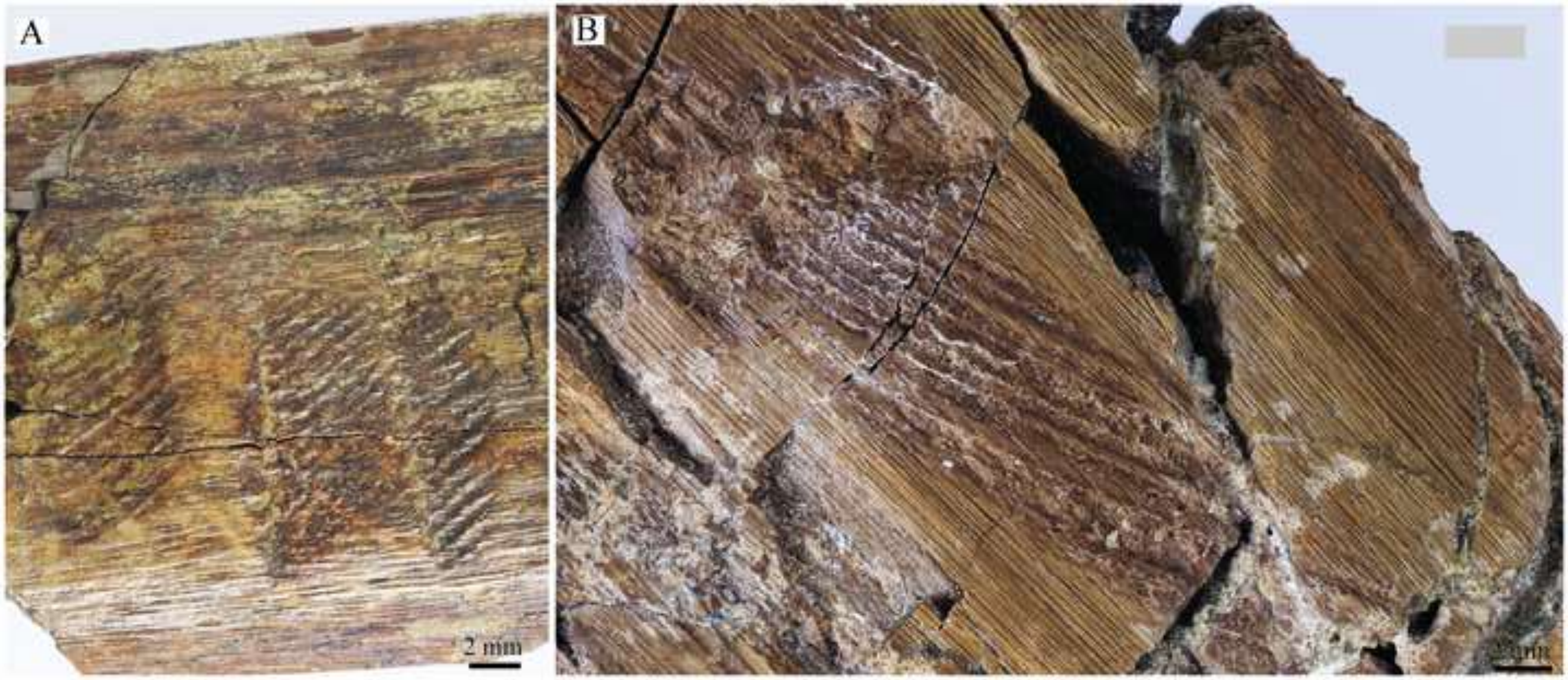

 Click here to access/download;Figure;Fig 10.tif

TOOTH TRACE TYPES				
**Puncture**	**Pit**	**Score**	**Furrow**	**Shearing**
-Circular, oval or polygonal traces<br>-Bowl-shaped cross section<br>-Depth: deep, penetration into cortical bone<br>-Long axis <3X short axis<br>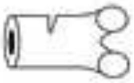	-Circular, oval or polygonal traces<br>-Bowl-shaped cross section<br>-Depth: superficial, no penetration into cortical bone<br>-Long axis < 3X short axis<br>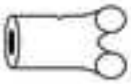	-Linear trace with varied length<br>-U-shaped cross section<br>-Depth: superficial, no penetration into full thickness of cortical bone<br>-Long axis ≥3X short axis<br>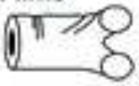	-Linear trace with varied length<br>- U-shaped Cross section<br>-Depth: deep, penetration into full thickness of cortical bone<br>-Long axis ≥3X short axis<br>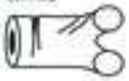	-Removal of a bone piece from a bite while bone still green<br>-Difficult to identify tooth trace<br>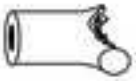

ICHNOTAXA FOR TOOTH TRACE		
Nihilichnus nihilicus	***Linichnus serratus***	***Knethichnus parallelum***
-Punch-hole trace (puncture)<br>-Bowl-shaped cross section<br>-Inflictor: carnivorans, odontocetes, crocodilians, toothed therapod dinosaurs, sharks<br>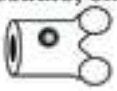	-Curved score trace with serrated outline morphology<br>-Inflictor: ziphodont teeth<br>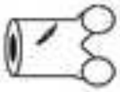	-Series of parallel grooves leading away from initial groove produced by denticles on bone surface<br>-Inflictor: ziphodont teeth<br>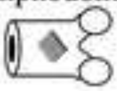

OTHER TYPES OF PERFORATIONS			
**Foramen**	**Bioerosion**	**Pathology**	
-Round anatomical openings on bone surface (often specific to bone)<br>-Penetrate into the bone and normally exit or form a channel system<br>- Perforations with smooth edges and surface<br>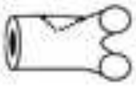	-Round, oval, linear trace of varied depths and lengths<br>-Excavator: worms, bivalves, insects<br>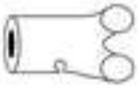	Cloaca<br>-Circular shaped opening<br>-Location: associated to lesion site<br>-Penetrates into bone usually forming channels for pus drainage<br>-Result from infection<br>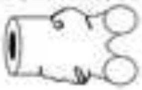	Joint Diseases<br>-Various shaped lesions forming a depression<br>-Depth varies from shallow to deep<br>-Location: joint surfaces<br>-Often associated with bone type<br>-Result from frequent mechanical stress<br>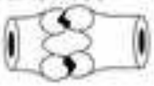

Supporting Information S1_Fig

Click here to access/download
Supporting Information
S1_Fig.docx

Supporting Information S2_Fig

Click here to access/download
Supporting Information
S2_Fig.tif

Supporting Information S3_Fig

Click here to access/download
Supporting Information
S3_ Fig.tif
