# Supplementary material for: Identification of tooth traces from a Cretaceous (Maastrichtian) Edmontosaurus annectens bonebed in the Lance Formation, Wyoming, U.S.A
Source: PLoS One. 2026 Jul 15;21(7):e0351939. doi: 10.1371/journal.pone.0351939 (PMC13372169; doi:10.1371/journal.pone.0351939)
Supplement: Attachment — Submitted filename: Response to Reviwers.docx [file pone.0351939.s008.docx]

**Response to Reviewers**

**REPLIES TO PDF COMMENTS DIRECTLY INTO THE MANUSCRIPT**

A) **PONE-D-2567537_reviewer.pdf:**

- Comment on page 11 pdf file

Page 3 original manuscript

*“most of”*

*Authors’ response: edited according to suggestion (line 82)*

- Comment on page 38 pdf file

Figure 1 in original manuscript

*“I suggest including a map of the United States showing the state of Wyoming, then the actual state of WY and finally the studied area. In the latter include at least one value of latitude and one value of longitude. Also highlight any road or reference in the map. So the final figure will have 3 sub images: United states, WY and the quarry map.”*

*Authors’ response: The authors decided to remove figure one according to another reviewer’s suggestion to remove the original figure 1 (with the map). Instead, we used the reference Synder et al. (2020) since they already discussed and illustrated the same bonebed and quarries location with maps and other figures. Article is published in PLoS One (see reference below).*

*Snyder K, McLain M, Wood J, Chadwick A. Over 13,000 elements from a single bonebed help elucidate disarticulation and transport of an Edmontosaurus thanatocoenosis. PLoS One. 2020 May 21;15(5): e0233182*

B) **PONE-D-2567537-B.Siviero.pdf:**

- Comment on page 11 pdf file

Page 3 original manuscript

*“use a comma instead of semicolon”*

*Authors’ response: edited according to suggestion (revised manuscript with tracking line 83).*

- Comment on page 12 pdf file

Page 4 original manuscript

*“There are many other ichnotaxa that are not footprints and the features mentioned in this sentence. It is better to delete this sentence”*

*Authors’ response: edited according to suggestion. “it is helpful to compare different ichnotaxa to determine perpetrator” (revised manuscript with tracking line 98-99)*

- Comment on page 13 pdf file

Page 5 original manuscript

*“Are there any examples of these in the literature?”*

*Authors’ response: references added into the paragraph according to comment and some text modification (see lines 103-114 in the revised manuscript with tracking). New references were also added into the reference page and order of references by number was modified to accommodate new additional references.*

- Comment on page 13 pdf file

Page 5 original manuscript

“In conclusion,…”

*Authors’ response: edited to “thus” for a better flow since section was edited according to overall suggestions of reviwers (revised manuscript with tracking line 115).*

- Comment on page 14 pdf file

Page 6 original manuscript

“Shouldn't it be Loma Linda University Medical Center?”

*Authors’ response: edited according to suggestion (revised manuscript with tracking line 142).*

- Comment on page 16 pdf file

Page 8 original manuscript

“It's not clear what you mean by this. What context? Are you referring to anatomical, sedimentary or something else? What is shape in this case? What is bone association here?”

*Authors’ response: edited to clarify the statement according to suggestion (revised manuscript with tracking lines 172-174).*

- Comments on pages 17 and 18 pdf file

Pages 9 and 10 original manuscript

“see comment below on the use of verbs in present and past.”

“Some traces are described in present tense (like in Ribs) and others in past tense (like here). It should be consistent throughout the text.”

*Authors’ response: modified according to suggestion to present tense when applicable suggestion (revised manuscript with tracking line 236).*

- Comment on page 22 pdf file

Page 14 original manuscript

“I suppose you want to say Probably trace maker identification by ichnotaxa”

*Authors’ response: edited according to suggestion (revised manuscript with tracking line 322).*

- Comment on page 28 pdf file

Page 20 original manuscript

“This seems strange statement. Right before, you state that you did NOT find any lesions affecting cancellous bone, so, why do you say that you may be underestimating? That possibility undermines your previous bold statement. I don't see the logic here.”

*Authors’ response: edited section to clarify concept based on the comment by reviewer including additional references (revised manuscript with tracking lines 474-476).*

- Comment on page 30 pdf file

Page 22 original manuscript

“shearing?”

*Authors’ response: edited according to suggestion (revised manuscript with tracking line 533).*

C) **PONE-D-2567537TAD.pdf:**

- Comments on page 11 pdf file

Page 3 original manuscript

“Well except the ones referenced above. Also, maybe this is just me, but I think another pass can tighten this section up as it feels a little.....flabby. If you don't agree thats ok, but I just would say maybe do another pass and tighten this a little.” (line 58)

“To be fair they mention the size difference (small theropod vs big a larger pterosaur) and others like Hone et al. 2010 mention the condition of the teeth as also supporting this.” (line 62)

“So this would also include Currie and Jacobsen's data too right? Why not introduce that study in this paragraph or figure a way to combine this paragraph into the earlier one.” (line 64)

*Authors’ response: paragraph was modified and rewritten according to comments and suggestions from this reviewer and other reviewers’ comments below (revised manuscript with tracking lines 56-76).*

- Comments on page 11 pdf file

Page 3 original manuscript

“maybe just indicate which of the above refs you used for this”

*Authors’ response: section of the paragraph was modified to adjust content based on the reviewer’s suggestion and accurately use the reference for the 1^st^ description of tooth traces, and also to accommodate for other reviewers’ comments below (revised manuscript with tracking lines 56-76).*

- Comments on page 12 pdf file

Page 4 original manuscript

“Maybe condense this paragraph as I feel it is a little long and a could use a bit of a trim to make it more punchy.”

*Authors’ response: section of the paragraph was rewritten to accommodate to the best we could the suggestion including suggestions from other reviewers (revised manuscript with tracking lines 56-76).*

- Comments on page 12 pdf file

Page 4 original manuscript

“The second line says this, and I like how it phrases it better. Maybe remove this line.”

*Authors’ response: edited according to suggestion.*

- Comments on page 13 pdf file

Page 5 original manuscript

“Again, combine with paragraph above to make a single, more punchy one that gets the point across.”

*Authors’ response: edited according to suggestion and paragraph was rewritten to also include the suggestions above from page 4 in the original manuscript and comments from other reviewers (revised manuscript with tracking lines 100-117).*

- Comments on page 15 pdf file

Page 7 original manuscript

“maybe not predator, but just call it a trace maker as if it is scavenging. Especially as the term "predator" has ecological implications, maybe not use that unless you are really confident it was a predation event by that trace maker”

*Authors’ response: agree with comment. Sentence was modified to reflect the authors’ intention (revised manuscript with tracking lines 147-148).*

- Comments on page 16 pdf file

Page 8 original manuscript

“Maybe put this section after the section dealing with more confidently asserted tooth traces?”

*Authors’ response: the authors decided to initially discuss what was not a tooth trace (from our selected fossils) and then focus on the fossils that we determined to represent actual tooth traces based on our experience on the more questionable ones.*

- Comments on page 20 pdf file

Page 12 original manuscript

“move this up, so when these taxa are referenced later there is context for the reader.”

*Authors’ response: thank you for the suggestion.* *Authors decided to maintain the original order. We believe that first describing each specimen by its morphology and, when applicable, its potential ichnotaxa, was better since the reader has already been introduced to ichnotaxa in the introduction. In a later section in the results, we prefer to focus on a few selected specimens that actually have preserve tooth-trace ichnotaxa to finalize results on potential tooth trace maker. See also* **Reply to reviewer #5 comments** *below.*

**REPLIES TO ADDITIONAL COMMENTS BY REVIEWERS**

D) **Reply to reviewer #1 comments:**

(Comments by reviewer in black)

“The manuscript is very well written, with clear explanations and well-illustrated. The limitations of interpretation are well considered and explained. Figure 10 and S1 Fig seem to be the same. You should move S2 Fig and S3 Fig into the main text rather than the Supplementary section.”

*Authors’ response: Figure S1 in the original manuscript is supplemental review of tooth traces classification according to literature review. Figure 10 in the original manuscript (revised manuscript is now Figure 9) includes every lesion that resembles a tooth trace. It incorporates information from figure S1* ***and*** *additional lesions/depressions to take into consideration when identifying or classifying tooth traces. Authors decided to maintain the supplemental material figure S1 as in the original manuscript since the is just a visual review of the described literature review. Figures S2 and S3 were also maintained in the supplemental material since they are an indirect part of this article and are just examples for the content discussed but still necessary to be in supplemental material since it adds to the interpretation.*

E) **Reply to reviewer #2 comments:**

(Comments by reviewer in black)

“The study presents results that appear to be original and not previously published, and the manuscript does not show evidence of direct duplication of figures or text from earlier peer-reviewed publications. The research question is incremental rather than groundbreaking, but this is acceptable under PLOS ONE’s scope.
However, the manuscript would benefit from a clearer positioning relative to closely related prior studies, particularly to better articulate what is novel about the present contribution beyond the presentation of tooth traces. The Abstract Introduction should more explicitly state how this work advances existing knowledge rather than reiterating established patterns. For example, only at the end of the mauscript it is cited the guide for the identification of tooth traces
488 (Fig 10) to incorporate many different types of bone perforations. In my opinion this is the most relevant part of the MS, since is a true contribution that enhances previous guideliness (showed in Fig S1).
So, only at the end of the discussion I realized that the MS is adding value other than just a description of tooth bites on some dinosaurs.
I think that of the MS is intended to be published in PLOS ONE, it needs to add value somehow, not only presenting a bit marks in a descriptive way.
I suggest re-shaping the MS focusing on the guide of tooth marks. As I see, the guide is the product of this MS.”

*Authors’ response: the original order in the manuscript is how we refined the criteria. We started to look for tooth traces according to the descriptions and soon we realized that context is important and that other tooth trace like perforations are also possible and could be misidentified. This is discussed and presented in order in the manuscript since the refined criteria are the final product of this study. However, additional sentences and edits were included according to suggestions to emphasize the revision of the criteria for tooth traces in the abstract and introduction (revised manuscript with tracking lines 48-52, 100-125) to bring to attention our contribution in not only describing the traces but also in enhancing the guidelines to tooth traces.*

“Coupled with this, the figures need to be of better quality (although maybe they lost quality in the PDF, but please, check the original files since they were not provided) and clearly show the mark patterns matched with the guide.”

*Authors’ response: PDF version lost definition as indicated by the reviewers.*

*Figures were submitted according to the guidelines of the journal.*

“See the commented PDF for few other comments.”

*Authors’ response: Thank you for the suggestion. All comments associated with PDF were addressed above under section “Replies to PDF Comments Directly into the Manuscript”.*

F) **Reply to reviewer #3 comments:**

(Comments by reviewer in black)

“The manuscript constitutes an interesting contribution to understanding certain biological features commonly found in fossil vertebrates. I believe this manuscript does not require major changes.
My only comment regards a sentence in page 14 related to the possible recognition of Nanotyrannus as a juvenile Tyrannosaurus rex. This has been recently discussed by other authors that sufficiently support they are different taxa (see Zanno, L. E., & Napoli, J. G. (2025). Nanotyrannus and Tyrannosaurus coexisted at the close of the Cretaceous. Nature, 1-3). Perhaps this can be relevant to the identification of the tooth marks inflictor.”

*Authors’ response: Thank you for the suggestion. Text was edited to reflect the suggestion with in-text references revised. Reference section of the manuscript was also adjusted to reflect these changes (revised manuscript with tracking lines 326-327).*

G) **Reply to reviewer #4 comments:**

(Comments by reviewer in black)

“The submitted MS reports the results of investigation of tooth traces of Edmontosaurus bones from Lance Formation. This topic is more than welcome, because numerous articles have been published in recent years on the examination of bite marks, which in many cases can be used to draw palaeoecological conclusions. However, unfortunately, this manuscript does not contain any new relevant scientific information related to the examination of bite marks, but only uses previously published observations in the examination of their own material. I am not saying that there is no need to mention the examples cited in the manuscript in a publication, but I just wanted to emphasize that the current manuscript does not contain any significant new findings. The conclusion chapter also contains entirely general statements. If the authors disagree with this, it would be justified to formulate the new results listed in the conclusions more precisely, and it would be worthwhile to emphasize in the main text of the article what different or new results have been achieved in the field of bite mark examination. In summary, the article is descriptive in nature. It contains observations, but does not place them in a broader context. However, the results presented in the study may be used as data in other publications, so the editor will decide whether to publish the study.

*Authors’ response: Thank you for the comments. Several sections of this manuscript were edited according to suggestions. Discussion and Conclusion sections of the manuscript we edited and added content according to the suggestions.*

• There are numerous great scientific publications dealing with bite marks of Mesozoic bones, but only a few studies are mentioned in the publication, which is often based on a rather arbitrary selection. I believe that the authors do not refer to fundamental works published on the subject (e.g. Fiorill, 1991; Carpenter, 1998; Jacobsen, 1998; Farlow and Holtz, 2002; Avilla et al., 2004; Buffetaut et al., 2004; Hone and Rauhut, 2009; Fiorelli, 2010; Longrich et al., 2010; Milàn et. al., 2010; Schwimmer, 2010; Bell, et al., 2012; Boyd et al., 2013, Botfalvai et al 2014 and references therein). I recommend taking a critical look at the publication mentioned and the references listed.

*Authors’ response: Thank you for the recommendation. We have incorporated several of the recommended publications where relevant to illustrate tooth traces and animal interactions within the introduction section of this manuscript. As this is not a review paper on tooth traces and animal interactions, we have not expanded these sections extensively but have included selected citations where appropriate to strengthen our introduction. Some of these references were also added into the discussion section of the manuscript, when applicable to the interpretations of tooth trace inflictors. See revised manuscript with tracking lines 56-75,349-369,490). References were also included in the reference section.*

• Surangular fossil HRS05499: The authors analyse the perforations/lesions visible on this fossil in a separate section. In the end, they conclude that these are not bite marks, but are normal foramina found in a triceratopsini surangular. The figure used as evidence (Figure 3) is of such poor resolution that I could not make out anything on it. It is possible that this is a mistake of the journal and only the generated PDF file contains such a poor quality figure. In any case, based on the available material, I cannot judge whether the authors are right on this issue. Comparing the marks on the angular bone of HRS05499 with those on other triceratopsine angular bones would help clarify this issue. Furthermore, it is necessary to list other anatomical features that link this bone element to triceratopsine dinosaurs. Are there other bones from the site that originate from ceratopsian dinosaurs?

*Authors’ response: Regarding the quality of the figures PDF version lost definition as indicated by other reviewers. Figures were submitted according to the guidelines of the journal and in the PDF version it might have lost quality. Regarding HRS05499 and other ceratopsian bones, yes, ceratopsian fossil bones are occasionally found in different quarries in this study site (from multiple bonebeds) and within the main bonebed quarries (specifically associated with this manuscript). We compared the surangular HRS05599 with other ceratopsian dinosaur surangular bones from other museum collections (indicated in the results and discussion sections of the manuscript). We also rewrote the section of the discussion (revised manuscript lines 415-421) to clarify the content according to the reviewer’s comment (and other reviewers’ comments).*

• The authors discuss at length that bite marks can often be confused with anatomically present foramina. I don't really understand why this needs to be emphasized. A detailed examination of the bite marks begins with determining the taxonomic classification of the bone being examined. Bones that cannot be classified taxonomically cannot be examined for bite marks, as in such cases we do not know what foramina the original bone contained. In my opinion, this is a conclusion that everyone knows even without the article.

*Authors’ response: in the original manuscript (word file lines 376-379), discussion section, we mentioned that “If one is not familiar with the specific osteological features in a specimen, especially in extinct species, these perforations could potentially be mistakenly identified as a tooth trace” and in the revised manuscript (revision with tracking line 408) we added, “especially for the untrained eye”. In our investigation to find out the etiology of the perforations on the surangular, we found that this could be potential for misinterpretation, even after talking with various paleontologists. Thus, we found that this concept was also important to include in our findings and discussions.*

“Figures:
• I realize that this is not the authors' fault, but the PDF figures sent by the journal are of very poor resolution. Therefore, in many cases, I was unable to verify the evidence that the authors wrote about in the text. So, at the moment, I can only hope that the authors have created images with much better resolution, where everything is much clearer. The following figures were the most problematic in this regard: Figure 2B-C; Figure 3; Figure 4A;
• Figure 1: For me, this is not a very informative figure. I know that it is not a very important figure in this study, but I still felt I should point out that it does not provide any information about the site. A sedimentary section, a photograph of the bone-bearing beds, an excavation picture, etc. would improve this figure and enhance the appearance of the article.”

*Authors’ response: Thank you for the suggestions. Regarding the quality of the figures PDF version lost definition as indicated by other reviewers. Figures were submitted according to the guidelines of the journal and in the PDF version it might have lost quality. Regarding Figure 1 in the original manuscript, authors deleted the image since it was not essential and previous publication (cited in the text where the location was mentioned in the edited version) by Snyder at al. 2020 reports the location with maps and images of the different quarries and stratigraphic context. This modification and reference in text about the location of the site should adequately address the reviewer’s comment.*

*Snyder K, McLain M, Wood J, Chadwick A. Over 13,000 elements from a single bonebed help elucidate disarticulation and transport of an Edmontosaurus thanatocoenosis. PLoS One. 2020 May 21;15(5): e0233182.*

H) **Reply to reviewer #5 comments:**

(Comments by reviewer in black)

“Overall I like this manuscript and it's attempt to more clearly define and confine what is a tooth trace on a fossil, as these have important ecological and behavioural implications. The main issue I have, and it's not a major one, is the writing style and organization of this manuscript could be improved to increase readability (especially for a more general audience). I suggest another pass through to tighten and moving around results sections would make it flow better.

But overall a god manuscript and I look forward to seeing it published without much alteration of the core of the text.”

*Authors’ response: Thank you for the comment. The authors made edits in the text and reorganized parts of this manuscript. We hope these changes will help the reader. Based on another comment (***PONE-D-2567537TAD.pdf)***above regarding the organization in the results for the ichnotaxa for tooth traces, we decided to maintain first the description of the bones to then follow with the ichnotaxa as we believe that is first necessary to describe the perforation (and give names to ichnotaxa for tooth traces when applicable) and then interpret. The background about the ichnotaxa for tooth traces is first given in the introduction thus by the results the reader should be able to understand the names given for the ichnotaxa based on their criteria.*

I) **Reply to reviewer #6 comments:**

(Comments by reviewer in black)

“This manuscript by Siviero and colleagues provides a rigorous and appropriately cautious approach to identifying the organisms behind tooth traces in a dinosaur bonebed from the Late Cretaceous. The research is well documented, with sufficient information to follow and reproduce the analysis in other settings. Similarly, I feel that the assignment of tooth marks to Tyrannosaurus is well supported with the evidence presented. A number of minor points are outlined below, but I wish to call attention to three in particular:

1) Determining the etiology of marks on bone has a significant body of research in paleoanthropology and archaeology, particularly in distinguishing human-created tool marks from other biotic and abiotic causes. I would suggest incorporating some of this research here, because it is directly relevant. This includes wear marks from trampling or soil compaction (e.g., Behrensmeyer et al., 1986; Dominguez-Rodrigo et a., 2009; Fiorillo, 1989; Olsen and Shipman, 1988; Rozada et al., 2018).

*Authors’ response: Thank you for the suggestion. We rewrote the paragraph in the discussion sections of the manuscript (revised manuscript with tracking lines 513-526) to further discuss the topic of these other different bone surface modifications and included the suggested references above in text and in the reference sections of the manuscript.*

2) Similarly, a few additional paleontological papers on tooth marks related to crocodilian and archosaur feeding, etc., could be cited. This includes Drumheller et a., 2023; Njau and Gilber, 2016; Erickson and Olson, 1996; and citations therein.

*Authors’ response: Thank you for the suggestion. We added additional references according to suggestions in the introduction section of this manuscript (revised manuscript with tracking lines 56-76) and we also created a new paragraph to include more on the discussion of the tooth traces including the suggested papers above and added a section in the conclusion to accommodate for the importance of these references (revised manuscript with tracking lines 349-369, 544-552) and additional papers. We also added the references into the reference section of the manuscript.*

3) The ultimate identification of HRS05499 is ambiguous. Can you definitively say it is ceratopsid based on other aspects of morphology? Or is it a weird Edmontosaurus that has a ceratopsid-like foramen pattern? Ceratopsid and hadrosaurid surangulars should be fairly distinct in overall shape (not just the pattern of foramina), so I think this should be addressed in more detail. It is not clear from the text as currently written.
*Authors’ response: we edited some of the text in the result section of the manuscript and in the discussion to clarify the concept according to suggestion. See revised manuscript with tracking lines 187-194, 415-421).*

Minor Comments:
- The recent Siviero et al. 2025 reference may now be added, because it includes some of the same sample.

*Authors’ response: Added according to the suggestion in several locations of the manuscript when necessary.*

- lines 91-92: Are the inferences around Knethichnus and Linichnus related to the same citation as earlier in the paragram? I would add the citation here again, if approriate, because it is fairly well separated in the text.

*Authors’ response: Added according to the suggestion.*

- line 180: Can the teeth be referred to Borealosuchus definitively? Tooth morphology is pretty flexible in crocs, so I personally prefer to be a bit more cautious - e.g., "Eusuchia indet., likely from Borealosuchus sternbergii". They're likely not Brachychampsa, of course!

*Authors’ response: Thank you for the suggestion. We have revised the text according to the suggestion. The crocodilian teeth occasionally lost within the bone bed lack serrations and are strongly conical in shape. Accordingly, we now describe them using more general terminology since we cannot be definitively certain if Borealosuchus sternbergii (revised manuscript with tracking lines 179-180).*

- line 323: You might add the newest Nanotyrannus papers here that more definitively settle the issue

*Authors’ response: we added additional reference according to suggestion and from another reviewer above. See revised manuscript with tracking lines 326-327.*

- Erickson and Olson, 1996, discusses other bite marks referable to Tyrannosaurus rex; this should be cited.

*Authors’ response: Thank you for the suggestion. I have cited and added this reference and others as suggested above.*

**ADDITIONAL REMARKS BY AUTHORS**

1. *We added the sentence “No permits were required for the described study, which complied with all relevant regulations” under the materials and methods section of the manuscript (revised manuscript with tracking lines 144-145).*
2. *Based on the suggestions, comments, and additional references, we have reorganized the numbering for the figures, in-text citation order and associated “References” section of this manuscript to accommodate these changes.*
3. *We also fixed and tracked some minor grammar mistakes.*
4. *We edited some of the formatting to meet journal’s style requirements.*
