# Supplementary material for: Identification of tooth traces from a Cretaceous (Maastrichtian) Edmontosaurus annectens bonebed in the Lance Formation, Wyoming, U.S.A
Source: PLoS One. 2026 Jul 15;21(7):e0351939. doi: 10.1371/journal.pone.0351939 (PMC13372169; doi:10.1371/journal.pone.0351939)
Supplement: Peer review history

# Peer review: Decision Letter 0

**DOI:** 10.1371/journal.pone.0351939.r001

**Authors:** Furqan A. Shah

4 Feb 2026

PONE-D-25-67537

Identification of tooth traces from a Cretaceous (Maastrichtian) Edmontosaurus annectens bonebed in the Lance Formation, Wyoming, U.S.A.

PLOS One

Dear Dr. C. T. Siviero,

Thank you for submitting your manuscript to PLOS ONE. After careful consideration, we feel that it has merit but does not fully meet PLOS ONE’s publication criteria as it currently stands. Therefore, we invite you to submit a revised version of the manuscript that addresses the points raised during the review process.

Please submit your revised manuscript by Mar 21 2026 11:59PM. If you will need more time than this to complete your revisions, please reply to this message or contact the journal office at plosone@plos.org. When you're ready to submit your revision, log on to https://www.editorialmanager.com/pone/ and select the 'Submissions Needing Revision' folder to locate your manuscript file.

Please include the following items when submitting your revised manuscript:

- A letter that responds to each point raised by the academic editor and reviewer(s). You should upload this letter as a separate file labeled 'Response to Reviewers'.
- A marked-up copy of your manuscript that highlights changes made to the original version. You should upload this as a separate file labeled 'Revised Manuscript with Track Changes'.
- An unmarked version of your revised paper without tracked changes. You should upload this as a separate file labeled 'Manuscript'.

If you would like to make changes to your financial disclosure, please include your updated statement in your cover letter. Guidelines for resubmitting your figure files are available below the reviewer comments at the end of this letter.

If applicable, we recommend that you deposit your laboratory protocols in protocols.io to enhance the reproducibility of your results. Protocols.io assigns your protocol its own identifier (DOI) so that it can be cited independently in the future. For instructions see: https://journals.plos.org/plosone/s/submission-guidelines#loc-laboratory-protocols. Additionally, PLOS ONE offers an option for publishing peer-reviewed Lab Protocol articles, which describe protocols hosted on protocols.io. Read more information on sharing protocols at https://plos.org/protocols?utm_medium=editorial-email&utm_source=authorletters&utm_campaign=protocols.

We look forward to receiving your revised manuscript.

Kind regards,

Furqan A. Shah

Academic Editor

PLOS One

Journal requirements:

When submitting your revision, we need you to address these additional requirements.

1. Please ensure that your manuscript meets PLOS ONE's style requirements, including those for file naming. The PLOS ONE style templates can be found at

https://journals.plos.org/plosone/s/file?id=wjVg/PLOSOne_formatting_sample_main_body.pdf and

https://journals.plos.org/plosone/s/file?id=ba62/PLOSOne_formatting_sample_title_authors_affiliations.pdf

2. In your manuscript, please provide additional information regarding the specimens used in your study. Ensure that you have reported human remain specimen numbers and complete repository information, including museum name and geographic location.

If permits were required, please ensure that you have provided details for all permits that were obtained, including the full name of the issuing authority, and add the following statement:

'All necessary permits were obtained for the described study, which complied with all relevant regulations.'

If no permits were required, please include the following statement:

'No permits were required for the described study, which complied with all relevant regulations.'

For more information on PLOS One's requirements for paleontology and archeology research, see https://journals.plos.org/plosone/s/submission-guidelines#loc-paleontology-and-archaeology-research.

3. We note that Figure 1 in your submission contain [map/satellite] images which may be copyrighted. All PLOS content is published under the Creative Commons Attribution License (CC BY 4.0), which means that the manuscript, images, and Supporting Information files will be freely available online, and any third party is permitted to access, download, copy, distribute, and use these materials in any way, even commercially, with proper attribution. For these reasons, we cannot publish previously copyrighted maps or satellite images created using proprietary data, such as Google software (Google Maps, Street View, and Earth). For more information, see our copyright guidelines: http://journals.plos.org/plosone/s/licenses-and-copyright.

We require you to either (1) present written permission from the copyright holder to publish these figures specifically under the CC BY 4.0 license, or (2) remove the figures from your submission:

1. You may seek permission from the original copyright holder of Figure 1 to publish the content specifically under the CC BY 4.0 license.

We recommend that you contact the original copyright holder with the Content Permission Form (http://journals.plos.org/plosone/s/file?id=7c09/content-permission-form.pdf) and the following text:

“I request permission for the open-access journal PLOS ONE to publish XXX under the Creative Commons Attribution License (CCAL) CC BY 4.0 (http://creativecommons.org/licenses/by/4.0/). Please be aware that this license allows unrestricted use and distribution, even commercially, by third parties. Please reply and provide explicit written permission to publish XXX under a CC BY license and complete the attached form.”

Please upload the completed Content Permission Form or other proof of granted permissions as an "Other" file with your submission.

In the figure caption of the copyrighted figure, please include the following text: “Reprinted from [ref] under a CC BY license, with permission from [name of publisher], original copyright [original copyright year].”

2. If you are unable to obtain permission from the original copyright holder to publish these figures under the CC BY 4.0 license or if the copyright holder’s requirements are incompatible with the CC BY 4.0 license, please either i) remove the figure or ii) supply a replacement figure that complies with the CC BY 4.0 license. Please check copyright information on all replacement figures and update the figure caption with source information. If applicable, please specify in the figure caption text when a figure is similar but not identical to the original image and is therefore for illustrative purposes only.

The following resources for replacing copyrighted map figures may be helpful:

USGS National Map Viewer (public domain): http://viewer.nationalmap.gov/viewer/

The Gateway to Astronaut Photography of Earth (public domain): http://eol.jsc.nasa.gov/sseop/clickmap/

Maps at the CIA (public domain): https://www.cia.gov/library/publications/the-world-factbook/index.html and https://www.cia.gov/library/publications/cia-maps-publications/index.html

NASA Earth Observatory (public domain): http://earthobservatory.nasa.gov/

Landsat: http://landsat.visibleearth.nasa.gov/

USGS EROS (Earth Resources Observatory and Science (EROS) Center) (public domain): http://eros.usgs.gov/#

Natural Earth (public domain): http://www.naturalearthdata.com/

If the reviewer comments include a recommendation to cite specific previously published works, please review and evaluate these publications to determine whether they are relevant and should be cited. There is no requirement to cite these works unless the editor has indicated otherwise.

[Note: HTML markup is below. Please do not edit.]

Reviewers' comments:

Reviewer's Responses to Questions

**Comments to the Author**

1. Is the manuscript technically sound, and do the data support the conclusions?

The manuscript must describe a technically sound piece of scientific research with data that supports the conclusions. Experiments must have been conducted rigorously, with appropriate controls, replication, and sample sizes. The conclusions must be drawn appropriately based on the data presented.

Reviewer #1: Yes

Reviewer #2: Partly

Reviewer #3: Yes

Reviewer #4: Partly

Reviewer #5: Yes

Reviewer #6: Yes

**********

2. Has the statistical analysis been performed appropriately and rigorously?

Reviewer #1: N/A

Reviewer #2: N/A

Reviewer #3: Yes

Reviewer #4: N/A

Reviewer #5: Yes

Reviewer #6: N/A

**********

3. Have the authors made all data underlying the findings in their manuscript fully available?

The PLOS Data policy requires authors to make all data underlying the findings described in their manuscript fully available without restriction, with rare exception (please refer to the Data Availability Statement in the manuscript PDF file). The data should be provided as part of the manuscript or its supporting information, or deposited to a public repository. For example, in addition to summary statistics, the data points behind means, medians and variance measures should be available. If there are restrictions on publicly sharing data—e.g. participant privacy or use of data from a third party—those must be specified.

Reviewer #1: Yes

Reviewer #2: Yes

Reviewer #3: Yes

Reviewer #4: Yes

Reviewer #5: Yes

Reviewer #6: Yes

**********

4. Is the manuscript presented in an intelligible fashion and written in standard English?

PLOS ONE does not copyedit accepted manuscripts, so the language in submitted articles must be clear, correct, and unambiguous. Any typographical or grammatical errors should be corrected at revision, so please note any specific errors here.

Reviewer #1: Yes

Reviewer #2: Yes

Reviewer #3: Yes

Reviewer #4: Yes

Reviewer #5: Yes

Reviewer #6: Yes

**********

5. Review Comments to the Author

Please use the space provided to explain your answers to the questions above. You may also include additional comments for the author, including concerns about dual publication, research ethics, or publication ethics. (Please upload your review as an attachment if it exceeds 20,000 characters)

Reviewer #1: The manuscript is very well written, with clear explanations and well-illustrated. The limitations of interpretation are well considered and explained. Figure 10 and S1 Fig seem to be the same. You should move S2 Fig and S3 Fig into the main text rather than the Supplementary section.

Reviewer #2: The study presents results that appear to be original and not previously published, and the manuscript does not show evidence of direct duplication of figures or text from earlier peer-reviewed publications. The research question is incremental rather than groundbreaking, but this is acceptable under PLOS ONE’s scope.

However, the manuscript would benefit from a clearer positioning relative to closely related prior studies, particularly to better articulate what is novel about the present contribution beyond the presentation of tooth traces. The Abstract Introduction should more explicitly state how this work advances existing knowledge rather than reiterating established patterns. For example, only at the end of the mauscript it is cited the guide for the identification of tooth traces

488 (Fig 10) to incorporate many different types of bone perforations. In my opinion this is the most relevant part of the MS, since is a true contribution that enhances previous guideliness (showed in Fig S1).

So, only at the end of the discussion I realized that the MS is adding value other than just a description of tooth bites on some dinosaurs.

I think that of the MS is intended to be published in PLOS ONE, it needs to add value somehow, not only presenting a bit marks in a descriptive way.

I suggest re-shaping the MS focusing on the guide of tooth marks. As I see, the guide is the product of this MS.

Coupled with this, the figures need to be of better quality (although maybe they lost quality in the PDF, but please, check the original files since they were not provided) and clearly show the mark patterns matched with the guide.

See the commented PDF for few other comments.

All the best.

Reviewer #3: The manuscript constitutes an interesting contribution to understanding certain biological features commonly found in fossil vertebrates. I believe this manuscript does not require major changes.

My only comment regards a sentence in page 14 related to the possible recognition of Nanotyrannus as a juvenile Tyrannosaurus rex. This has been recently discussed by other authors that sufficiently support they are different taxa (see Zanno, L. E., & Napoli, J. G. (2025). Nanotyrannus and Tyrannosaurus coexisted at the close of the Cretaceous. Nature, 1-3). Perhaps this can be relevant to the identification of the tooth marks inflictor.

Reviewer #4: The submitted MS reports the results of investigation of tooth traces of Edmontosaurus bones from Lance Formation. This topic is more than welcome, because numerous articles have been published in recent years on the examination of bite marks, which in many cases can be used to draw palaeoecological conclusions. However, unfortunately, this manuscript does not contain any new relevant scientific information related to the examination of bite marks, but only uses previously published observations in the examination of their own material. I am not saying that there is no need to mention the examples cited in the manuscript in a publication, but I just wanted to emphasize that the current manuscript does not contain any significant new findings. The conclusion chapter also contains entirely general statements. If the authors disagree with this, it would be justified to formulate the new results listed in the conclusions more precisely, and it would be worthwhile to emphasize in the main text of the article what different or new results have been achieved in the field of bite mark examination. In summary, the article is descriptive in nature. It contains observations, but does not place them in a broader context. However, the results presented in the study may be used as data in other publications, so the editor will decide whether to publish the study.

• There are numerous great scientific publications dealing with bite marks of Mesozoic bones, but only a few studies are mentioned in the publication, which is often based on a rather arbitrary selection. I believe that the authors do not refer to fundamental works published on the subject (e.g. Fiorill, 1991; Carpenter, 1998; Jacobsen, 1998; Farlow and Holtz, 2002; Avilla et al., 2004; Buffetaut et al., 2004; Hone and Rauhut, 2009; Fiorelli, 2010; Longrich et al., 2010; Milàn et. al., 2010; Schwimmer, 2010; Bell, et al., 2012; Boyd et al., 2013, Botfalvai et al 2014 and references therein). I recommend taking a critical look at the publication mentioned and the references listed.

• Surangular fossil HRS05499: The authors analyse the perforations/lesions visible on this fossil in a separate section. In the end, they conclude that these are not bite marks, but are normal foramina found in a triceratopsini surangular. The figure used as evidence (Figure 3) is of such poor resolution that I could not make out anything on it. It is possible that this is a mistake of the journal and only the generated PDF file contains such a poor quality figure. In any case, based on the available material, I cannot judge whether the authors are right on this issue. Comparing the marks on the angular bone of HRS05499 with those on other triceratopsine angular bones would help clarify this issue. Furthermore, it is necessary to list other anatomical features that link this bone element to triceratopsine dinosaurs. Are there other bones from the site that originate from ceratopsian dinosaurs?

• The authors discuss at length that bite marks can often be confused with anatomically present foramina. I don't really understand why this needs to be emphasized. A detailed examination of the bite marks begins with determining the taxonomic classification of the bone being examined. Bones that cannot be classified taxonomically cannot be examined for bite marks, as in such cases we do not know what foramina the original bone contained. In my opinion, this is a conclusion that everyone knows even without the article.

Figures:

• I realize that this is not the authors' fault, but the PDF figures sent by the journal are of very poor resolution. Therefore, in many cases, I was unable to verify the evidence that the authors wrote about in the text. So, at the moment, I can only hope that the authors have created images with much better resolution, where everything is much clearer. The following figures were the most problematic in this regard: Figure 2B-C; Figure 3; Figure 4A;

• Figure 1: For me, this is not a very informative figure. I know that it is not a very important figure in this study, but I still felt I should point out that it does not provide any information about the site. A sedimentary section, a photograph of the bone-bearing beds, an excavation picture, etc. would improve this figure and enhance the appearance of the article.

Reviewer #5: Overall I like this manuscript and it's attempt to more clearly define and confine what is a tooth trace on a fossil, as these have important ecological and behavioural implications. The main issue I have, and it's not a major one, is the writing style and organization of this manuscript could be improved to increase readability (especially for a more general audience). I suggest another pass through to tighten and moving around results sections would make it flow better.

But overall a god manuscript and I look forward to seeing it published without much alteration of the core of the text.

Reviewer #6: This manuscript by Siviero and colleagues provides a rigorous and appropriately cautious approach to identifying the organisms behind tooth traces in a dinosaur bonebed from the Late Cretaceous. The research is well documented, with sufficient information to follow and reproduce the analysis in other settings. Similarly, I feel that the assignment of tooth marks to Tyrannosaurus is well supported with the evidence presented. A number of minor points are outlined below, but I wish to call attention to three in particular:

1) Determining the etiology of marks on bone has a significant body of research in paleoanthropology and archaeology, particularly in distinguishing human-created tool marks from other biotic and abiotic causes. I would suggest incorporating some of this research here, because it is directly relevant. This includes wear marks from trampling or soil compaction (e.g., Behrensmeyer et al., 1986; Dominguez-Rodrigo et a., 2009; Fiorillo, 1989; Olsen and Shipman, 1988; Rozada et al., 2018).

2) Similarly, a few additional paleontological papers on tooth marks related to crocodilian and archosaur feeding, etc., could be cited. This includes Drumheller et a., 2023; Njau and Gilber, 2016; Erickson and Olson, 1996; and citations therein.

3) The ultimate identification of HRS05499 is ambiguous. Can you definitively say it is ceratopsid based on other aspects of morphology? Or is it a weird Edmontosaurus that has a ceratopsid-like foramen pattern? Ceratopsid and hadrosaurid surangulars should be fairly distinct in overall shape (not just the pattern of foramina), so I think this should be addressed in more detail. It is not clear from the text as currently written.

Minor Comments:

- The recent Siviero et al. 2025 reference may now be added, because it includes some of the same sample.

- lines 91-92: Are the inferences around Knethichnus and Linichnus related to the same citation as earlier in the paragram? I would add the citation here again, if approriate, because it is fairly well separated in the text.

- line 180: Can the teeth be referred to Borealosuchus definitively? Tooth morphology is pretty flexible in crocs, so I personally prefer to be a bit more cautious - e.g., "Eusuchia indet., likely from Borealosuchus sternbergii". They're likely not Brachychampsa, of course!

- line 323: You might add the newest Nanotyrannus papers here that more definitively settle the issue

- Erickson and Olson, 1996, discusses other bite marks referable to Tyrannosaurus rex; this should be cited.

Behrensmeyer, A.K., Gordon, K.D., and Yanagi, G.T. 1986. Trampling as a cause of bone surface damage and pseudo-cutmarks. Nature, 319:768-771.

https://doi.org/10.1038/319768a0

Dominguez-Rodrigo, M., S. de Juana, A.B. Galan, and M. Rodriguez, 2009. A new protocol to differentiate trampling marks from butchery cut marks. Journal of Archaeological Science, 1-12.

Drumheller, S. K., D’Amore, D. C., Njau, J. K. 2023. Taphonomic approaches to bite-mark analyses in the fossil record and applications to archosaurian paleobiology. In IUP Crocodylian Paleobiology.

Erickson, G. M., & Olson, K. H. (1996). Bite marks attributable to Tyrannosaurus rex: preliminary description and implications. Journal of Vertebrate Paleontology, 16(1), 175-178.

Fiorillo, A.R., 1989. An experimental study of trampling: implications for the fossil record. Bone Modification, 2, 61-71.

Njau, J. and Gilbert, H. 2016. Standardizing terms for crocodile-induced bite marks on bone surfaces in light of the frequent bone modification equifinality found to result from crocodile feeding behavior, stone tool modification, and trampling. FOROST (Forensic Osteology) Occasional Publications, 3:1-13.

Olsen, S.L. and P. Shipman, 1988. Surface Modification on Bone: Trampling versus Butchery. Journal of Archaeological Science, 15, 535-553.

Rozada, L., Allain, R., & Tournepiche, J. F. (2018). Trampling experiments on bones in fine and soft sediments. Quaternaire. Revue de l'Association française pour l'étude du Quaternaire, 29(1), 39-44.

Siviero, B. C., Rega, E., Nick, K. E., & Chadwick, A. V. (2025). Analysis of pseudopathologies in Edmontosaurus annectens bones: taphonomic implications from biogenetic and diagenetic bone alterations from a Cretaceous bonebed in the Lance Formation, Wyoming. Journal of Vertebrate Paleontology, e2600392.

--Andrew A. Farke

**********

6. PLOS authors have the option to publish the peer review history of their article (what does this mean?). If published, this will include your full peer review and any attached files.

If you choose “no”, your identity will remain anonymous but your review may still be made public.

**Do you want your identity to be public for this peer review?**   For information about this choice, including consent withdrawal, please see our Privacy Policy.

Reviewer #1: No

Reviewer #2: No

Reviewer #3: **Yes:**  Rodolfo A. Coria

Reviewer #4: No

Reviewer #5: **Yes:**  T. Alexander Dececchi

Reviewer #6: **Yes:**  Andrew A. Farke

**********

[NOTE: If reviewer comments were submitted as an attachment file, they will be attached to this email and accessible via the submission site. Please log into your account, locate the manuscript record, and check for the action link "View Attachments". If this link does not appear, there are no attachment files.]

To ensure your figures meet our technical requirements, please review our figure guidelines: https://journals.plos.org/plosone/s/figures

You may also use PLOS’s free figure tool, NAAS, to help you prepare publication quality figures: https://journals.plos.org/plosone/s/figures#loc-tools-for-figure-preparation.

NAAS will assess whether your figures meet our technical requirements by comparing each figure against our figure specifications.

Attachment Submitted filename: PONE-D-25-67537-B. Siviero.pdf

Attachment Submitted filename: PONE-D-25-67537_reviewer.pdf

Attachment Submitted filename: PONE-D-25-67537TAD.pdf

# Author comment: Author response to Decision Letter 1

**DOI:** 10.1371/journal.pone.0351939.r002

3 May 2026

Response to Reviewers

REPLIES TO PDF COMMENTS DIRECTLY INTO THE MANUSCRIPT

A) PONE-D-2567537_reviewer.pdf:

• Comment on page 11 pdf file

Page 3 original manuscript

“most of”

Authors’ response: edited according to suggestion (line 82)

• Comment on page 38 pdf file

Figure 1 in original manuscript

“I suggest including a map of the United States showing the state of Wyoming, then the actual state of WY and finally the studied area. In the latter include at least one value of latitude and one value of longitude. Also highlight any road or reference in the map. So the final figure will have 3 sub images: United states, WY and the quarry map.”

Authors’ response: The authors decided to remove figure one according to another reviewer’s suggestion to remove the original figure 1 (with the map). Instead, we used the reference Synder et al. (2020) since they already discussed and illustrated the same bonebed and quarries location with maps and other figures. Article is published in PLoS One (see reference below).

Snyder K, McLain M, Wood J, Chadwick A. Over 13,000 elements from a single bonebed help elucidate disarticulation and transport of an Edmontosaurus thanatocoenosis. PLoS One. 2020 May 21;15(5): e0233182

B) PONE-D-2567537-B.Siviero.pdf:

• Comment on page 11 pdf file

Page 3 original manuscript

“use a comma instead of semicolon”

Authors’ response: edited according to suggestion (revised manuscript with tracking line 83).

• Comment on page 12 pdf file

Page 4 original manuscript

“There are many other ichnotaxa that are not footprints and the features mentioned in this sentence. It is better to delete this sentence”

Authors’ response: edited according to suggestion. “it is helpful to compare different ichnotaxa to determine perpetrator” (revised manuscript with tracking line 98-99)

• Comment on page 13 pdf file

Page 5 original manuscript

“Are there any examples of these in the literature?”

Authors’ response: references added into the paragraph according to comment and some text modification (see lines 103-114 in the revised manuscript with tracking). New references were also added into the reference page and order of references by number was modified to accommodate new additional references.

• Comment on page 13 pdf file

Page 5 original manuscript

“In conclusion,…”

Authors’ response: edited to “thus” for a better flow since section was edited according to overall suggestions of reviwers (revised manuscript with tracking line 115).

• Comment on page 14 pdf file

Page 6 original manuscript

“Shouldn't it be Loma Linda University Medical Center?”

Authors’ response: edited according to suggestion (revised manuscript with tracking line 142).

• Comment on page 16 pdf file

Page 8 original manuscript

“It's not clear what you mean by this. What context? Are you referring to anatomical, sedimentary or something else? What is shape in this case? What is bone association here?”

Authors’ response: edited to clarify the statement according to suggestion (revised manuscript with tracking lines 172-174).

• Comments on pages 17 and 18 pdf file

Pages 9 and 10 original manuscript

“see comment below on the use of verbs in present and past.”

“Some traces are described in present tense (like in Ribs) and others in past tense (like here). It should be consistent throughout the text.”

Authors’ response: modified according to suggestion to present tense when applicable suggestion (revised manuscript with tracking line 236).

• Comment on page 22 pdf file

Page 14 original manuscript

“I suppose you want to say Probably trace maker identification by ichnotaxa”

Authors’ response: edited according to suggestion (revised manuscript with tracking line 322).

• Comment on page 28 pdf file

Page 20 original manuscript

“This seems strange statement. Right before, you state that you did NOT find any lesions affecting cancellous bone, so, why do you say that you may be underestimating? That possibility undermines your previous bold statement. I don't see the logic here.”

Authors’ response: edited section to clarify concept based on the comment by reviewer including additional references (revised manuscript with tracking lines 474-476).

• Comment on page 30 pdf file

Page 22 original manuscript

“shearing?”

Authors’ response: edited according to suggestion (revised manuscript with tracking line 533).

C) PONE-D-2567537TAD.pdf:

• Comments on page 11 pdf file

Page 3 original manuscript

“Well except the ones referenced above. Also, maybe this is just me, but I think another pass can tighten this section up as it feels a little.....flabby. If you don't agree thats ok, but I just would say maybe do another pass and tighten this a little.” (line 58)

“To be fair they mention the size difference (small theropod vs big a larger pterosaur) and others like Hone et al. 2010 mention the condition of the teeth as also supporting this.” (line 62)

“So this would also include Currie and Jacobsen's data too right? Why not introduce that study in this paragraph or figure a way to combine this paragraph into the earlier one.” (line 64)

Authors’ response: paragraph was modified and rewritten according to comments and suggestions from this reviewer and other reviewers’ comments below (revised manuscript with tracking lines 56-76).

• Comments on page 11 pdf file

Page 3 original manuscript

“maybe just indicate which of the above refs you used for this”

Authors’ response: section of the paragraph was modified to adjust content based on the reviewer’s suggestion and accurately use the reference for the 1st description of tooth traces, and also to accommodate for other reviewers’ comments below (revised manuscript with tracking lines 56-76).

• Comments on page 12 pdf file

Page 4 original manuscript

“Maybe condense this paragraph as I feel it is a little long and a could use a bit of a trim to make it more punchy.”

Authors’ response: section of the paragraph was rewritten to accommodate to the best we could the suggestion including suggestions from other reviewers (revised manuscript with tracking lines 56-76).

• Comments on page 12 pdf file

Page 4 original manuscript

“The second line says this, and I like how it phrases it better. Maybe remove this line.”

Authors’ response: edited according to suggestion.

• Comments on page 13 pdf file

Page 5 original manuscript

“Again, combine with paragraph above to make a single, more punchy one that gets the point across.”

Authors’ response: edited according to suggestion and paragraph was rewritten to also include the suggestions above from page 4 in the original manuscript and comments from other reviewers (revised manuscript with tracking lines 100-117).

• Comments on page 15 pdf file

Page 7 original manuscript

“maybe not predator, but just call it a trace maker as if it is scavenging. Especially as the term "predator" has ecological implications, maybe not use that unless you are really confident it was a predation event by that trace maker”

Authors’ response: agree with comment. Sentence was modified to reflect the authors’ intention (revised manuscript with tracking lines 147-148).

• Comments on page 16 pdf file

Page 8 original manuscript

“Maybe put this section after the section dealing with more confidently asserted tooth traces?”

Authors’ response: the authors decided to initially discuss what was not a tooth trace (from our selected fossils) and then focus on the fossils that we determined to represent actual tooth traces based on our experience on the more questionable ones.

• Comments on page 20 pdf file

Page 12 original manuscript

“move this up, so when these taxa are referenced later there is context for the reader.”

Authors’ response: thank you for the suggestion. Authors decided to maintain the original order. We believe that first describing each specimen by its morphology and, when applicable, its potential ichnotaxa, was better since the reader has already been introduced to ichnotaxa in the introduction. In a later section in the results, we prefer to focus on a few selected specimens that actually have preserve tooth-trace ichnotaxa to finalize results on potential tooth trace maker. See also Reply to reviewer #5 comments below.

REPLIES TO ADDITIONAL COMMENTS BY REVIEWERS

D) Reply to reviewer #1 comments:

(Comments by reviewer in black)

“The manuscript is very well written, with clear explanations and well-illustrated. The limitations of interpretation are well considered and explained. Figure 10 and S1 Fig seem to be the same. You should move S2 Fig and S3 Fig into the main text rather than the Supplementary section.”

Authors’ response: Figure S1 in the original manuscript is supplemental review of tooth traces classification according to literature review. Figure 10 in the original manuscript (revised manuscript is now Figure 9) includes every lesion that resembles a tooth trace. It incorporates information from figure S1 and additional lesions/depressions to take into consideration when identifying or classifying tooth traces. Authors decided to maintain the supplemental material figure S1 as in the original manuscript since the is just a visual review of the described literature review. Figures S2 and S3 were also maintained in the supplemental material since they are an indirect part of this article and are just examples for the content discussed but still necessary to be in supplemental material since it adds to the interpretation.

E) Reply to reviewer #2 comments:

(Comments by reviewer in black)

“The study presents results that appear to be original and not previously published, and the manuscript does not show evidence of direct duplication of figures or text from earlier peer-reviewed publications. The research question is incremental rather than groundbreaking, but this is acceptable under PLOS ONE’s scope.

However, the manuscript would benefit from a clearer positioning relative to closely related prior studies, particularly to better articulate what is novel about the present contribution beyond the presentation of tooth traces. The Abstract Introduction should more explicitly state how this work advances existing knowledge rather than reiterating established patterns. For example, only at the end of the mauscript it is cited the guide for the identification of tooth traces

488 (Fig 10) to incorporate many different types of bone perforations. In my opinion this is the most relevant part of the MS, since is a true contribution that enhances previous guideliness (showed in Fig S1).

So, only at the end of the discussion I realized that the MS is adding value other than just a description of tooth bites on some dinosaurs.

I think that of the MS is intended to be published in PLOS ONE, it needs to add value somehow, not only presenting a bit marks in a descriptive way.

I suggest re-shaping the MS focusing on the guide of tooth marks. As I see, the guide is the product of this MS.”

Authors’ response: the original order in the manuscript is how we refined the criteria. We started to look for tooth traces according to the descriptions and soon we realized that context is important and that other tooth trace like perforations are also possible and could be misidentified. This is discussed and presented in order in the manuscript since the refined criteria are the final product of this study. However, additional sentences and edits were included according to suggestions to emphasize the revision of the criteria for tooth traces in the abstract and introduction (revised manuscript with tracking lines 48-52, 100-125) to bring to attention our contribution in not only describing the traces but also in enhancing the guidelines to tooth traces.

“Coupled with this, the figures need to be of better quality (although maybe they lost quality in the PDF, but please, check the original files since they were not provided) and clearly show the mark patterns matched with the guide.”

Authors’ response: PDF version lost definition as indicated by the reviewers.

Figures were submitted according to the guidelines of the journal.

“See the commented PDF for few other comments.”

Authors’ response: Thank you for the suggestion. All comments associated with PDF were addressed above under section “Replies to PDF Comments Directly into the Manuscript”.

F) Reply to reviewer #3 comments:

(Comments by reviewer in black)

“The manuscript constitutes an interesting contribution to understanding certain biological features commonly found in fossil vertebrates. I believe this manuscript does not require major changes.

My only comment regards a sentence in page 14 related to the possible recognition of Nanotyrannus as a juvenile Tyrannosaurus rex. This has been recently discussed by other authors that sufficiently support they are different taxa (see Zanno, L. E., & Napoli, J. G. (2025). Nanotyrannus and Tyrannosaurus coexisted at the close of the Cretaceous. Nature, 1-3). Perhaps this can be relevant to the identification of the tooth marks inflictor.”

Authors’ response: Thank you for the suggestion. Text was edited to reflect the suggestion with in-text references revised. Reference section of the manuscript was also adjusted to reflect these changes (revised manuscript with tracking lines 326-327).

G) Reply to reviewer #4 comments:

(Comments by reviewer in black)

“The submitted MS reports the results of investigation of tooth traces of Edmontosaurus bones from Lance Formation. This topic is more than welcome, because numerous articles have been published in recent years on the examination of bite marks, which in many cases can be used to draw palaeoecological conclusions. However, unfortunately, this manuscript does not contain any new relevant scientific information related to the examination of bite marks, but only uses previously published observations in the examination of their own material. I am not saying that there is no need to mention the examples cited in the manuscript in a publication, but I just wanted to emphasize that the current manuscript does not contain any significant new findings. The conclusion chapter also contains entirely general statements. If the authors disagree with this, it would be justified to formulate the new results listed in the conclusions more precisely, and it would be worthwhile to emphasize in the main text of the article what different or new results have been achieved in the field of bite mark examination. In summary, the article is descriptive in nature. It contains observations, but does not place them in a broader context. However, the results presented in the study may be used as data in other publications, so the editor will decide whether to publish the study.

Authors’ response: Thank you for the comments. Several sections of this manuscript were edited according to suggestions. Discussion and Conclusion sections of the manuscript we edited and added content according to the suggestions.

• There are numerous great scientific publications dealing with bite marks of Mesozoic bones, but only a few studies are mentioned in the publication, which is often based on a rather arbitrary selection. I believe that the authors do not refer to fundamental works published on the subject (e.g. Fiorill, 1991; Carpenter, 1998; Jacobsen, 1998; Farlow and Holtz, 2002; Avilla et al., 2004; Buffetaut et al., 2004; Hone and Rauhut, 2009; Fiorelli, 2010; Longrich et al., 2010; Milàn et. al., 2010; Schwimmer, 2010; Bell, et al., 2012; Boyd et al., 2013, Botfalvai et al 2014 and references therein). I recommend taking a critical look at the publication mentioned and the references listed.

Authors’ response: Thank you for the recommendation. We have incorporated several of the recommended publications where relevant to illustrate tooth traces and animal interactions within the introduction section of this manuscript. As this is not a review paper on tooth traces and animal interactions, we have not expanded these sections extensively but have included selected citations where appropriate to strengthen our introduction. Some of these references were also added into the discussion section of the manuscript, when applicable to the interpretations of tooth trace inflictors. See revised manuscript

Attachment Submitted filename: Response to Reviwers.docx

# Peer review: Decision Letter 1

**DOI:** 10.1371/journal.pone.0351939.r003

**Authors:** Furqan A. Shah

4 Jun 2026

Identification of tooth traces from a Cretaceous (Maastrichtian) Edmontosaurus annectens bonebed in the Lance Formation, Wyoming, U.S.A.

PONE-D-25-67537R1

Dear Dr. C. T. Siviero,

We’re pleased to inform you that your manuscript has been judged scientifically suitable for publication and will be formally accepted for publication once it meets all outstanding technical requirements.

Within one week, you’ll receive an e-mail detailing the required amendments. When these have been addressed, you’ll receive a formal acceptance letter and your manuscript will be scheduled for publication.

An invoice will be generated when your article is formally accepted. Please note, if your institution has a publishing partnership with PLOS and your article meets the relevant criteria, all or part of your publication costs will be covered. Please make sure your user information is up-to-date by logging into Editorial Manager at Editorial Manager® and clicking the ‘Update My Information' link at the top of the page. For questions related to billing, please contact billing support.

If your institution or institutions have a press office, please notify them about your upcoming paper to help maximize its impact. If they’ll be preparing press materials, please inform our press team as soon as possible -- no later than 48 hours after receiving the formal acceptance. Your manuscript will remain under strict press embargo until 2 pm Eastern Time on the date of publication. For more information, please contact onepress@plos.org.

Kind regards,

Furqan A. Shah

Academic Editor

PLOS One

Additional Editor Comments (optional):

Reviewers' comments:

Reviewer's Responses to Questions

**Comments to the Author**

1. If the authors have adequately addressed your comments raised in a previous round of review and you feel that this manuscript is now acceptable for publication, you may indicate that here to bypass the “Comments to the Author” section, enter your conflict of interest statement in the “Confidential to Editor” section, and submit your "Accept" recommendation.

Reviewer #1: All comments have been addressed

Reviewer #3: All comments have been addressed

Reviewer #6: (No Response)

**********

2. Is the manuscript technically sound, and do the data support the conclusions?

The manuscript must describe a technically sound piece of scientific research with data that supports the conclusions. Experiments must have been conducted rigorously, with appropriate controls, replication, and sample sizes. The conclusions must be drawn appropriately based on the data presented.

Reviewer #1: Yes

Reviewer #3: Yes

Reviewer #6: Yes

**********

3. Has the statistical analysis been performed appropriately and rigorously?

Reviewer #1: N/A

Reviewer #3: Yes

Reviewer #6: N/A

**********

4. Have the authors made all data underlying the findings in their manuscript fully available?

The PLOS Data policy requires authors to make all data underlying the findings described in their manuscript fully available without restriction, with rare exception (please refer to the Data Availability Statement in the manuscript PDF file). The data should be provided as part of the manuscript or its supporting information, or deposited to a public repository. For example, in addition to summary statistics, the data points behind means, medians and variance measures should be available. If there are restrictions on publicly sharing data—e.g. participant privacy or use of data from a third party—those must be specified.

Reviewer #1: Yes

Reviewer #3: Yes

Reviewer #6: Yes

**********

5. Is the manuscript presented in an intelligible fashion and written in standard English?

PLOS ONE does not copyedit accepted manuscripts, so the language in submitted articles must be clear, correct, and unambiguous. Any typographical or grammatical errors should be corrected at revision, so please note any specific errors here.

Reviewer #1: Yes

Reviewer #3: Yes

Reviewer #6: (No Response)

**********

6. Review Comments to the Author

Please use the space provided to explain your answers to the questions above. You may also include additional comments for the author, including concerns about dual publication, research ethics, or publication ethics. (Please upload your review as an attachment if it exceeds 20,000 characters)

Reviewer #1: (No Response)

Reviewer #3: I believe the authors have fullfilled all comments and reviews. I have no further comments hoping to see this ms out soon.

Reviewer #6: The authors have adequately addressed my comments from the previous version of this manuscript. I have only a handful of minor edits to suggest - at this point, I do not need to see the manuscript again.

Comments:

- Add the Griffin et al. paper also, relative to Nanotyrannus as a valid taxon (10.1126/science.adx8706)

- line 118, line 158 - replace "monospecific" with "monodominant" as to the bonebed type, to ensure consistency throughout

- check italics for all genus/species names (esp. in references - e.g., reference 38 and others)

--Andrew A. Farke

**********

7. PLOS authors have the option to publish the peer review history of their article (what does this mean?). If published, this will include your full peer review and any attached files.

If you choose “no”, your identity will remain anonymous but your review may still be made public.

**Do you want your identity to be public for this peer review?**   For information about this choice, including consent withdrawal, please see our Privacy Policy.

Reviewer #1: **Yes:**  Raúl Esperante

Reviewer #3: **Yes:**  Rodolfo A. Coria

Reviewer #6: **Yes:**  Andrew A. Farke

**********

# Editor report: Acceptance letter

**DOI:** 10.1371/journal.pone.0351939.r004

**Authors:** Furqan A. Shah

PONE-D-25-67537R1

PLOS One

Dear Dr. C. T. Siviero,

I'm pleased to inform you that your manuscript has been deemed suitable for publication in PLOS One. Congratulations! Your manuscript is now being handed over to our production team.

At this stage, our production department will prepare your paper for publication. This includes ensuring the following:

* All references, tables, and figures are properly cited

* All relevant supporting information is included in the manuscript submission,

* There are no issues that prevent the paper from being properly typeset

You will receive further instructions from the production team, including instructions on how to review your proof when it is ready. Please keep in mind that we are working through a large volume of accepted articles, so please give us a few days to review your paper and let you know the next and final steps.

Lastly, if your institution or institutions have a press office, please let them know about your upcoming paper now to help maximize its impact. If they'll be preparing press materials, please inform our press team within the next 48 hours. Your manuscript will remain under strict press embargo until 2 pm Eastern Time on the date of publication. For more information, please contact onepress@plos.org.

You will receive an invoice from PLOS for your publication fee after your manuscript has reached the completed accept phase. If you receive an email requesting payment before acceptance or for any other service, this may be a phishing scheme. Learn how to identify phishing emails and protect your accounts at https://explore.plos.org/phishing.

If we can help with anything else, please email us at customercare@plos.org.

Thank you for submitting your work to PLOS ONE and supporting open access.

Kind regards,

PLOS ONE Editorial Office Staff

on behalf of

Dr. Furqan A. Shah

Academic Editor

PLOS One
